# Supplementary material for: Acetate supplementation restores cognitive deficits caused by ARID1A haploinsufficiency in excitatory neurons
Source: EMBO Mol Med. 2022 Nov 17;14(12):e15795. doi: 10.15252/emmm.202215795 (PMC9728054; doi:10.15252/emmm.202215795)
Supplement: Supplementary file 1 — Appendix [file EMMM-14-e15795-s004.pdf]

Appendix

Table of contents

Appendix Figure S1-S5
Appendix Table S1-S2

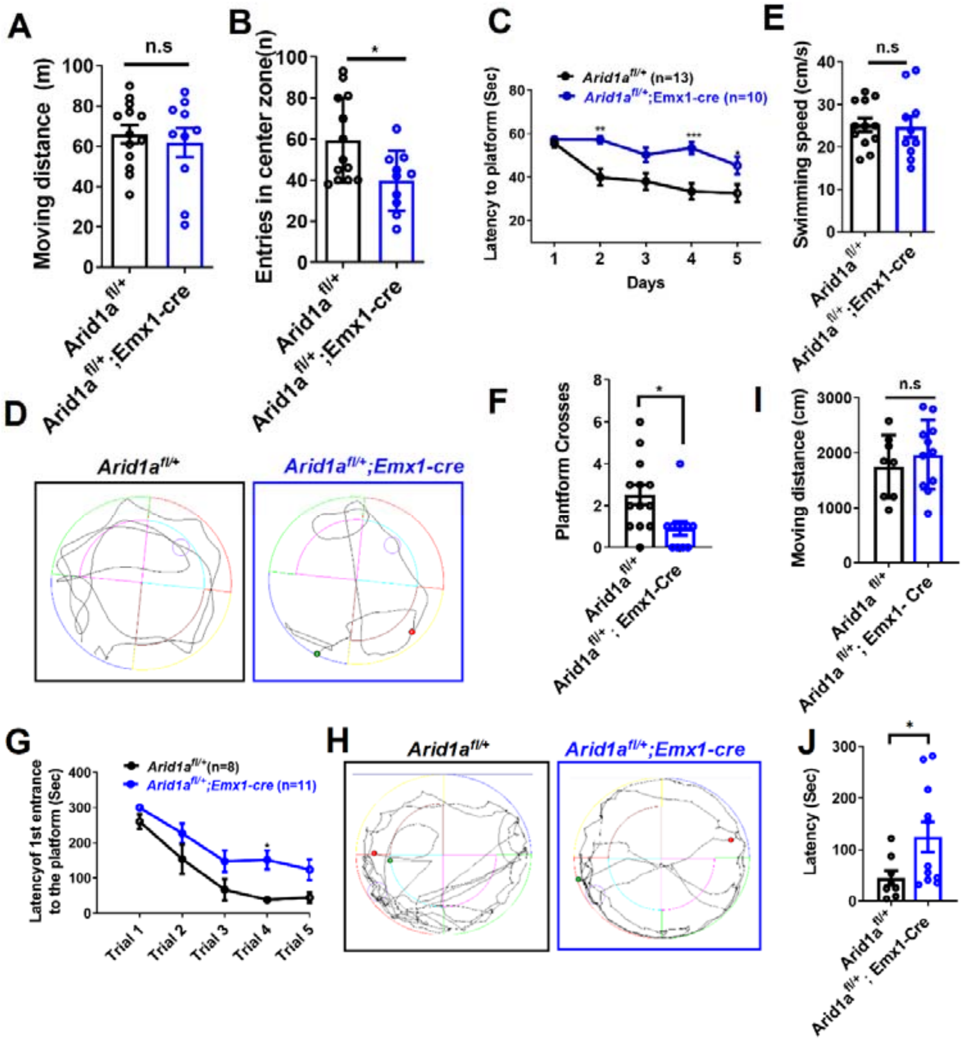

Appendix Figure S1. Haploinsufficiency of *Arid1a* in forebrain leads to impaired learning and memory

A *Arid1a*$^{fl/+}$*; Emx1-Cre* mice had similar locomotion compared to *Arid1a*$^{fl/+}$ littermate mice in an open field test over a 30-min period (*Arid1a*$^{fl/+}$, n=13 mice; *Arid1a*$^{fl/+}$*;Emx1-Cre*, n=10 mice).

B *Arid1a*$^{fl/+}$*;Emx1-Cre* mice entered the center zone less frequently during the 30-min open field test compared to *Arid1a*$^{fl/+}$ littermate mice (*Arid1a*$^{fl/+}$, n=13 mice; *Arid1a*$^{fl/+}$*;Emx1-Cre*, n=10 mice).

C *Arid1a*$^{fl/+}$*;Emx1-Cre mice* spent more time reaching the platform during the 5-day training in the Morris water maze test in the left (*Arid1a*$^{fl/+}$, n=13 mice; *Arid1a*$^{fl/+}$ *;Emx1-Cre*, n=10 mice).

D Representative locomotive patterns of *Arid1a*$^{fl/+}$ and *Arid1a*$^{fl/+}$*;Emx1-Cre* mice in the water maze test.

E *Arid1a*$^{fl/+}$*;Emx1-Cre* mice showed similar swimming speed in Morris water maze test compared to Arid1a$^{fl/+}$ mice (*Arid1a*$^{fl/+}$, n=13 mice; *Arid1a*$^{fl/+}$*;Emx1-Cre* , n=10 mice; n.s=non-significant).

F *Arid1a*$^{fl/+}$*;Emx1-Cre* mice showed less platform crossing in the Morris water maze test (*Arid1a*$^{fl/+}$, n=13 mice; *Arid1a*$^{fl/+}$*;Emx1-Cre*, n=10 mice; $*P < 0.05$, unpaired two-tailed *t*-test).

G *Arid1a*$^{fl/+}$*; Emx1-Cre* mice spent more time finding the platform during the 5-day training in the Barnes maze test (*Arid1a*$^{fl/+}$, n=8 mice; *Arid1a*$^{fl/+}$*;Emx1-Cre*, n=11 mice).

H Representative tracing pathway of *Arid1a*$^{fl/+}$ and *Arid1a*$^{fl/+}$*;Emx1-Cre* mice in the Barnes maze test.

I *Arid1a*$^{fl/+}$*;Emx1-Cre* mice showed similar moving distance in Morris water maze test compared to GFP mice (*Arid1a*$^{fl/+}$, n=8 mice; *Arid1a*$^{fl/+}$*;Emx1-Cre*, n=11 mice; n.s=non-significant).

J *Arid1a*$^{fl/+}$*;Emx1-Cre* mice showed longer latency finding the escape box in the Barnes maze test (*Arid1a*$^{fl/+}$, n=8 mice; *Arid1a*$^{fl/+}$*;Emx1-Cre*, n=11 mice; $**P < 0.05$, unpaired two-tailed *t*-test).

Data information: Data represent means ± SEM. In (A, B, E, F, I, J)$*P$ <0.05, n.s=non-significant, unpaired two-tailed *t*-test. In C, $P > 0.9999$ (day 1), $**P < 0.01$(day 2),

$P = 0.0594$ (day 3), ***$P < 0.001$(day 4), *$P < 0.05$ (day 5), two-way ANOVA with Bonferroni post hoc test. In G, $P > 0.9999$ (Trial 1), $P = 0.2823$ (Trial 2), $P = 0.1829$ (Trial 3), *$P < 0.05$ (Trial 4), P =0.1982 (Trial 5), two-way ANOVA with Bonferroni post hoc test.

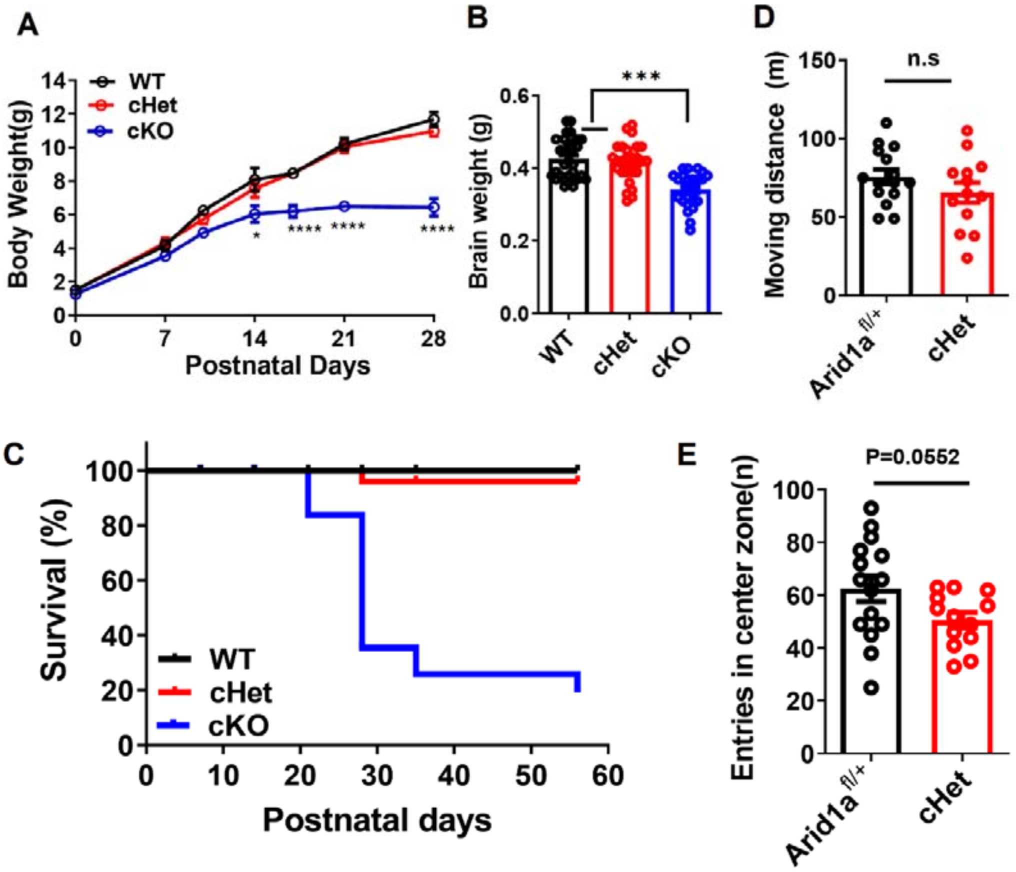

Appendix Figure S2. Loss of *Arid1a* in excitatory neurons leads to high postnatal lethality

A Quantitative comparison of body weight in P0-P28 from WT, cHet and cKO mice. n=4-14.

B Brain weight of the indicated genotypes at P17.

C Kaplan–Meier graph shows survival curves of the WT, cHet and cKO mice groups. Mice in the homozygous (cKO) group exhibited significantly decreased survival (blue) relative to the

other groups. A majority of *Arid1a*$^{fl/fl}$*;Nex-Cre* mice died by P28.

D cHet mice had similar locomotion compared to *Arid1a*$^{fl/+}$ littermate mice in open field test over a 30-min period (*Arid1a*$^{fl/+}$, n=15 mice; cHet, n=13 mice).

E cHet mice had less entry into the center zone during the 30-min open field test compared to *Arid1a*$^{fl/+}$ littermate mice (*Arid1a*$^{fl/+}$, n=15 mice; cHet, n=13 mice).

Data information: Data represent means ± SEM. In A, *$P < 0.05$ (day 14), ***$P < 0.001$ (day 17), ***$P<0.001$ (day 21), ***$P < 0.001$(day 28) versus the other groups, unpaired Student's t-test. ***$P < 0.001$. In C, ***$P < 0.001$, two-tailed log-rank tests. In (B, D, E), ***$P < 0.001$, n.s=non-significant, unpaired two-tailed *t*-test.

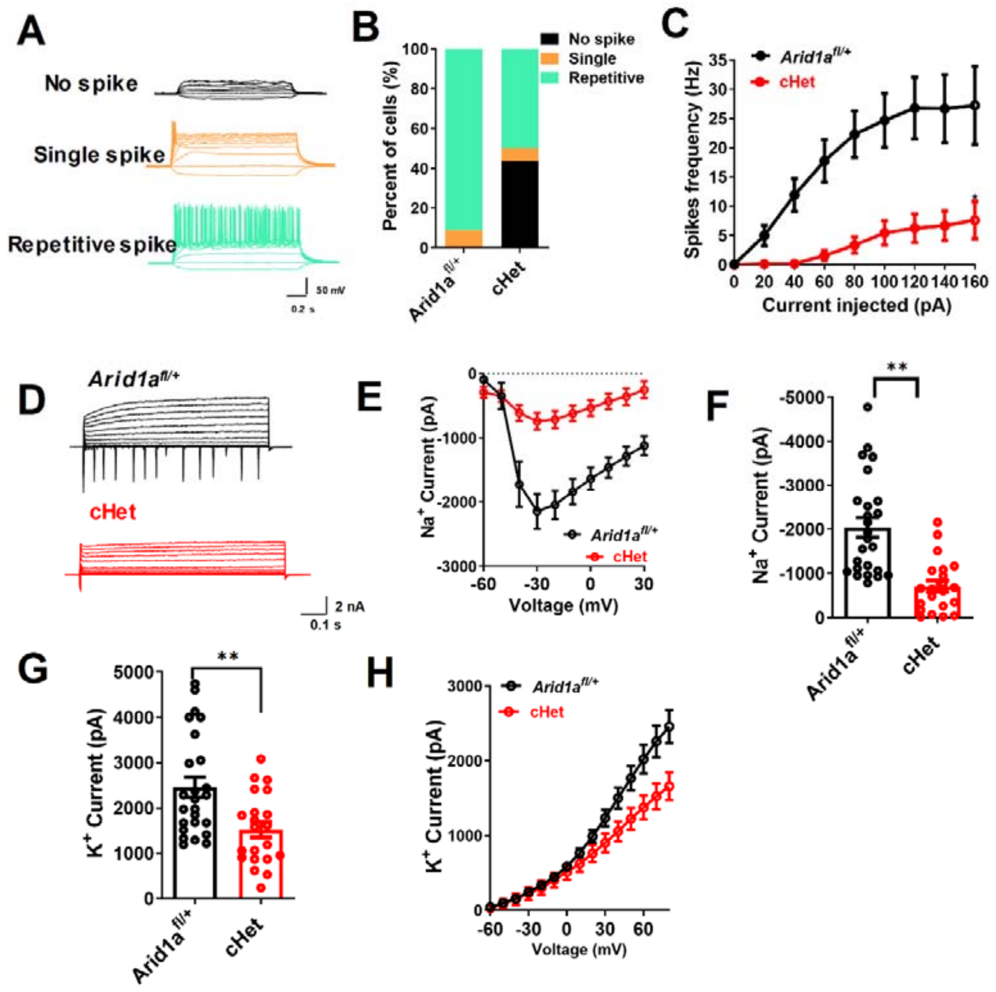

Appendix Figure S3. Electrophysiological defects in hippocampal neurons with *Arid1a* haploinsufficiency

A Representative traces of membrane potential responding to step depolarization by current injection steps from -10 pA to +60 pA in 10pA increments. Membrane potential was current-clamped at around -65 mV. Representative traces were displayed by *Arid1a*$^{fl/+}$ neurons.

B Quantification of the neuron maturity by recorded AP firing patterns in *Arid1a*$^{fl/+}$ and cHet hippocampal CA1 neurons.

C Mean input/output relationship in WT and cHet hippocampal CA1 neurons.

D Representative hyperpolarizing inward current density traces of total current in *Arid1a*$^{fl/+}$ and cHet hippocampal CA1 neurons.

E, F Averaged current-voltage relationship (I-V curves) for Na+ currents, recorded from *Arid1a*$^{fl/+}$ and cHet hippocampal CA1 neurons.

G, H Average current-voltage relationship (I-V curves) for K+ currents, recorded from *Arid1a*$^{fl/+}$ and cHet hippocampal CA1 neurons.

Data information: Data represent means ± SEM. In (C, F, G), *$P < 0.05$, **$P < 0.01$(unpaired two-tailed *t*-test).

)

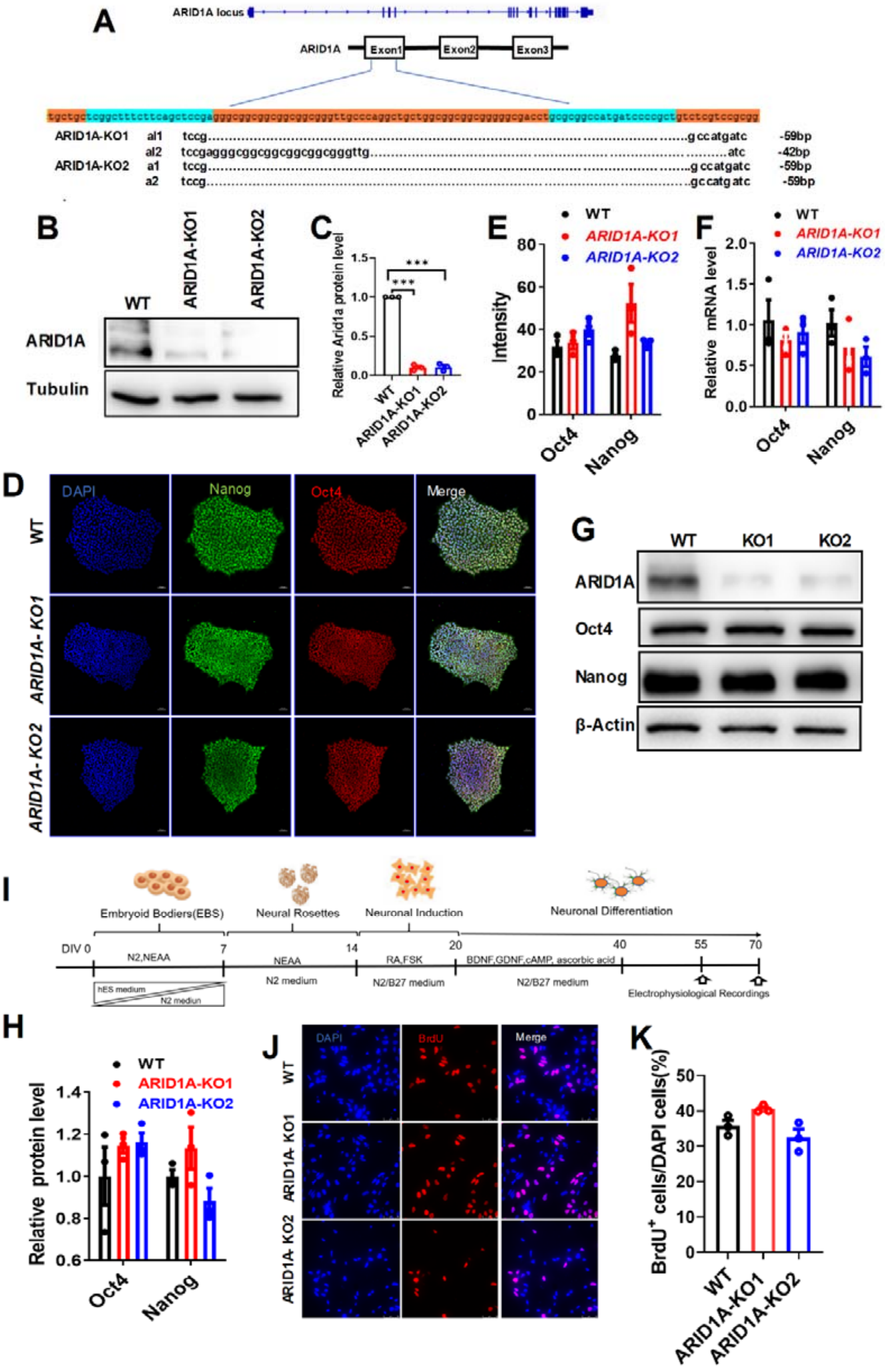

A
ARID1A locus
ARID1A
Exon1
Exon2
Exon3
ARID1A-KO1 al1 tccg..........gccatgatc -59bp
al2 tccgagggcggcggcggcggcgggttg..........atc -42bp
ARID1A-KO2 a1 tccg..........gccatgatc -59bp
a2 tccg..........gccatgatc -59bp
B
WT
ARID1A-KO1
ARID1A-KO2
ARID1A
Tubulin
C
Relative Arid1a protein level
E
Intensity
Oct4
Nanog
WT
ARID1A-KO1
ARID1A-KO2
F
Relative mRNA level
D
DAPI
Nanog
Oct4
Merge
G
KO1
KO2
β-Actin
I
Embryoid Bodiers(EBS)
Neural Rosettes
Neuronal Induction
Neuronal Differentiation
DIV 0
N2,NEAA
NEAA
RA,FSK
BDNF,GDNF,cAMP, ascorbic acid
hES medium
N2 medium
N2/B27 medium
Electrophysiological Recordings
H
Relative protein level
J
BrdU
K
BrdU+ cells/DAPI cells(%)

Appendix Figure S4. The deletion of *ARID1A* has no effect on the pluripotency of hESCs

A Strategy for using CRISPR/Cas9 to complete knock out human *ARID1A* in H9 hESCs. The sgRNA sequences are labeled in blue and PAM recognition sequences are highlighted in red. Dotted line indicates the deletion of allele 1 (al1) and allele 2 (al2).

B Western blot analysis of ARID1A protein expression levels. β-Tubulin was used as a loading control.

C Quantification of the density of the ARID1A protein bands by normalization to the intensity of β-Tubulin bands. n=3.

D Representative images of immunostaining expression of OCT4 (green) and Nanog(red) in WT and ARID1A KO clone. Scale bar, 50 μm.

E Relative fluorescence intensities of OCT4 and Nanog were elevated upon the loss of ARID1A in the hESC clones. $n = 3$.

F Real-time PCR analysis of OCT4 and Nanog mRNA levels in WT and ARID1A KO clones. The amount of mRNA was normalized to GAPDH levels. n = three experimental replicates.

G Western blot analysis of OCT4 and Nanog protein expression in WT and ARID1A KO clones. Tubulin was used as a loading control.

H Quantification of the density of the OCT4 and Nanog protein bands by normalization to the intensity of β-Actin bands.

I Schematic protocol of neural differentiation of hESCs. RA, retinoic acid. BDNF, brain-derived neurotrophic factor. GDNF, glial cell line-derived neurotrophic factor.

J WT and ARID1A KO hESCs immunostained positive for BrdU.

K Bar chart displayed percentage of the BrdU-positive cells. $n = 3$ biological replicates. Scale

bar, 50 μm.

Data information: Data represent means ± SEM. In C, ***P<0.001(unpaired two-tailed t-test).

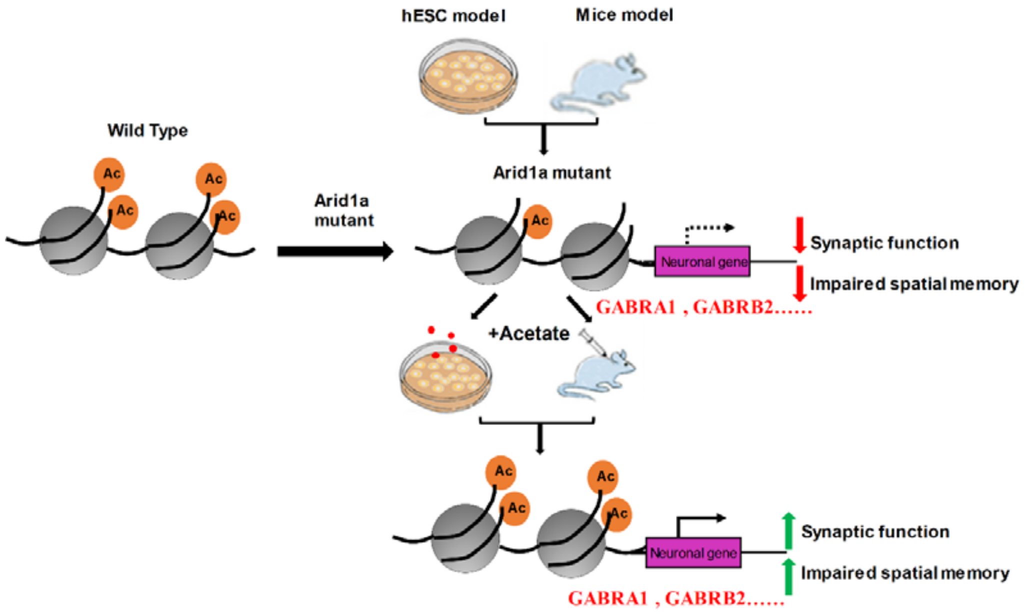

Appendix Figure S5. The working model of ARID1A in excitatory neurons

ARID1A haploinsufficiency in excitatory neurons disrupts histone modifications in genes essential for the functions of excitatory neurons and cognition in mice. After acetate supplementation can restore the expression of key neuronal genes, including GABRA1, GABRB2, the deficits of cognition and neuronal morphology are rescued in both murine and human neurons.

Appendix TableS1. Primers for Genotyping

Genotyping	Sequence
ARID1A-F:	TGG GCA GGA AAG AGT AAT GG
ARID1A-R:	AAC ACC ACT TTC CCA TAG GC
Nex-Cre P1	GAGTCCTGGAATCAGTCTTTTTC
Nex-Cre P2	AGAATGTGGAGTAGGGTGAC
Nex-Cre P3	CCGCATAACCAGTGAAACAG
Emx1-Cre P1	GCGGTCTGGCAGTAAAAACTATC
Emx1-Cre P2	GTGAAACAGCATTGCTGTCACTT
Emx1-Cre P3	AAGGTGTGGTTCCAGAATCG
Emx1-Cre P4	CTCTCCACCAGA AGGCTGAG

Appendix Table S2. Real-Time PCR primers

RT-PCR	Forward sequence (5'-3')	Reverse sequence (5'-3')
Gabrb2	ATGTCGCTGGTTAAAGAGACG	CTGCCACTCGGTTGTCCAAA
Gabra1	AAAAGTCGGGGTCTCTCTGAC	CAGTCGGTCCAAAATTCTTGTGA
Slitrk1	GAAGGGGACTTACACGTAGACT	AGTGAGGGAATTGCCATGCAG
Cacna2d1	CTGCTGGCCTTGACTCTGAC	CACTCCACTTGCTGTTTTTGC
Fzd6	ATGGAAAGGTCCCCGTTTCTG	GGGAAGAACGTCATGTTGTAAGT
Rp1	CCTGTAGTGGCTAAACGCATC	CAGCAGAGCGTCAAAAGTCTTA
Tnf	GGTGCCTATGTCTCAGCCTCTT	GCCATAGAACTGATGAGAGGGAG
Vgf	CTTTGACACCCTTATCCAAGGCG	GCTAATCCTTGCTGAAGCAGGC
Lrfn1	GGCATCCGTATGTACCAAGTGC	GCCAGGTCATTCACTAGGAAGG
C1ql1	AGTATGTGGGCAGACCTCTGCA	TCCAGATGCAGGATCACGCTGT
Lrrtm4	TTCCTCGTCTCTTCAACCTGCG	CAGGCTCGATTGCTTGGATGTC
App	TCCGTGTGATCTACGAGCGCAT	GCCAAGACATCGTCGGAGTAGT

Cntn2	CTCCAGCAGAATCCGCACTAAG	CTCCATTCTGGTACTCTCGTGAC
Tac1	TAATGGGCAAGCGGGATGCTGA	CCATTAGTCCAACAAAGGAATCTG
Faim2	GCAACTTACCTGACTCTGGCTTG	TGGACAGCATCCCAGTGAGGTA
Mapk8	CGCCTTATGTGGTGACTCGCTA	TCCTGGAAAGAGGATTTTGTGGC
Bok	TTCATGCCCTGGTTGACTGCCT	AAGCCAGGATCTGTGCTGACCA
Agap2	CGCAACCTATGGGCTCAATGTG	GCTACAGGAGTAGATGCAGCTG
Epha7	GATGTTGCCACACTTGAGGAAGC	ATGATGGTCCCTGCTACAGCCA
Ripk1	GACTGTGTACCCTTACCTCCGA	CACTGCGATCATTCTCGTCCTG
Bbc3	ACCGCTCCACCTGCCGTCAC	ACGGGCGACTCTAAGTGCTGC
Dapk3	AAGCAGGAGACGCTGACGAACA	AATGCTCCAGGCTCTGTGCGAT
Bax	AGGATGCGTCCACCAAGAAGCT	TCCGTGTCCACGTCAGCAATCA
