# Supplementary material for: Acetate supplementation restores cognitive deficits caused by ARID1A haploinsufficiency in excitatory neurons
Source: EMBO Mol Med. 2022 Nov 17;14(12):e15795. doi: 10.15252/emmm.202215795 (PMC9728054; doi:10.15252/emmm.202215795)
Supplement: Supplementary file 2 — Expanded View Figures PDF [file EMMM-14-e15795-s001.pdf]

Expanded View Figures

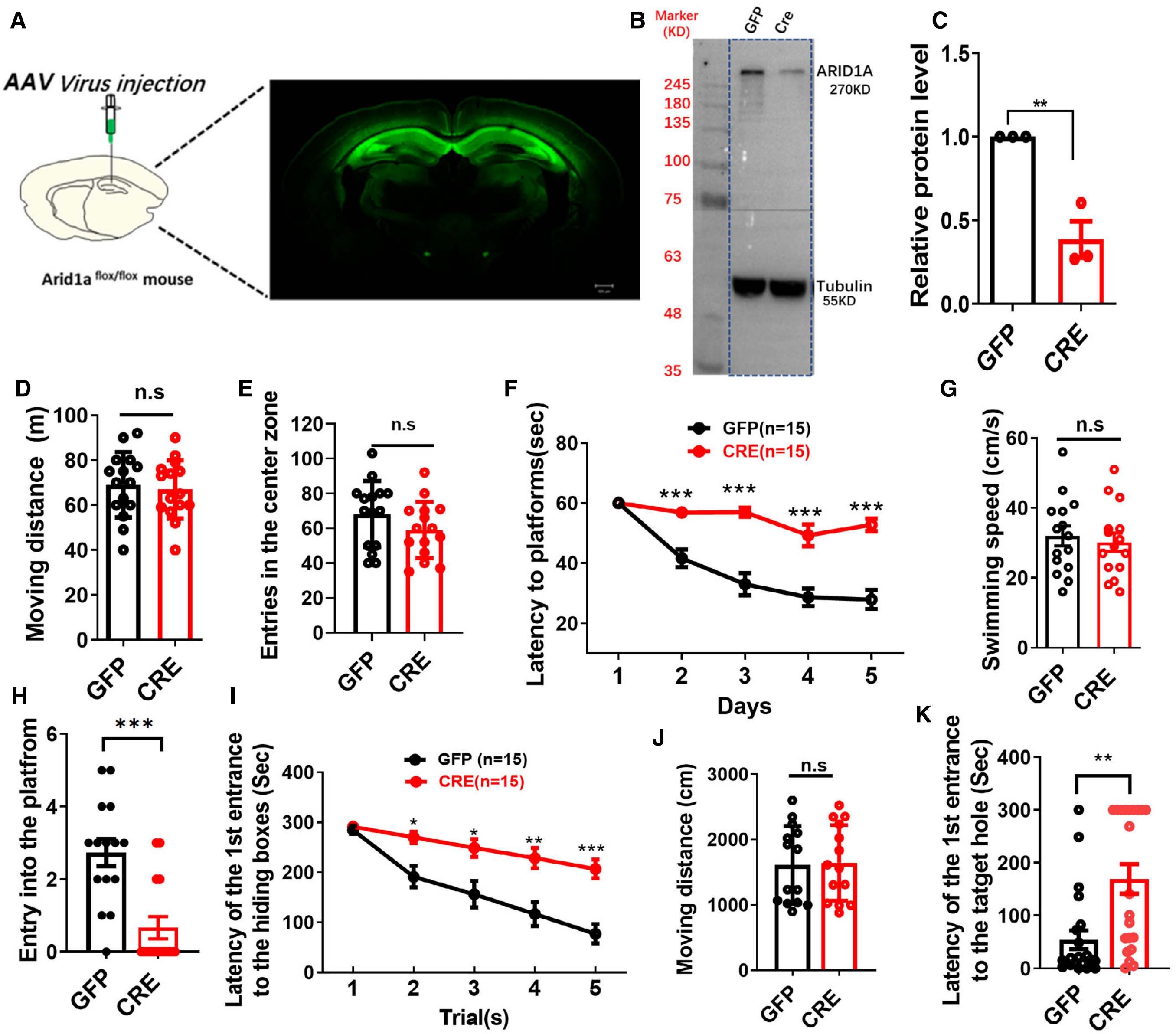

Figure EV1.

Figure EV1. Reduced expression of *Arid1a* exhibits impaired learning and memory disability.

A Schematic illustrating injection of AAV-CRE-GFP or AAV-GFP control virus in adult *Arid1a*$^{fl/fl}$ hippocampus (left), representative images showing GFP expression in the hippocampus after 1-month viral injection (right). Scale bar, 500 μm.
B Representative Western blot images for ARID1A expression. ARID1A expression is downregulated in the hippocampus after AAV-*Cre* injection. Tubulin was used as a loading control.
C Quantification of ARID1A protein levels in hippocampal tissues from GFP and AAV-*Cre* mice ($n = 3$ mice).
D AAV-*Cre* mice had comparable locomotion to GFP littermate mice in open field test over a 30-min period (AAV-GFP, $n = 15$ mice; AAV-*Cre*, $n = 15$ mice).
E AAV-*Cre* mice had comparable entries into the center zone during a 30-min open field test compared to GFP littermate mice (AAV-GFP, $n = 15$ mice; AAV-*Cre*, $n = 15$ mice; n.s., nonsignificant).
F AAV-*Cre* mice spent more time reaching the platform during the 5-day training in the Morris water maze test (AAV-GFP, $n = 15$ mice; AAV-*Cre*, $n = 15$ mice).
G AAV-*Cre* mice showed similar swimming speed in the Morris water maze test compared to GFP mice (AAV-GFP, $n = 15$ mice; AAV-*Cre*, $n = 15$ mice).
H AAV-*Cre* mice crossed the platform less frequently in the Morris water maze test (AAV-GFP, $n = 15$ mice; AAV-*Cre*, $n = 15$ mice).
I AAV-*Cre* mice spent more time finding the escape box during 5-day training in the Barnes maze test (AAV-GFP, $n = 15$ mice; AAV-*Cre*, $n = 15$ mice).
J AAV-*Cre* mice showed similar moving distances in the Morris water maze test compared to GFP mice (AAV-GFP, $n = 15$ mice; AAV-*Cre*, $n = 15$ mice).
K AAV-*Cre* mice showed longer latency when finding the escape box in the Barnes maze test (AAV-GFP, $n = 15$ mice; AAV-*Cre*, $n = 15$ mice).

Data information: Data represent means ± SEM. In (C–E), $^{**}P < 0.01$, n.s. = nonsignificant (unpaired two-tailed *t*-test). In (F), $P > 0.9999$ (day 1), $^{***}P < 0.001$ (day 2), $^{***}P < 0.001$ (day 3), $^{***}P < 0.001$ (day 4), $^{***}P < 0.001$ (day 5; two-way ANOVA with Bonferroni *post hoc* test). In (G, H), n.s. = nonsignificant, $^{***}P < 0.001$(unpaired two-tailed *t*-test). In (I), $P > 0.9999$ (Trial1), $^{*}P < 0.05$(Trial 2), $^{*}P < 0.05$ (Trial 3), $^{**}P < 0.01$ (Trial 4), $^{***}P < 0.001$ (Trial 5; two-way ANOVA with Bonferroni *post hoc* test). In (J, K), n.s. = nonsignificant, $^{**}P < 0.01$(unpaired two-tailed *t*-test).
Source data are available online for this figure.

Figure EV2. Supplementation of Acetate rescues neuronal morphology deficits caused by *Arid1a* haploinsufficiency in excitatory neurons.

A Change in the body weight of mice after chronic treatment with ethyl acetate ($n = 5$ mice per group).
B Separate channels of the triple immunostainings staining of DAPI (blue), H3K27ac (green), and Vgult1 (red) in forebrain tissues of *Arid1a*$^{fl/+}$ and cHet mice, respectively. IF staining was performed on 40-μm thick floating sections. Relative fluorescence intensities of H3K27ac decreased upon the loss of Arid1a in the cortex. Scale Bar, 20 μm.
C Open field test in *Arid1a*$^{fl/+}$ and *cHet* mice treated with vehicle or Acetate. *Arid1a*$^{fl/+}$ and cHet mice treated with vehicle or Acetate had similar locomotion in open field tests over a 30-min period ($n = 15$ mice per group).
D *Arid1a*$^{fl/+}$ and cHet mice treated with vehicle or Acetate displayed similar entry tendencies into the center zone during a 30-min open field test, ($n = 15$ mice per group).
E The digitized trace of Golgi-stained coronal sections in the hippocampal CA1 region of adult (2-months-old) Vehicle or Acetate-treated cHet mice. Scale Bar, 50 μm.
F Quantification of total dendritic length from dendritic tree reconstructions shown in (E)., at least 15 neurons from $n = 3$ mice per group.
G Sholl analysis of dendritic branching complexity in the total dendrites of vehicle or Acetate-treated cHet mice, at least 15 neurons from $n = 3$ mice per group.
H Sholl analysis of dendritic branching complexity in the basal and apical dendrites of vehicle or Acetate-treated cHet mice, at least 15 neurons from $n = 3$ mice per group.

Data information: Data represent means ± SEM. In (C, D), n.s. = nonsignificant, unpaired two-tailed *t*-test. In (F), $^{**}P < 0.01$(apical), $^{**}P < 0.01$(total; unpaired two-tailed *t*-test). In G, $^{*}P < 0.05$ (100 μm), $^{*}P < 0.05$ (110 μm; two-way ANOVA with Bonferroni *post hoc* test). In (H), $^{*}P < 0.05$ (80 μm), $^{*}P < 0.05$ (90 μm), $^{*}P < 0.05$ (100 μm), $^{**}P < 0.01$ (110 μm), $^{*}P < 0.05$ (120 μm), $^{*}P < 0.05$ (130 μm; two-way ANOVA with Bonferroni *post hoc* test).

 ©2022 The Authors

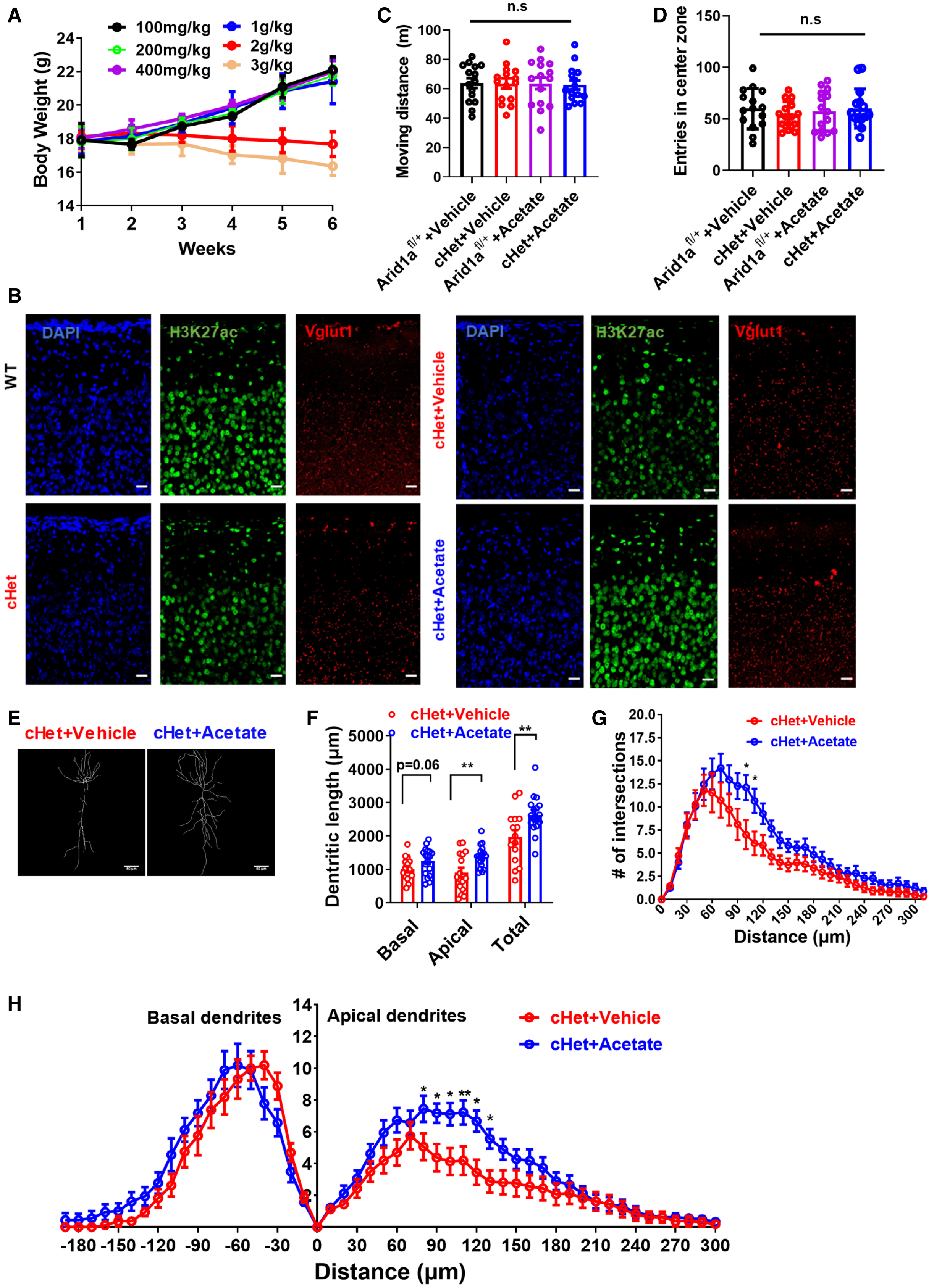
A
100mg/kg
200mg/kg
400mg/kg
1g/kg
2g/kg
3g/kg
Body Weight (g)
Weeks
C
n.s
Moving distance (m)
D
n.s
Entries in center zone
Arid1a fl/+ +Vehicle
cHet+Vehicle
Arid1a fl/+ +Acetate
cHet+Acetate
B
DAPI
H3K27ac
Vglut1
WT
cHet
cHet+Vehicle
cHet+Acetate
E
cHet+Vehicle
cHet+Acetate
F
cHet+Vehicle
cHet+Acetate
Dentritic length (μm)
p=0.06
**
**
Basal
Apical
Total
G
cHet+Vehicle
cHet+Acetate
# of intersections
Distance (μm)
H
Basal dendrites
Apical dendrites
cHet+Vehicle
cHet+Acetate
Distance (μm)

Figure EV2.

Figure EV3. Altered gene expression profile caused by the haploinsufficiency of *Arid1a*.

A Schematic of RNA-seq experiment. Left, coronal section showing the distribution of excitatory neurons with red fluorescence of tdTomato.
B The principal component analysis (PCA) of RNA-seq.
C GO functional clustering of downregulated targets in Arid1a-haploinsufficiency transcriptome.
D KEGG pathway analysis of upregulated targets in Arid1a-haploinsufficiency transcriptome.
E Principal component analysis (PCA) results of H3K27ac ChIP-seq data based on enrichment signals at peak regions.
F Left, heatmaps displaying enhanced (5061) and reduced (1899) H3K27ac peaks from ChIP-seq analysis in the *Arid1a*$^{fl/+}$ vs cHet mice. Right, Gene ontology (GO) functional clustering of genes that were upregulated and downregulated to identify biological processes regulated by H3K27ac ChIP-seq. The unit of the color key is normalized RPKM.
G Effect of acetate on genome-wide occupancy of H3K27ac as determined by ChIP-seq. The unit of the color key is normalized RPKM.

©2022 The Authors

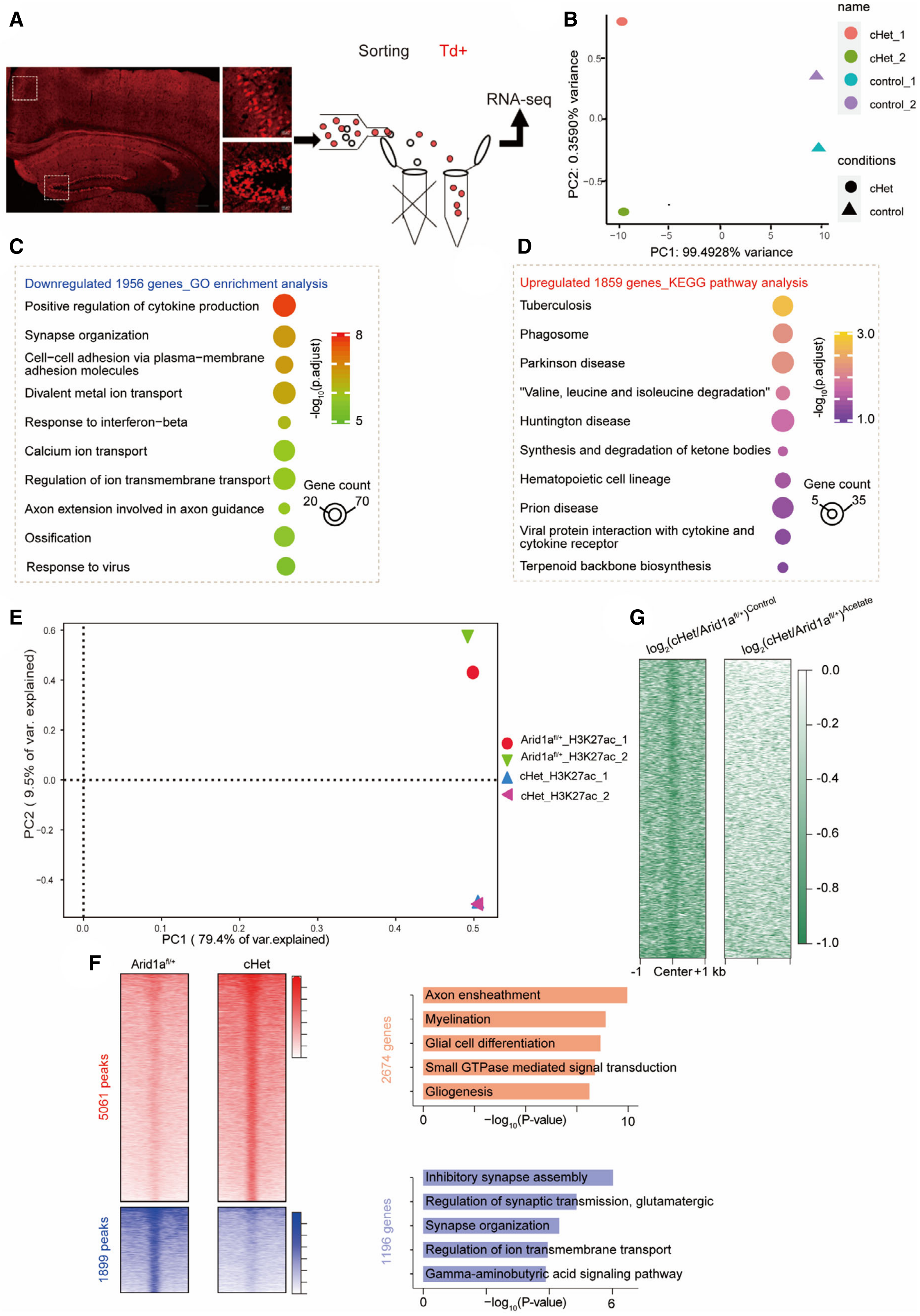
A
Sorting
Td+
RNA-seq
B
name
cHet_1
cHet_2
control_1
control_2
conditions
cHet
control
PC2: 0.3590% variance
PC1: 99.4928% variance
C
Downregulated 1956 genes_GO enrichment analysis
Positive regulation of cytokine production
Synapse organization
Cell−cell adhesion via plasma−membrane adhesion molecules
Divalent metal ion transport
Response to interferon−beta
Calcium ion transport
Regulation of ion transmembrane transport
Axon extension involved in axon guidance
Ossification
Response to virus
-log10(p.adjust)
Gene count
D
Upregulated 1859 genes_KEGG pathway analysis
Tuberculosis
Phagosome
Parkinson disease
"Valine, leucine and isoleucine degradation"
Huntington disease
Synthesis and degradation of ketone bodies
Hematopoietic cell lineage
Prion disease
Viral protein interaction with cytokine and cytokine receptor
Terpenoid backbone biosynthesis
-log10(p.adjust)
Gene count
E
PC2 (9.5% of var. explained)
PC1 (79.4% of var.explained)
Arid1a fl/+_H3K27ac_1
Arid1a fl/+_H3K27ac_2
cHet_H3K27ac_1
cHet_H3K27ac_2
F
Arid1a fl/+
cHet
5061 peaks
1899 peaks
2674 genes
Axon ensheathment
Myelination
Glial cell differentiation
Small GTPase mediated signal transduction
Gliogenesis
−log10(P-value)
1196 genes
Inhibitory synapse assembly
Regulation of synaptic transmission, glutamatergic
Synapse organization
Regulation of ion transmembrane transport
Gamma-aminobutyric acid signaling pathway
−log10(P-value)
G
log2(cHet/Arid1a fl/+)Control
log2(cHet/Arid1a fl/+)Acetate
-1 Center +1 kb

Figure EV3.

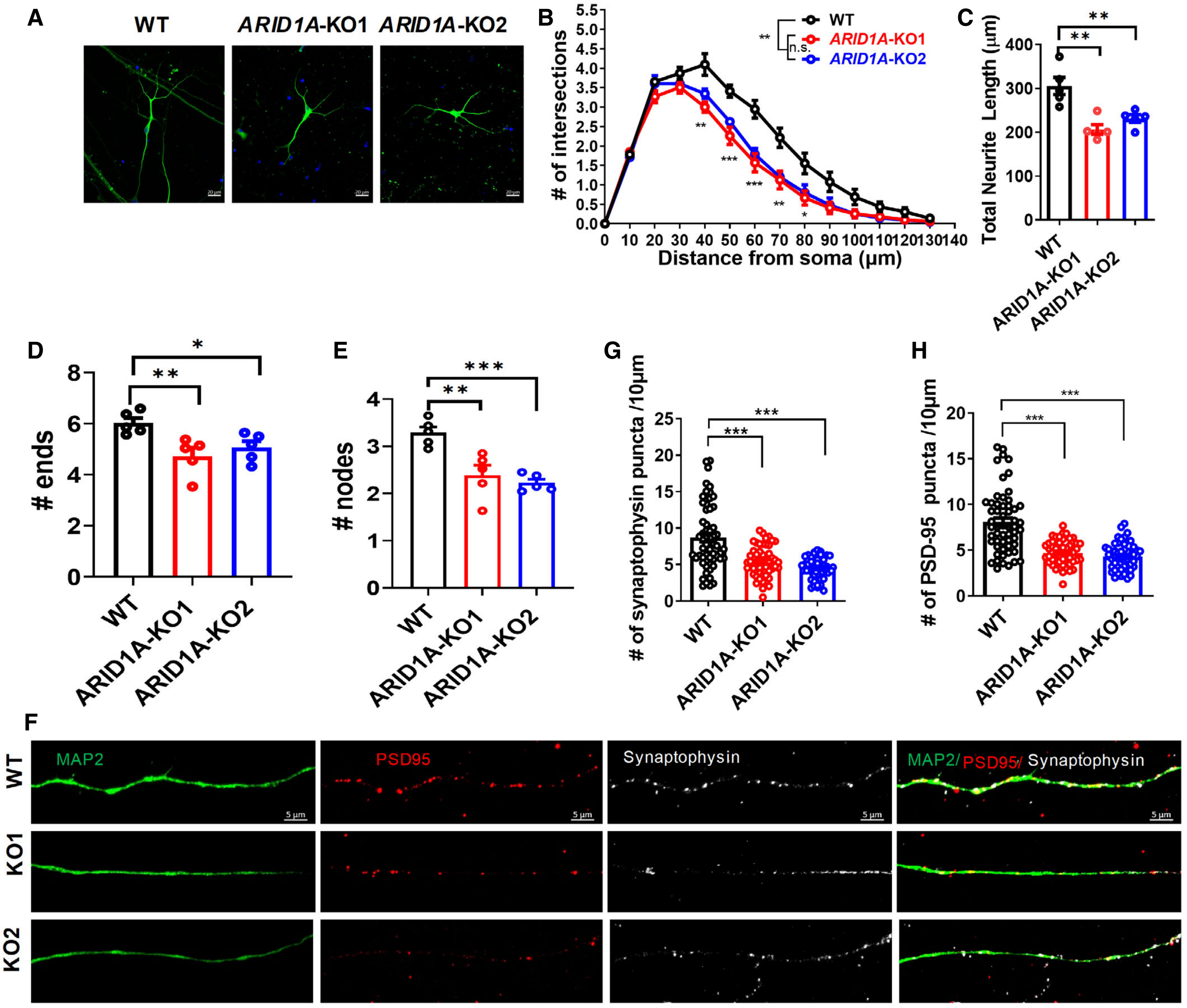

Figure EV4. Loss of ARID1A decreased neurite complexity in hESCs-derived neurons.

A Representative image of neurons derived from WT and *ARID1A* KO hESCs on day 40 of neural differentiation Scale Bar, 20 μm.

B Sholl analysis shows that total neurite length decreased in ARID1A KO hESC-derived neurons on day 40 (*n* = at least 3 independent replicates).

C–E Compared with the WT group, ARID1A KO hESC-derived neurons exhibited decreased dendritic complexity, as shown by reduced length (C), ends (D), and notes (E) (*n* = at least 3 independent replicates).

F Representative images of dendrites (MAP2, green) showing localization of foci of the pre- and postsynaptic protein complexes, synaptophysin, and PSD-95 proteins in WT and ARID1A KO hESC-derived neurons on day 55 Scale Bar, 5 μm.

G, H Quantification of the spine density from synaptophysin (G) and PSD-95 protein (H) stained secondary dendrites of neurons on day 55 were significantly reduced in ARID1A KO hESC on day 55 of neural differentiation (*n* = 3 independent replicates).

Data information: Data represent means ± SEM. In (B), $^{*}P < 0.05$ (40 μm), $^{***}P < 0.01$(50 μm), $^{***}P < 0.01$(60 μm), $^{**}P < 0.01$(70 μm), $^{*}P < 0.05$ (80 μm); ANOVA. In (C), $^{**}P < 0.01$ (KO1), $^{**}P < 0.01$ (KO2); unpaired two-tailed *t*-test. In (D), $^{**}P < 0.01$(KO1), $^{*}P < 0.05$ (KO2); unpaired two-tailed *t*-test. In (E), $^{**}P < 0.01$ (KO1), $^{***}P < 0.001$ (KO2); unpaired two-tailed *t*-test. In (G), $^{****}P < 0.0001$(KO1), $^{****}P < 0.0001$(KO2); unpaired two-tailed *t*-test. In (H), $^{****}P < 0.0001$(KO1), $^{****}P < 0.0001$(KO2), unpaired two-tailed *t*-test.

 ©2022 The Authors

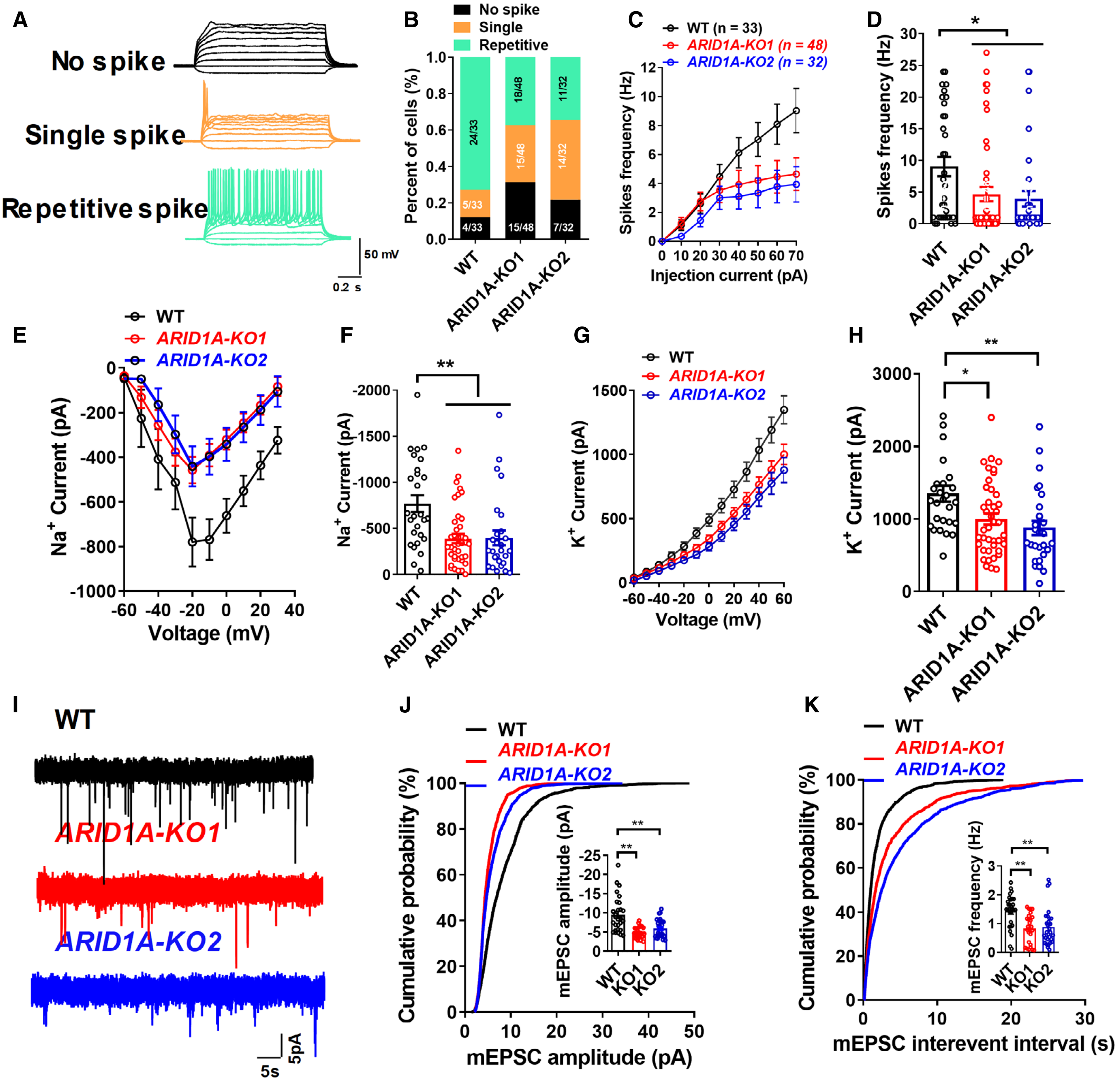

Figure EV5. Loss of ARID1A results in electrophysiological defects and neuronal functions in hESCs-derived neurons.

A Representative traces of membrane potential responding to step depolarization by current injection steps from −10 pA to +60 pA in 10 pA increments. Membrane potential was current-clamped at around −65 mV. Representative traces were displayed by WT neurons.

B Quantification of the neuron maturity by recorded AP firing patterns on day 55 after differentiation ($n \geq 30$ neurons in every group).

C, D Mean input/output relationship during WT and ARID1A hESC-derived neurons on day 55. ($n \geq 30$ neurons in every group).

E, F Averaged current–voltage relationship (I-V curves) for Na+ currents, recorded from hESC-derived neurons ($n \geq 27$ neurons in every group).

G, H Averaged current–voltage relationship (I-V curves) for K+ currents, recorded from hESC-derived neurons ($n \geq 27$ neurons in every group).

I Detection of mEPSCs in whole-cell recordings of WT and ARID1A KO hESC-derived neurons on day 55.

J Amplitude of SC decreased significantly in ARID1A KO hESC-derived neurons ($n \geq 25$ neurons in every group).

K Frequency of mEPSC decreased significantly in ARID1A KO hESC-derived neurons ($n \geq 25$ neurons in every group).

Data information: Data represent means ± SEM. In (D, F, H, J, K), *$P < 0.05$, **$P < 0.01$, unpaired two-tailed t-test.

©2022 The Authors
