# Supplementary figures and images for: Acetate supplementation restores cognitive deficits caused by ARID1A haploinsufficiency in excitatory neurons
Source: EMBO Mol Med. 2022 Nov 17;14(12):e15795. doi: 10.15252/emmm.202215795 (PMC9728054; doi:10.15252/emmm.202215795)

Appendix Figure S4

B

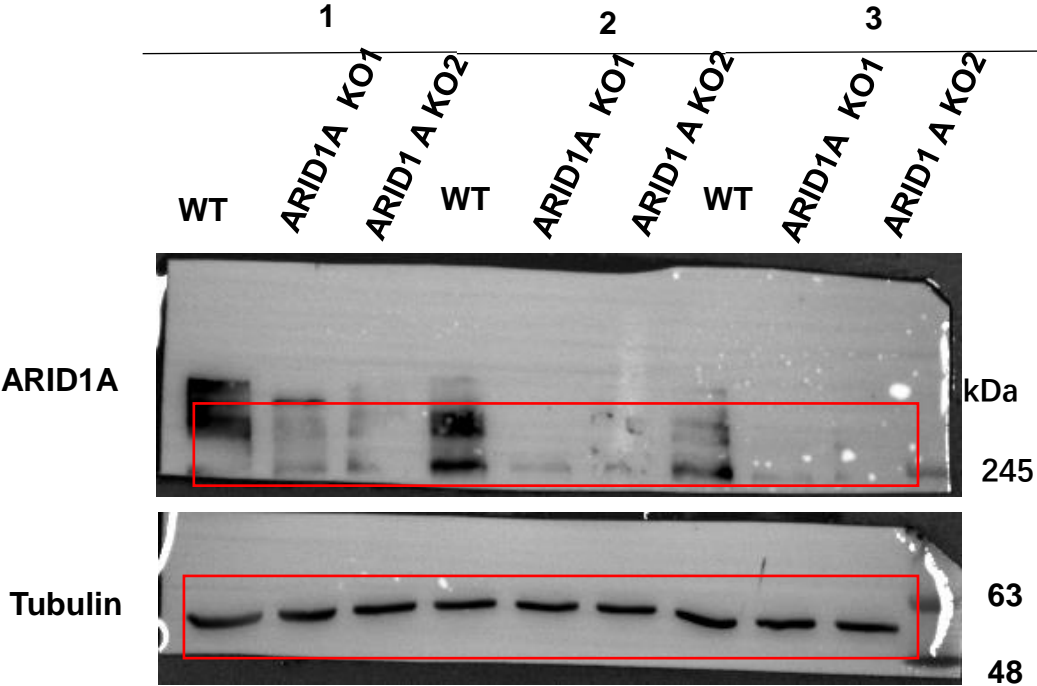

G

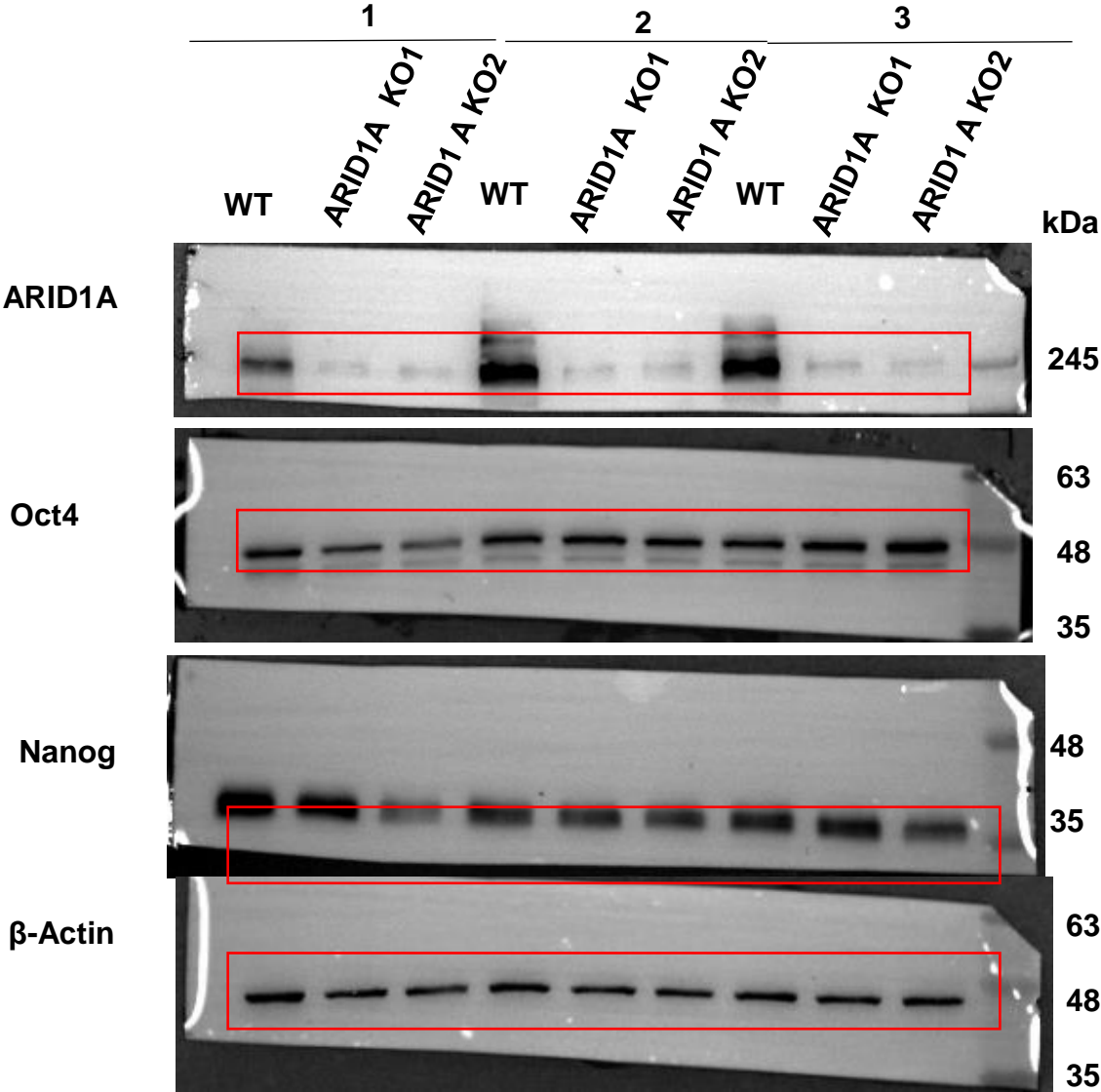

Supplement: Supplementary file 3 — Source Data for Expanded View and Appendix [file EMMM-14-e15795-s006.zip › manuscriptEMM-2022-15795_SourceDataFor ExpandedViewAndAppendixFigure/SourceDataForAppendixFigure S4.pdf]

## Figure EV1B

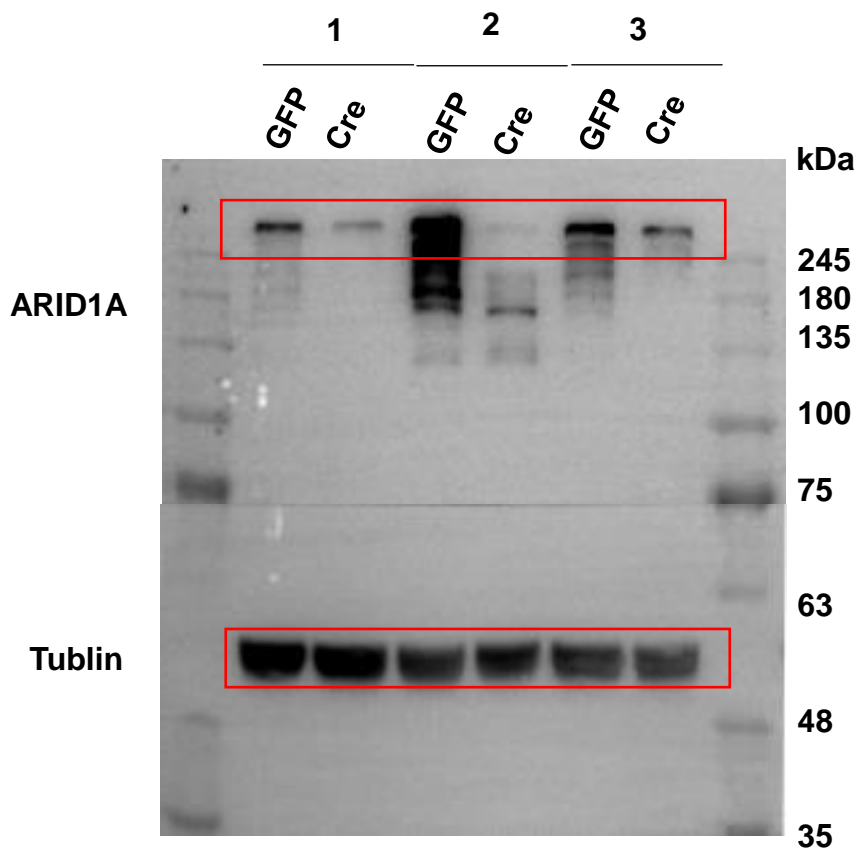

Supplement: Supplementary file 3 — Source Data for Expanded View and Appendix [file EMMM-14-e15795-s006.zip › manuscriptEMM-2022-15795_SourceDataFor ExpandedViewAndAppendixFigure/SourceDataForFigure EV1.pdf]

Figure 1

B

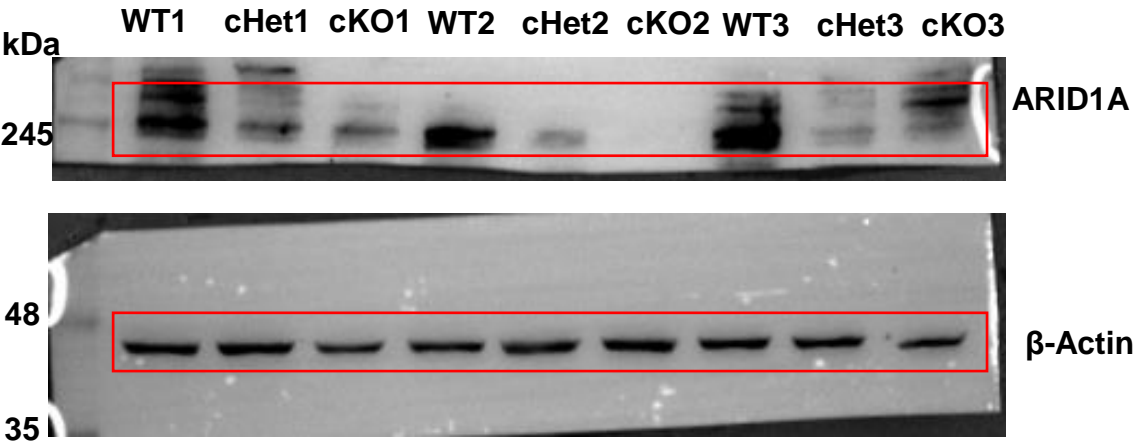

C

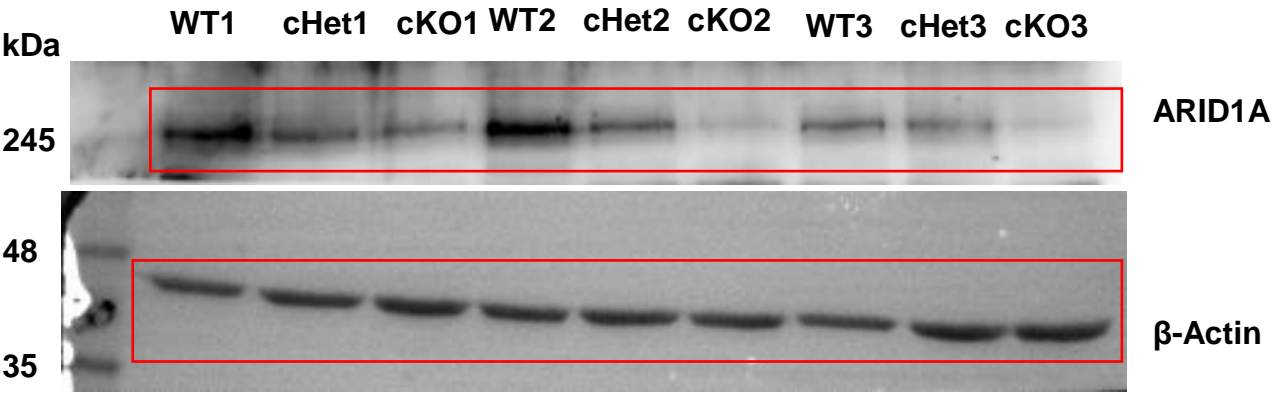

Supplement: Supplementary file 5 — Source Data for Figure 1 [file EMMM-14-e15795-s003.pdf]

### Figure 2G

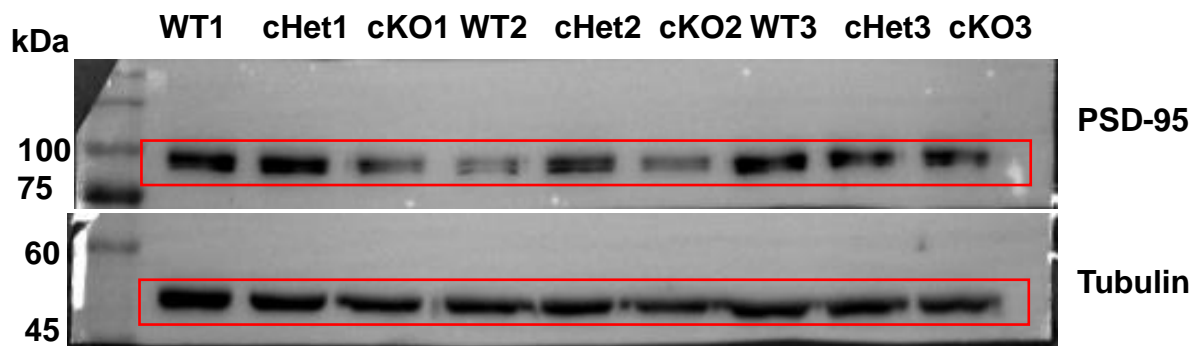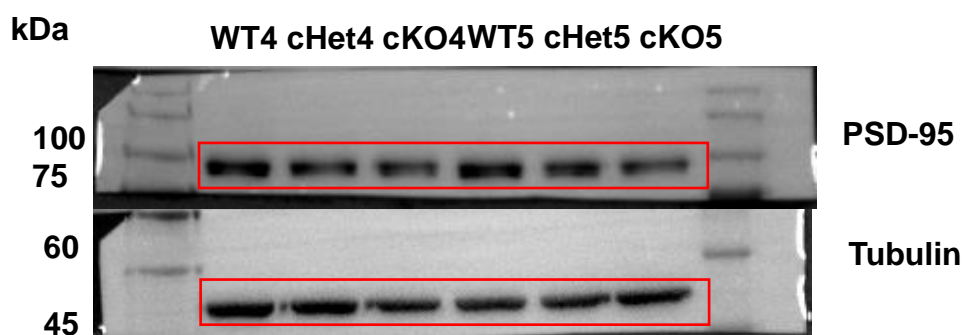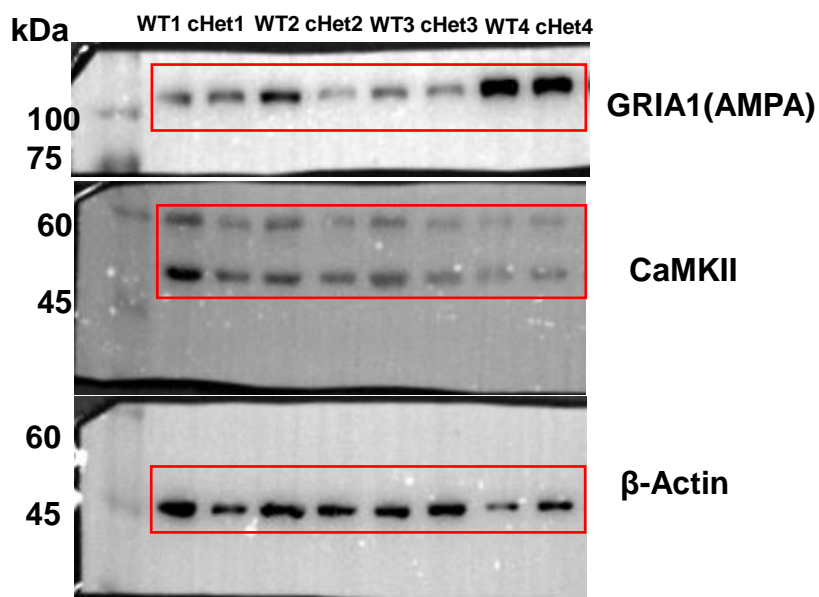

Supplement: Supplementary file 6 — Source Data for Figure 2 [file EMMM-14-e15795-s007.pdf]

Figure 4

A

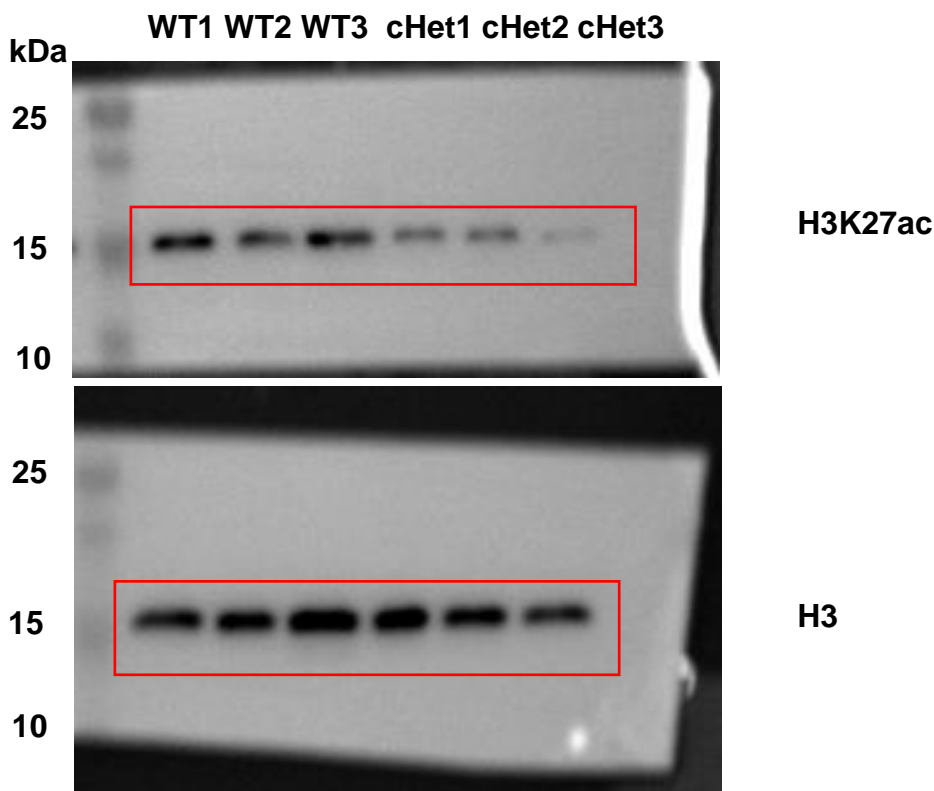

**B**

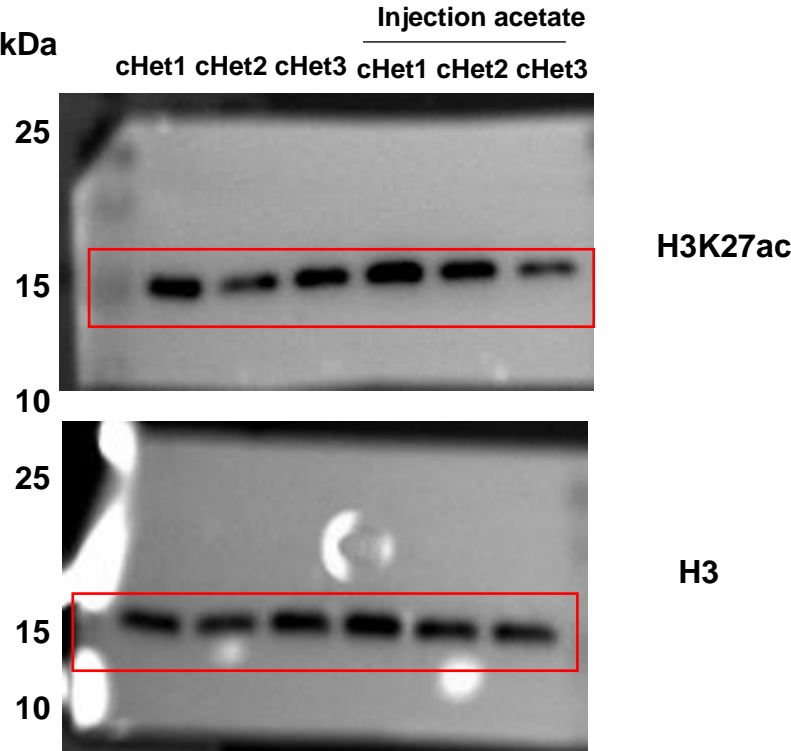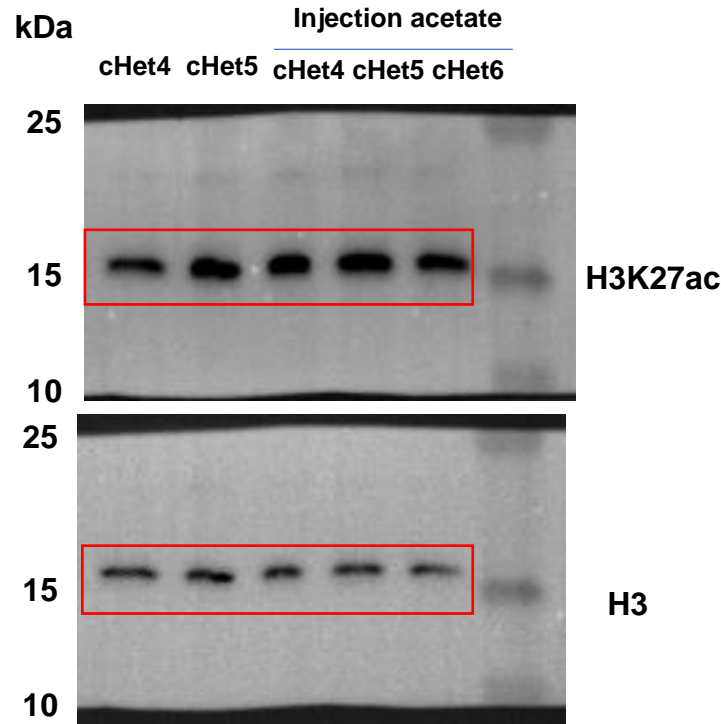

Supplement: Supplementary file 7 — Source Data for Figure 4 [file EMMM-14-e15795-s005.pdf]
