# Supplementary material for: Acetate supplementation restores cognitive deficits caused by ARID1A haploinsufficiency in excitatory neurons
Source: EMBO Mol Med. 2022 Nov 17;14(12):e15795. doi: 10.15252/emmm.202215795 (PMC9728054; doi:10.15252/emmm.202215795)
Supplement: Supplementary file 4 — PDF+ [file EMMM-14-e15795-s002.pdf]

Article

SOURCE DATA | TRANSPARENT PROCESS | OPEN ACCESS

EMBO Molecular Medicine

Acetate supplementation restores cognitive deficits caused by ARID1A haploinsufficiency in excitatory neurons

Pei-Pei Liu[1,2,3,4,†], Shang-Kun Dai[1,2,5,†], Ting-Wei Mi[1,†], Gang-Bin Tang[1], Zhuo Wang[1,2], Hui Wang[1,2], Hong-Zhen Du[1,3,4], Yi Tang[6,*], Zhao-Qian Teng[1,2,3,4,**] & Chang-Mei Liu[1,2,3,4,***]

Abstract

Mutations in AT-rich interactive domain-containing protein 1A (ARID1A) cause Coffin-Siris syndrome (CSS), a rare genetic disorder that results in mild to severe intellectual disabilities. However, the biological role of ARID1A in the brain remains unclear. In this study, we report that the haploinsufficiency of ARID1A in excitatory neurons causes cognitive impairment and defects in hippocampal synaptic transmission and dendritic morphology in mice. Similarly, human embryonic stem cell-derived excitatory neurons with deleted *ARID1A* exhibit fewer dendritic branches and spines, and abnormal electrophysiological activity. Importantly, supplementation of acetate, an epigenetic metabolite, can ameliorate the morphological and electrophysiological deficits observed in mice with *Arid1a* haploinsufficiency, as well as in *ARID1A*-null human excitatory neurons. Mechanistically, transcriptomic and ChIP-seq analyses demonstrate that acetate supplementation can increase the levels of H3K27 acetylation at the promoters of key regulatory genes associated with neural development and synaptic transmission. Collectively, these findings support the essential roles of ARID1A in the excitatory neurons and cognition and suggest that acetate supplementation could be a potential therapeutic intervention for CSS.

Keywords acetate; ARID1A; cognitive deficit; excitatory neurons; SWI/SNF
Subject Category Neuroscience
DOI 10.15252/emmm.202215795 | Received 28 January 2022 | Revised 13 October 2022 | Accepted 14 October 2022 | Published online 17 November 2022
EMBO Mol Med (2022) 14: e15795

Introduction

Intellectual disabilities (ID) are characterized by substantial limitations in both intellectual functioning and adaptive behavior, and their worldwide prevalence has been estimated to be about 1% (Maulik *et al*, 2013). Individuals with ID are often identified early in childhood because of developmental issues, and ID is a major feature of most developmental disorders (Vissers *et al*, 2016). There are no pharmacological and/or genetic treatments for ID, and the current treatments focus on behavioral and educational management and physical therapy. Therefore, it is important to identify causative genetic factors to develop more effective ID therapies.

Coffin-Siris syndrome (CSS), a typical ID disorder, is characterized by developmental delays, intellectual disability, hypoplasia of the fifth fingernails and/or toenails, and multiple organ abnormalities. Exome sequencing studies have identified that CSS is caused by dominant *de novo* mutations in genes encoding individual subunits of the BRM-associated factor (BAF) complex, also known as the switch/sucrose nonfermentable (SWI/SNF)-like chromatin remodeling complex, including *ARID1A*, *ARID1B*, *SMARCA4*, *SMARCB1*, *SMARCA2*, *SMARCE1*, and *PHF6* (Bartsocas & Tsiantos, 1970; Tsurusaki *et al*, 2012; Son & Crabtree, 2014). Individuals with *SMARCE1* mutations have moderate to severe ID (Vasko *et al*, 2021). Patients with *ARID1A* mutations tend to have a broad spectrum of severe ID and internal complications that can lead to early death (Slavotinek *et al*, 2022). Mutations in *SMARCA4*, *SMARCB1*, and *SMARCE1* exert dominant-negative or gain-of-function effects, while *ARID1A*-related variants exert loss-of-function effects (Kosho *et al*, 2014). The BAF complex is widely expressed in cells and organs and plays various roles in embryonic stem cell pluripotency and neuronal lineage specification (Bultman *et al*, 2000; Clapier & Cairns, 2009; Diana & Gerald, 2011; Wilson & Roberts, 2011). ARID1A, also known as BAF250A, is one of

1 State Key Laboratory of Stem Cell and Reproductive Biology, Institute of Zoology, Chinese Academy of Sciences, Beijing, China
2 University of Chinese Academy of Sciences, Beijing, China
3 Institute for Stem Cell and Regeneration, Chinese Academy of Sciences, Beijing, China
4 Beijing Institute for Stem Cell and Regenerative Medicine, Beijing, China
5 School of Life Sciences and Medicine, Shandong University of Technology, Zibo, China
6 Department of Neurology, Innovation Center for Neurological Disorders, Xuanwu Hospital, Capital Medical University, Beijing, China
*Corresponding author. Tel: +86 10 83198277; E-mail: tangyi@xwhosp.org
**Corresponding author. Tel: +86 10 82619699; E-mail: tengzq@ioz.ac.cn
***Corresponding author. Tel: +86 10 82619690; E-mail: liuchm@ioz.ac.cn
[†]These authors contributed equally to this work

©2022 The Authors. Published under the terms of the CC BY 4.0 license.

the main BAF complex subunits. Although human genetic studies suggest that both germ-line mutations and microduplication of *ARID1A* could cause CSS (Tsurusaki *et al*, 2012; Santen *et al*, 2013; Bidart *et al*, 2017), the link between loss-of-function of *ARID1A* and the CSS pathogenesis remain unexplored.

Previous studies have demonstrated that deletion of *Arid1a* in early mouse embryos leads to developmental arrest and the absence of the mesodermal layer (Kim *et al*, 2015). Moreover, *Arid1a* deficiency compromises mouse embryonic stem (ES) cell pluripotency, severely blocks self-renewal and promotes differentiation into primitive endoderm-like cells (Gao *et al*, 2008). This finding highlights the critical role of *Arid1a* in mammalian embryonic development. Although *Arid1a* shows constitutional expression in ES cells, neural progenitors, and postmitotic neurons, it is not known whether ARID1A is incorporated into the BAF complex in a cell-type-specific manner. Importantly, the developmental stage-specific assembly of the BAF complex has been recently reported (Son & Crabtree, 2014). However, it is unclear whether ARID1A has unique functions during neurodevelopment or in specific neurons, or whether there is a link between the dysfunction of ARID1A and the ID phenotype of CSS.

In this study, we established a mouse model with specific *Arid1a* haploinsufficiency in excitatory neurons and demonstrated that the haploinsufficient loss of *Arid1a* leads to the dysfunction of synaptic plasticity, as well as impaired learning and spatial memory. Using CRISPR-CAS9 knockout technology, we further showed that *ARID1A* KO hESC-derived excitatory neurons exhibited a severe reduction in neurite complexity and abnormal synaptic plasticity. Mechanistically, we found that ARID1A haploinsufficiency in excitatory neurons suppressed histone H3 lysine 27 acetylation (H3K27ac) mark at key neuronal genes associated with synapse or cognition (*Gabrb2*, *Gabra1*, *Slitrk1*, *Cacna2d1*). Importantly, an acetate supplement could restore H3K27 acetylation, reverse the expression of neuronal genes, and ameliorate the observed abnormalities in both murine and human neurons carrying the loss of ARID1A. Together, these data establish the genetic basis of the pathophysiological role of ARID1A in CSS and indicate the therapeutic potential of acetate for cognitive disorders with ARID1A haploinsufficiency.

Results

Arid1a haploinsufficiency in excitatory neurons of the forebrain leads to deficits in learning and memory

ARID1A mutations can cause CSS, a rare congenital malformation syndrome with severe neurodevelopmental deficits (Kosho *et al*, 2014), which indicates that ARID1A could be related to cognition. Therefore, we tested whether *Arid1a* knockdown causes a cognitive deficit in young adult mice. We injected the AAV9 virus expressing Cre recombinase to delete *Arid1a* in the hippocampus of 6-week-old *Arid1a*$^{fl/fl}$ mice, while the controls received the virus only expressing GFP (Fig EV1A). Western blot analysis confirmed that ARID1A was successfully knocked down in the hippocampus (Fig EV1B and C). We then investigated whether loss of *Arid1a* was associated with abnormal intellectual behaviors. First, we conducted an open field test and found that there was no difference in the total moving distance, suggesting similar locomotive activities between the control group infected with AAV-GFP and *Arid1a* acute deletion mice with infected AAV-Cre (Fig EV1D). Meanwhile, no anxiety-like behavior was observed in *Arid1a* acute deletion mice, because they had similar entries in the center zone to that of *Arid1a* acute deletion mice (Fig EV1E). Next, we performed the Morris water maze test and Barnes maze test to measure spatial learning and memory in mice. In the Morris water maze test, we found that mice with acute knockdown of *Arid1a* in the hippocampi took a longer time to locate the hidden platform in the training trials (Fig EV1F) and spent less time in the platform zone in the probe test (Fig EV1H). There was no significant difference in swimming speed between the two groups of mice (Fig EV1G). In the Barnes maze test, mice with acute knockdown of *Arid1a* in the hippocampi took a longer time to locate the target hole in the training trials (Fig EV1I) and took a longer time to find the target hole (Fig EV1K), while all mice displayed similar moving distances (Fig EV1J). To further confirm the cognitive phenotype of *Arid1a* deficiency, we crossed *Emx1-Cre* mice with *Arid1a*$^{fl/fl}$ mice to produce *Arid1a*$^{fl/+}$;*Emx1-Cre* mice (Liu *et al*, 2021) for behavioral assays. As we expected, the *Arid1a*$^{fl/+}$;*Emx1-Cre* mice exhibited normal locomotive activities (Appendix Fig S1A and B) but severe cognitive impairment (Appendix Fig S1C–J). These results indicate that *Arid1a* is involved in regulating learning and memory.

Since previous studies demonstrate that *Emx1*-expressing neural progenitors produce cortical excitatory neurons and glia, but not GABAergic neurons (Gorski *et al*, 2002; Kosho *et al*, 2014), we hypothesize that ARID1A regulates cognition in excitatory neurons. To test this hypothesis, we specifically deleted *Arid1a* in excitatory neurons by crossing *Arid1a*$^{fl/fl}$ mice with *Nex-Cre* transgenic mice (Fig 1A; Goebbels *et al*, 2006; Li *et al*, 2017). Western blot (Fig 1B and C) and immunofluorescence staining analyses confirmed that *Arid1a* was deleted in the cortex and hippocampi of *Arid1a*$^{fl/fl}$;*Nex-Cre* (cKO) and the intensity of ARID1A expression significantly decreased *in Arid1a*$^{fl/+}$;*Nex-Cre* (cHet) mice compared to littermate control *Arid1a*$^{fl/+}$(WT; Fig 1D). *Arid1a* cKO mice were born at the Mendelian ratio and were indistinguishable from their wild-type littermates at P0 (Appendix Fig S2A). *Arid1a* cKO mice survived the first 2 weeks after birth (Appendix Fig S2C), albeit with growth retardation and low brain weight (Appendix Fig S2B and C). Approximately 80% of cKO mice died 5 weeks after birth (Appendix Fig S2C). However, cHet mice survived to adulthood and had similar body weights compared to their WT littermates (Appendix Fig S2B and C).

To analyze whether *Arid1a* haploinsufficiency in excitatory neurons affects cognition, we first performed the open field test to rule out the effect of locomotive ability on behavior performance in mice. There was no significant difference in the total moving distance and the entrance to the center zone during the 30-min period between the two groups of mice (Appendix Fig S2D and E), suggesting similar locomotive activities between *Arid1a*$^{fl/+}$ and cHet mice. In the Morris water maze (MWM) test, *Arid1a* cHet mice exhibited considerable delays in finding the platform during the training phase compared to *Arid1a*$^{fl/+}$ mice (Fig 1E). In the subsequent probe test phase, cHet mice showed a significantly longer latency to locate the target and fewer target crossings (Fig 1F and G), indicating that *Arid1a* haploinsufficiency in excitatory neurons affects spatial learning and memory. To further validate impaired spatial learning and memory in cHet mice, we conducted a Barnes maze test and found that cHet mice spent more time searching for the hiding box (Fig 1H

©2022 The Authors

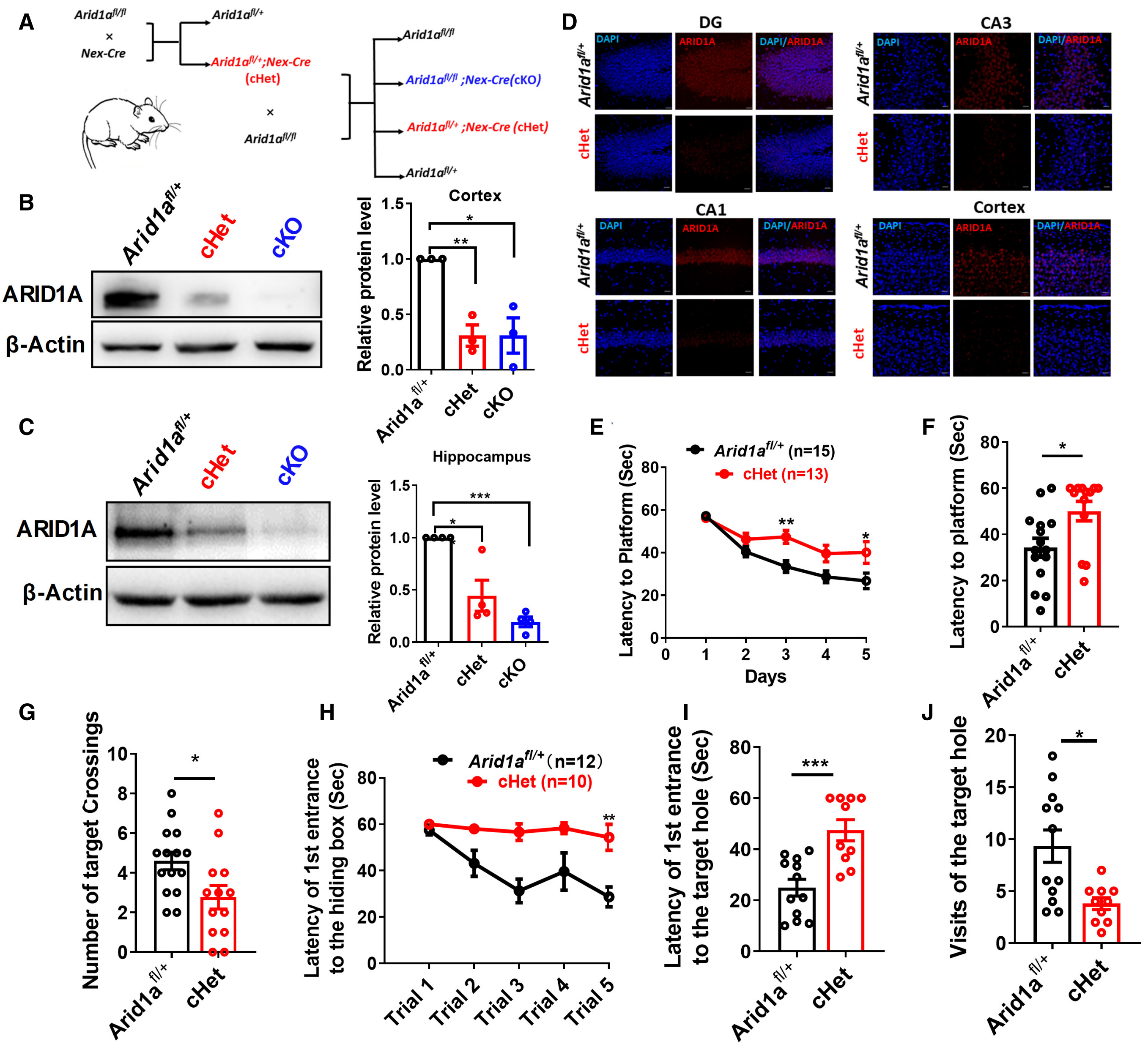

Figure 1. Cognitive defects as a consequence of *Arid1a* haploinsufficiency in mice excitatory neurons.

A Schematic for the production of *Arid1a* knockout mice. By crossing them with *Nex-Cre* mouse lines, we specifically deleted *Arid1a* in neocortex and hippocampus pyramidal neurons and generated heterozygous *Arid1a*$^{fl/+}$ (WT), *Arid1a*$^{fl/+}$;*Nex-Cre* (cHet) mice and *Arid1a*$^{fl/fl}$;*Nex-Cre* (cKO) mice, respectively.

B Left, representative images of Western blot for ARID1A expression in lysates extracted from the cortex of *Arid1a*$^{fl/+}$ (WT), cHet, and cKO mice using the indicated antibodies. Right, quantification of protein levels normalized to β-actin (n = 3 mice per group).

C Left, representative images of Western blot for ARID1A expression in lysates extracted from the hippocampi of *Arid1a*$^{fl/+}$, cHet, and cKO mice. Right, quantification of protein levels which were normalized to β-actin (n = 4 mice per group).

D Immunostaining against ARID1A (red) in the dentate gyrus (DG), cortex, CA1, and CA3 regions of *Arid1a*$^{fl/+}$ and cHet mice. Scale bar, 20 µm.

E cHet mice spent more time reaching the platform during 5-day training in Morris water maze assay compared to the control group mice. *Arid1a*$^{fl/+}$ (n = 15 mice), cHet (n = 13 mice).

F In the probe trial, cHet mice displayed spatial learning disabilities compared with *Arid1a*$^{fl/+}$ mice. *Arid1a*$^{fl/+}$ (n = 15 mice), cHet (n = 13 mice).

G cHet mice undertook fewer platform crossings than *Arid1a*$^{fl/+}$ mice in the Morris water maze assay. *Arid1a*$^{fl/+}$ (n = 15 mice), cHet (n = 13 mice).

H–J cHet mice were evaluated using the Barnes maze test. During the training phase, the cHet mice (n = 12 mice) failed to diminish the latency of the first entrance to the hiding box (H), indicating that Arid1a haploinsufficiency results in impaired spatial memory. Probe trials demonstrated that cHet mice also had spatial memory deficits (I), as they visited the target hole less often than the *Arid1a*$^{fl/+}$ mice (n = 12 mice) (J).

Data information: Data represent means ± SEM. In (B), $^{**}P < 0.01$ (cHet), $^{*}P < 0.05$ (cKO), unpaired two-tailed t-test. In (C), $^{*}P < 0.05$ (cHet), $^{***}P < 0.001$ (cKO), unpaired two-tailed t-test. In (E), $P > 0.9999$ (day 1), $P = 0.8500$ (day 2), $^{**}P < 0.01$ (day 3), $P = 0.0651$ (day 4), $^{*}P < 0.05$ (day 5), two-way ANOVA with Bonferroni *post hoc* test. In (F, G, I, J), $^{*}P < 0.05$, $^{**}P < 0.01$, $^{***}P < 0.001$, unpaired two-tailed t-test. In (H), $^{**}P < 0.01$ (Trial 5), two-way ANOVA with Bonferroni *post hoc* test.
Source data are available online for this figure.

and I), and had fewer target crossings than *Arid1a*$^{fl/+}$ mice in both the training phase and the probe test phase (Fig 1J). This indicates that the haploinsufficiency of *Arid1a* in excitatory neurons leads to deficits in learning and memory.

Arid1a cHet mice display impaired synaptic transmission and plasticity

To explore the potential mechanism of spatial learning and memory deficits in cHet mice, we investigated whether *Arid1a* haploinsufficiency affects hippocampal synaptic transmission and plasticity. We first performed whole-cell current-clamp recordings to investigate the effect of *Arid1a* deficiency on neuronal plasticity. We found that 91% of the recorded mature action potential (AP) showed a faster firing rate and consistent AP velocity, while the remaining 9% fired single- or no-spikes in the *Arid1a*$^{fl/+}$ group (Appendix Fig S3A and B). However, the proportion of single- or no-spike cells increased in *Arid1a* haploinsufficiency CA1 neurons, suggesting that partial loss of *Arid1a* impairs the maturity of hippocampal neurons (Appendix Fig S3B and C). Moreover, sodium and potassium currents (Appendix Fig S3D–H) exhibited downward trends in *Arid1a* haploinsufficiency CA1 neurons compared with the control cells.

To further study the function of *Arid1a* in hippocampal synaptic transmission, we measured the frequency and amplitude of spontaneous miniature excitatory postsynaptic currents (mEPSCs) in hippocampal CA1 neurons. In *Arid1a* haploinsufficiency CA1 neurons, mEPSCs decreased in amplitude and frequency compared to the control group (Fig 2A and B). We also performed paired-pulse facilitation across interpulse intervals of 20, 40, 50, 100, 200, 300, and 500 ms. Compared to *Arid1a*$^{fl/+}$ mice, we found a prominent reduction in the paired-pulse ratio in *Arid1a* haploinsufficient mice (Fig 2C and D). Finally, as long-term potentiation (LTP) is regarded as a cellular model for studying learning and memory, we tested whether *Arid1a* modulates long-term synaptic plasticity by examining LTP in *Arid1a*$^{fl/+}$ and cHet mice acute hippocampal slices. Compared to *Arid1a*$^{fl/+}$ mice, LTP was weakened in cHet mice (Fig 2E), and the amplitude of LTP 55–60 min after induction was lower in cHet mice (Fig 2F).

To further examine whether *Arid1a* is involved in hippocampal synaptic transmission, we analyzed the abundance of proteins involved in LTP in the whole hippocampal tissue postsynaptic density (PSD). Western blot assay demonstrated that haploinsufficient loss of *Arid1a* reduced the expressions of GRIA1, CaMKII, and PSD-95 in the hippocampi of cHet mice (Fig 2G and H). Together, these data indicate that *Arid1a* is required to maintain synaptic plasticity.

Haploinsufficiency of *Arid1a* in excitatory neurons shows reduced dendritic complexity and synapse density *in vivo*

Synaptic transmission and plasticity depend on neuronal morphology and spines (Goebbels *et al*, 2006; Cheng *et al*, 2018). To determine whether *Arid1a* haploinsufficiency affects neuronal morphology, we performed Golgi staining and analyzed dendritic complexity in the CA1 region of the hippocampi in *Arid1a*$^{fl/+}$and cHet mice. The number of basal and apical dendrites with ends, nodes, and intersections significantly decreased in cHet mice compared to the *Arid1a*$^{fl/+}$ mice, indicating that partial loss of *Arid1a* results in dendritic complexity deficits (Fig 3A–F). Moreover, compared with *Arid1a*$^{fl/+}$ neurons, cHet neurons had decreased spine densities in the hippocampi (Fig 3G and H). These results suggest that ARID1A plays a key role in maintaining synapse density and dendritic complexity.

Acetate supplementation ameliorates the cognitive deficits associated with *Arid1a* haploinsufficiency

Arid1a, as a chromatin remodeling factor, has been reported to be correlated with H3K27ac (Mathur *et al*, 2017). We first examined H3K27ac levels by Western blot and immunofluorescent staining and found that it was significantly decreased in excitatory neurons of forebrain tissues in cHet mice compared to their *Arid1a*$^{fl/+}$ siblings (Fig 4A and D). Previous studies have demonstrated that acetate supplementation can lessen the chronic MK-801-induced cognitive behavior phenotypes by monitoring the acetylation status of specific lysine residues of histones H3 and H4 (Singh *et al*, 2016), and the addition of acetate could block H3K9/27 acetylation loss in hESC (Moussaieff *et al*, 2015). Given the significant decrease of H3K27ac levels upon *Arid1a* haploinsufficiency, we reasoned that restoring H3K27ac activity by reagents could potentially be used to remodel the chromatin or gene transcription and could ameliorate impaired behaviors related to *Arid1a* haploinsufficiency.

We then investigated whether acetate supplementation could block H3K27 acetylation loss and restore phenotype deficits caused by *Arid1a* deletion. Intraperitoneal (i.p.) injection of acetate (100–1,000 mg/kg) did not significantly affect the body weight of the animals (Fig EV2A). Combined with the previous reference, we

Figure 2. Deficient basal transmission and plasticity in hippocampal synapses with *Arid1a* haploinsufficiency.

A Representative traces of spontaneous miniature excitatory postsynaptic currents (mEPSCs) in CA1 hippocampal acute slices from *Arid1a*$^{fl/+}$ and cHet mice.
B Quantification of amplitude (B, left) and frequency (B, right) spontaneous miniature excitatory postsynaptic currents (mEPSCs) in *Arid1a*$^{fl/+}$ and cHet hippocampal CA1 neurons. *Arid1a*$^{fl/+}$ (17 neurons from $n = 3$ mice) and cHet (15 neurons from $n = 4$ mice).
C Representative recording of the paired-pulse ratio at the 50-ms interpulse interval ms from slices prepared from *Arid1a*$^{fl/+}$ ($n = 4$) and cHet ($n = 4$) mice.
D Paired-pulse facilitation (PPF) studies across different interpulse intervals (20 ms, 40 ms, 50 ms, 100 ms, 200 ms, 300 ms, and 500 ms) revealed a significant difference in paired-pulse ratios at 50-ms intervals.
E Summary plots showing the time course of long-term potentiation (LTP) induced by frequency stimulation in the CA1 region from *Arid1a*$^{fl/+}$ or cHet mice. Field excitatory postsynaptic potential (fEPSP) traces before (black) and after (black or red) are shown in the inset above.
F Average amplitude of LTP measured at 55–60 min postinduction ($n = 8$ slices from 4 mice per group; 10 fEPSP slope (%) values were collected from 1 slice).
G Representative Western blot images for the expression of several LTP-related proteins in the *Arid1a*$^{fl/+}$ and cHet hippocampi.
H Quantification of the expression of each protein normalized to the β-actin level. Data information: Data represent means ± SEM. In (B, D, F, H), $^*P < 0.05$, $^{**}P < 0.01$, unpaired two-tailed *t*-test.

Source data are available online for this figure.

 ©2022 The Authors

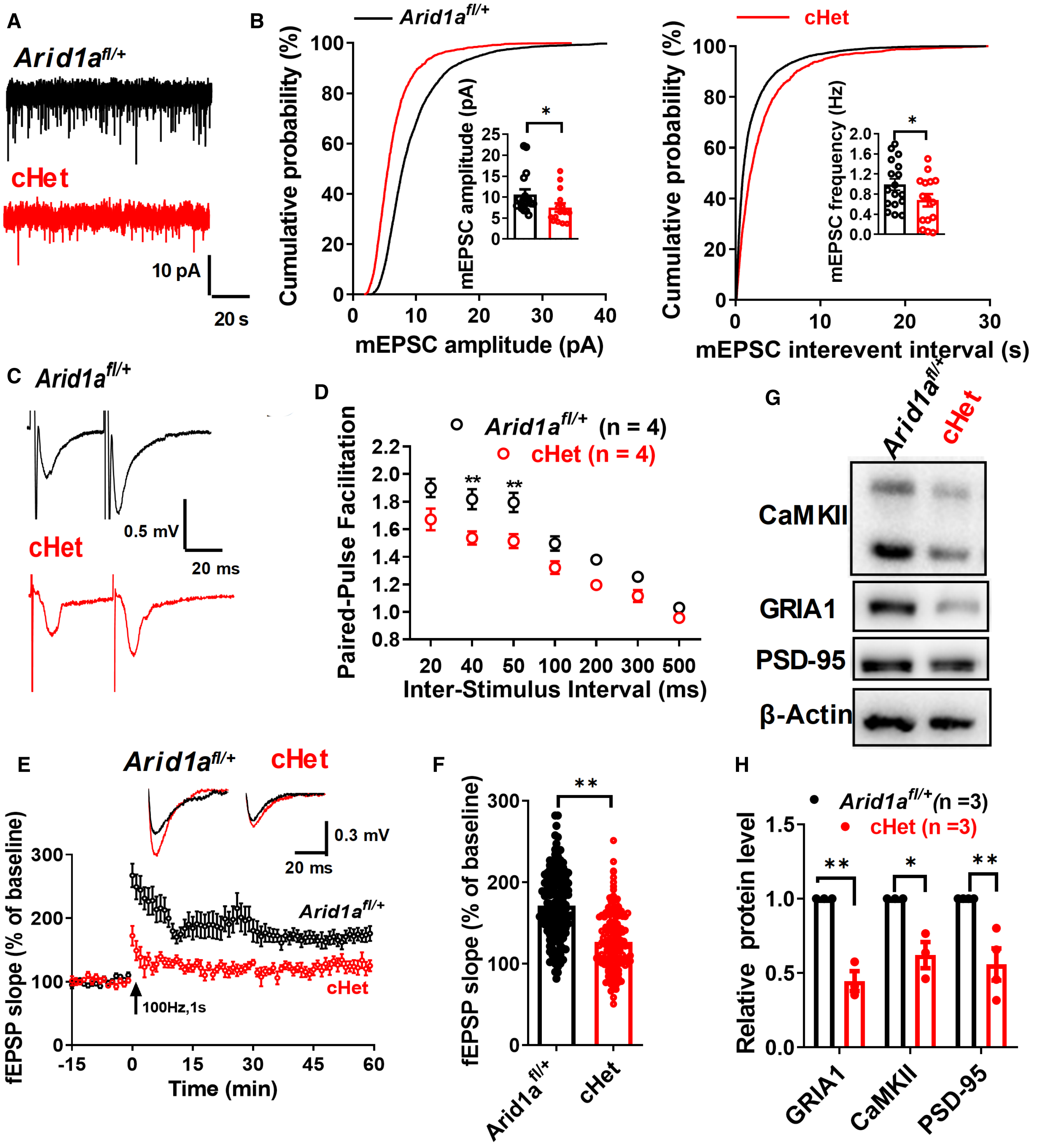

Figure 2.

selected two doses and found that a single daily i.p. injection of acetate for 28 consecutive days significantly increased the acetylation of H3K27 in forebrain tissues (Fig 4B, C, and E, and Fig EV2B). In the open field test, *Arid1a*$^{fl/+}$ and cHet mice treated with vehicle or acetate displayed similar locomotion (Fig EV2C and D). Importantly, acetate supplementation in *Arid1a* cHet mice could rescue their impaired spatial learning and memory in both the Morris water maze (Fig 4F–H) and the Barnes maze tests (Fig 4I–K).

We then tested whether acetate supplementation could rescue impaired synaptic transmission and plasticity. The results demonstrated that acetate supplementation restored impaired LTP (Fig 5A and B) and increased the paired-pulse ratio at an

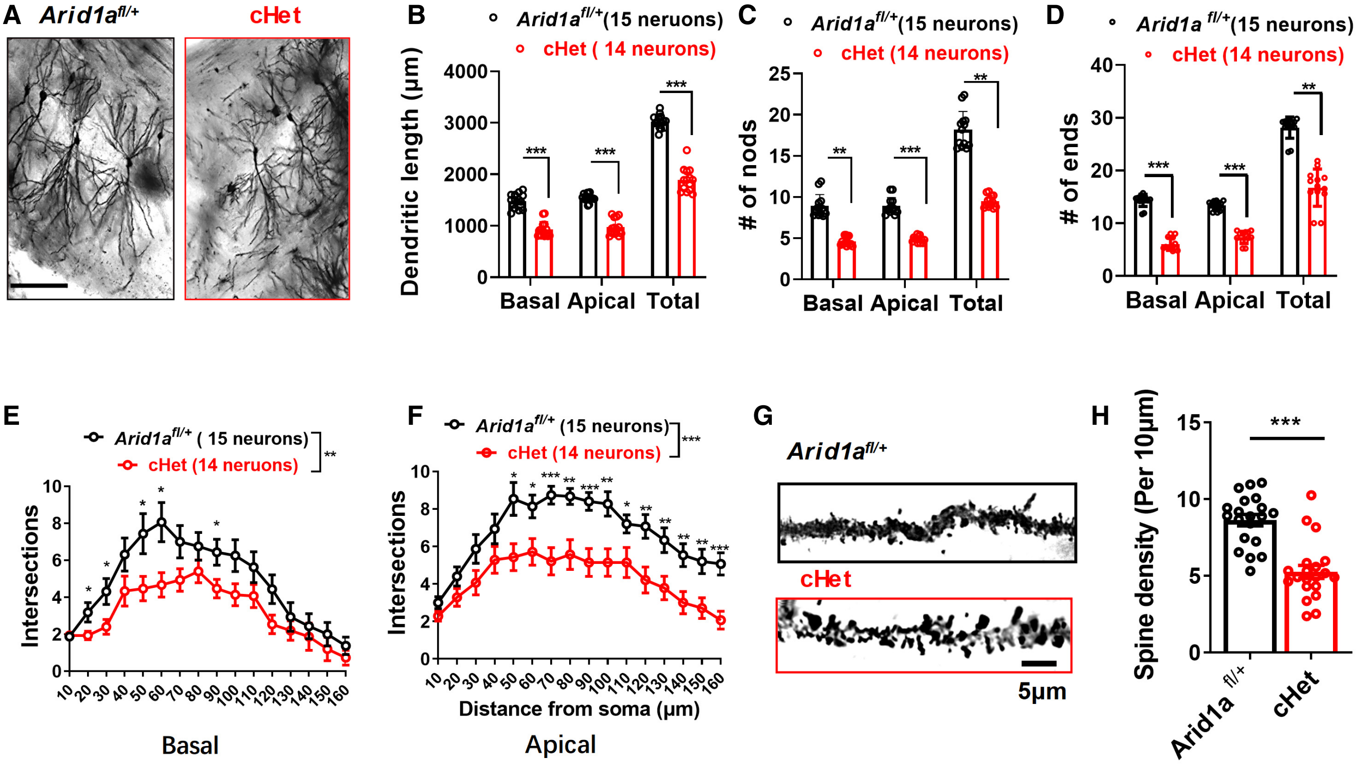

Figure 3. Haploinsufficiency of *Arid1a* in the excitatory neurons leads to impaired dendritic growth and synaptic loss *in vivo*.

A Golgi-stained coronal section in the hippocampal CA1 region of 2-month-old *Arid1a*$^{fl/+}$ and cHet mice. Scale bar, 100 μm.

B–D Partial loss of *Arid1a* significantly decreased dendritic length (B), dendritic nodes (C), and dendritic ends (D) in cHet neurons.

E, F Sholl analysis showing that CA1 pyramidal neurons of cHet mice exhibited decreased basal (E) and apical (F) dendritic complexity compared with neurons of *Arid1a*$^{fl/+}$ mice. $n = 6$ mice.

G Representative images of apical dendrites of CA1 pyramidal neurons are shown in (A). Scale bar, 5 μm.

H Spine density per 10 μm was measured on secondary dendrites (at least 20 neurons from 4 mice for each group).

Data information: Data represent means ± SEM. In (B–F), *$P < 0.05$, **$P < 0.01$, ***$P < 0.001$, two-way ANOVA with Bonferroni *post hoc* test. In (H), ***$P < 0.001$, unpaired two-tailed *t*-test.

interpulse interval of 50 ms in cHet mice (Fig 5C and D). Finally, we tested whether neuronal morphology deficits could be reversed after acetate treatment. Our data demonstrated that acetate increased spine density (Fig 5E and F) and dendritic complexity in *Arid1a* cHet mice (Fig EV2E–H). Altogether, these findings suggest that acetate supplementation ameliorates the

Figure 4. Supplementation with acetate rescues cognitive deficits caused by *Arid1a* haploinsufficiency in excitatory neurons.

A Left, Western blot analysis of H3K27ac in forebrain tissues of *Arid1a*$^{fl/+}$ and cHet mice. Right, relative quantitation of the intensity of H3K27ac normalized to the H3 level ($n = 3$ mice per group).

B Left, Western blot analysis of H3K27ac in forebrain tissues of vehicle- or acetate-treated cHet mice. Right, relative quantitation of the intensity of H3K27ac which was normalized to the H3 level ($n = 4$ mice per group).

C Experimental design showing the timeline for sodium acetate injection. cHet mice were treated with vehicle or acetate every day for 4 weeks.

D Immunofluorescence (IF) staining of H3K27ac(green) and Vgult1(red) in forebrain tissues of *Arid1a*$^{fl/+}$ and cHet mice, respectively. IF staining was performed on 40-μm thick floating sections. Relative fluorescence intensities of H3K27ac decreased upon the haploinsufficiency of *Arid1a* in the cortex. Scale Bar, 20 μm. *Arid1a*$^{fl/+}$ ($n = 7$) and cHet ($n = 4$) mice.

E Immunofluorescence staining of H3K27ac (green) and Vgult1 (red) in cortex tissues of vehicle- or acetate-treated cHet mice, respectively. IF staining was performed on 40-μm thick floating sections. Relative fluorescence intensities of H3K27ac increased after treatment with acetate in the cortex of cHet mice. Scale Bar, 20 μm ($n = 3$ mice per group).

F–H Morris water maze test of *Arid1a*$^{fl/+}$ and cHet mice treated with vehicle or acetate. Acquisition task, the latency to find the platform was used to assess learning ability. Latency to the platform was measured during training trials (F). Latency to the platform was measured during probe test (G). (H) Retention task, the times in the target crossings were used to assess memory retention. The times in the target crossings were measured in probe tests, $n = 10$–15 mice per group.

I–K Barnes maze test of *Arid1a*$^{fl/+}$ and cHet mice treated with vehicle or acetate. (I) During the training phase, the time to find the target in *Arid1a*$^{fl/+}$ and cHet mice that were treated with vehicle or acetate for 28 days. (J) In probe test trials, the time of latency to the platform to find the target in *Arid1a*$^{fl/+}$ and cHet mice treated with vehicle or acetate. (K) In probe trials, the numbers of target crossings of *Arid1a*$^{fl/+}$ and cHet mice treated with vehicle or acetate ($n = 12$ mice).

Data information: Data are represented as mean ± SEM. In (A, B, D, E, G, H, J, K) *$P < 0.05$, **$P < 0.01$, ***$P < 0.001$, unpaired two-tailed *t*-test.
Source data are available online for this figure.

 ©2022 The Authors

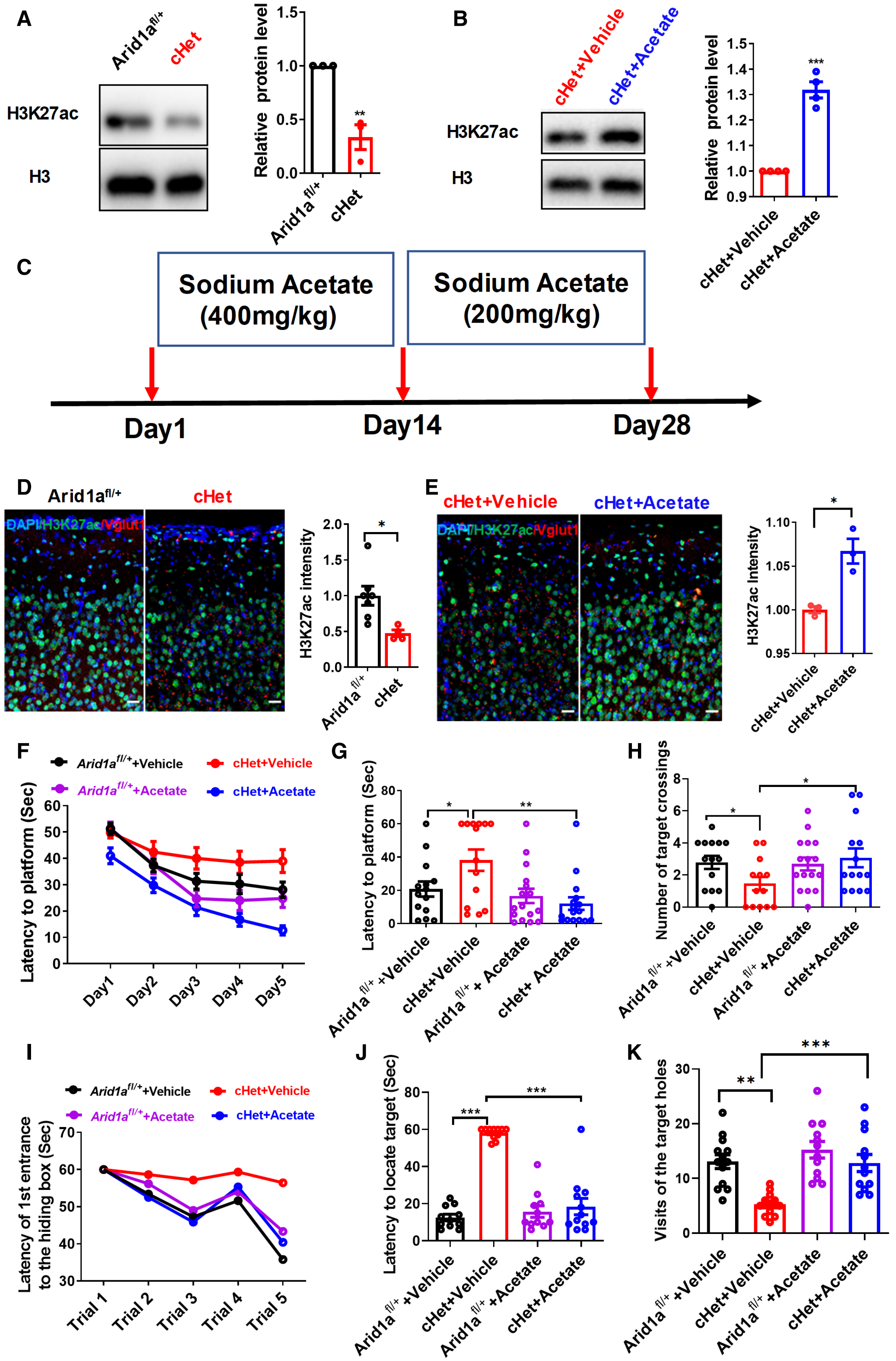
A
Arid1a fl/+
cHet
H3K27ac
H3
Relative protein level
Arid1a fl/+
cHet
B
cHet+Vehicle
cHet+Acetate
H3K27ac
H3
Relative protein level
C
Sodium Acetate (400mg/kg)
Sodium Acetate (200mg/kg)
Day1
Day14
Day28
D
Arid1a fl/+
cHet
DAPI/H3K27ac/Vglut1
H3K27ac intensity
E
cHet+Vehicle
cHet+Acetate
DAPI/H3K27ac/Vglut1
H3K27ac Intensity
F
Arid1a fl/+ +Vehicle
cHet+Vehicle
Arid1a fl/+ +Acetate
cHet+Acetate
Latency to platform (Sec)
Day1 Day2 Day3 Day4 Day5
G
Latency to platform (Sec)
H
Number of target crossings
I
Latency of 1st entrance to the hiding box (Sec)
Trial 1 Trial 2 Trial 3 Trial 4 Trial 5
J
Latency to locate target (Sec)
K
Visits of the target holes

Figure 4.

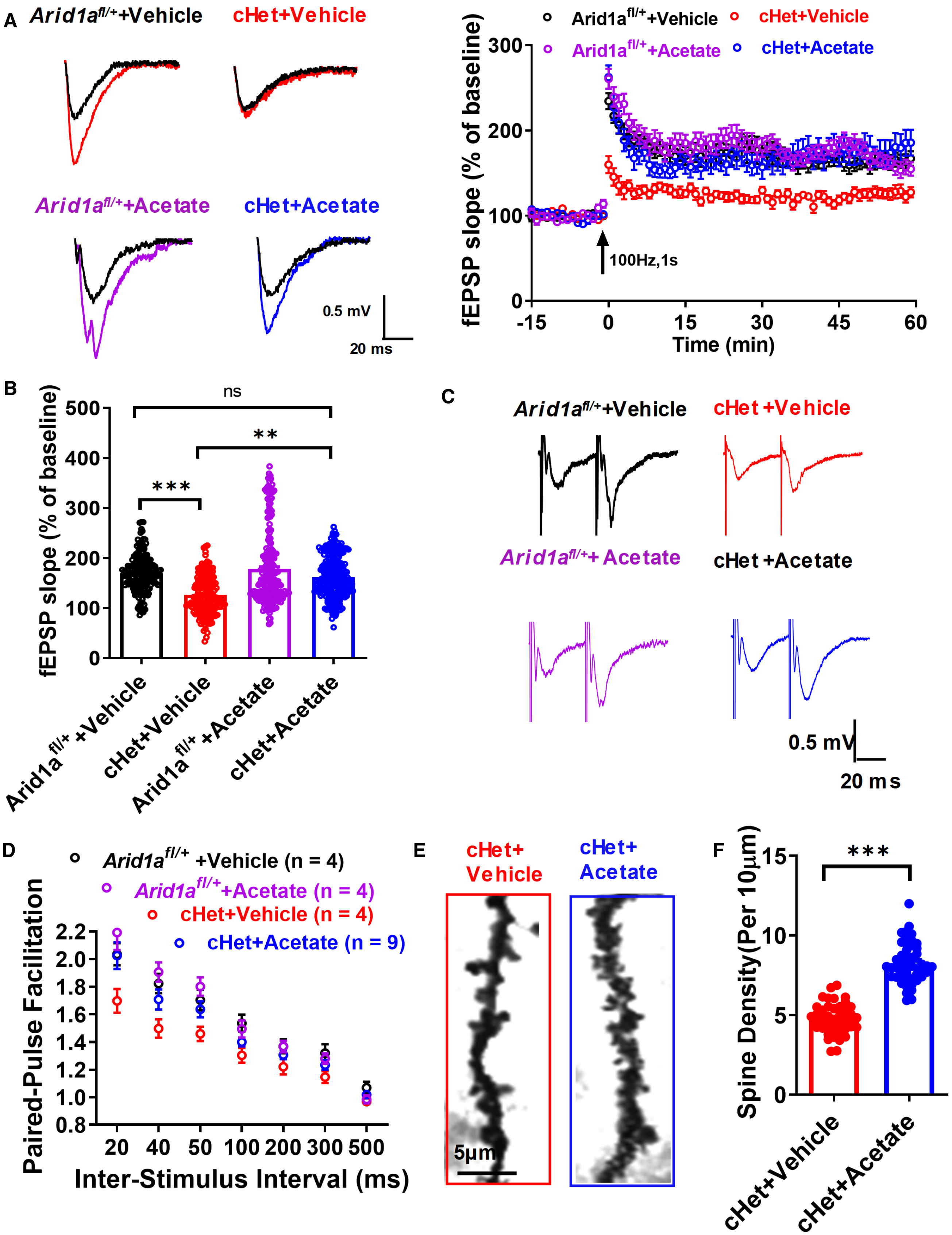
A
Arid1a^fl/+ +Vehicle
cHet+Vehicle
Arid1a^fl/+ +Acetate
cHet+Acetate
0.5 mV
20 ms
Arid1a^fl/+ +Vehicle
cHet+Vehicle
Arid1a^fl/+ +Acetate
cHet+Acetate
fEPSP slope (% of baseline)
100Hz,1s
Time (min)
B
fEPSP slope (% of baseline)
ns
**
***
Arid1a^fl/+ +Vehicle
cHet+Vehicle
Arid1a^fl/+ +Acetate
cHet+Acetate
C
Arid1a^fl/+ +Vehicle
cHet +Vehicle
Arid1a^fl/+ + Acetate
cHet +Acetate
0.5 mV
20 ms
D
Arid1a^fl/+ +Vehicle (n = 4)
Arid1a^fl/+ +Acetate (n = 4)
cHet+Vehicle (n = 4)
cHet+Acetate (n = 9)
Paired-Pulse Facilitation
Inter-Stimulus Interval (ms)
E
cHet+ Vehicle
cHet+ Acetate
5μm
F
***
Spine Density(Per 10μm)
cHet+Vehicle
cHet+Acetate

Figure 5.

Figure 5. Supplementation with acetate restores the deficient basal transmission and plasticity caused by *Arid1a* haploinsufficiency.

A, B *Arid1a*$^{fl/+}$ and cHet mice were injected with vehicle or acetate. Average traces for fEPSPs in hippocampal slices from each experimental group (A). (B) Average amplitude of LTP measured at 55–60 min postinduction (n = 8 slices from 4 mice per group; 10 fEPSP slope (%) values were collected from 1 slice).

C *Arid1a*$^{fl/+}$ and cHet mice were injected with vehicle or acetate. Representative traces of the paired-pulse ratio at an interval of 50 ms from slices prepared from each experimental group.

D Paired-pulse facilitation (PPF) was recorded with different interpulse intervals (20, 40, 50, 100, 200, 300, and 500 ms).

E Representative images of secondary branches of apical dendrites of vehicle- or acetate-treated cHet CA1 pyramidal neurons stained by the Golgi-Cox method (at least 20 neurons from 4 mice for each group). Scale bar, 5 μm.

F Spine density per 10 μm was measured on secondary dendrites in (E).

Data information: Data are represented as mean ± SEM. In (B, F), **$P < 0.01$, ***$P < 0.001$, ns = nonsignificant. Unpaired two-tailed t-test.

cognitive deficits associated with *Arid1a* haploinsufficiency in excitatory neurons.

ARID1A regulates H3K27ac deposition at neuronal genes

To reveal the molecular mechanisms underlying the impaired cognition and decreased neurite complexity in *Arid1a* haploinsufficiency mice, we performed transcriptome analysis of *Td*$^+$ excitatory neurons in the hippocampi of *Arid1a*$^{fl/+}$;*Nex-Cre*;*Td* mice at P28 (Fig EV3A). *Td*$^+$ excitatory neurons of littermate *Nex-Cre*;*Td* mice were set up as the control. The *Td*$^+$ cells were purified through fluorescence-activated cell sorting (FACS). Approximately 100,000 excitatory neurons were obtained from each mouse (2 mice from 2 littermates for each group). We then performed RNA sequencing (RNA-seq) of these *Td*$^+$ cells. To capture the overall similarity between the control samples and *Arid1a* haploinsufficiency samples, we performed a principal components analysis (PCA) on the transcriptomes. The PCA results demonstrated that the *Arid1a* cHet group and the control group were substantially different (PC1, 99.4928% variance), while there was almost no variability across the replicates in each group (PC2, 0.3590% variance; Fig EV3B). We found that a total of 3,815 genes were differentially expressed in the *Arid1a* haploinsufficiency samples compared with control samples ($P < 0.05$, fold change > 1.5 Fig EV3).

Gene ontology analysis demonstrated that genes downregulated upon partial loss of *Arid1a* were enriched in terms related to synapse and ion channels, such as synapse organization, divalent metal ion transport, and axon extension (Fig EV3C). To further identify enriched pathways perturbed by *Arid1a* haploinsufficiency, we subjected the differentially expressed genes (DEGs) to Kyoto Encyclopedia and Genes and Genomes (KEGG) analysis and found that the upregulated genes were more connected to inflammatory signatures such as tuberculosis, phagosome, synthesis, and degradation of ketone bodies (Fig EV3D). In contrast, genes downregulated by *Arid1a* haploinsufficiency are associated with neuronal functions such as genes encoding channels and proteins important for axon guidance, which is consistent with phenotypes of reduced neuronal firing and lack of electrical responses observed in *Arid1a* haploinsufficiency mice (Fig 6A).

To identify genes that are regulated by ARID1A, we examined H3K27ac by chromatin immunoprecipitation sequencing (ChIP-seq) analysis. PCA was based on enrichment signals at peak regions and indicated that the cHet group and the *Arid1a*$^{fl/+}$ group were different, while the two ChIP replicates in each group were highly reproducible (Fig EV3E). Among 6,960 H3K27ac peaks (Fig EV3F), GO analysis revealed that upregulated genes were associated with axon ensheathment and myelination, while the down-regulated categories were highly enriched for synaptic transmission, synapse organization, and ion transmembrane transport, similar to RNA-seq results (Fig EV3F). We then examined the overlap between down-regulated H3K27ac peaks with RNA-seq down-regulated genes following *Arid1a* haploinsufficiency and found 63 targets that show a reduction in both H3K27ac peaks and gene expression upon loss of Arid1*a* (Fig 6B). Gene ontology analysis of these 63 *Arid1a* target genes revealed significant enrichment for “synapse,” “membrane potential,” and “neurotransmitter levels,” and terms related to neuronal function, including *Gabra1*, *Gabrb2*, *Fzd1*, *Grin2a*, *Slitrk1*, and *Cacna2d1* (Fig 6C). We then validated the expression of a subset of ARID1A target genes using qRT-PCR and found that the mRNA levels of genes acting as the downstream targets of synaptic transmission (*Gabrb2*, *Gabra1*, *Slitrk1*, and *Cacna2d1*) were significantly downregulated in *Arid1a* haploinsufficiency neurons (Fig 6D), suggesting that *Arid1a* plays an essential role in synapse functions by modulating the deposition of the H3K27ac mark at key neuronal genes.

Next, we investigated the effect of acetate on the genome-wide occupancy of H3K27ac. ChIP-seq analysis showed that acetate

Figure 6. Identification targets of ARID1A in excitatory neurons.

A KEGG pathway analysis of downregulated targets in Arid1a-haploinsufficiency transcriptome.

B Venn diagram showing the overlap between down-regulated H3K27ac ChIP-seq peaks with downregulated genes following Arid1a-haploinsufficiency (RNA-seq).

C Gene ontology enrichment analysis of 63 genes with decreased H3K27ac enrichment and downregulated expression. Displayed terms are all with “*P-adjust* < 0.05.”

D qPCR analysis of 63 genes with known functions related to synaptic transmission in the *Arid1a* cHet mice (n = 4 mice per group).

E Effect of acetate supplementation on genome-wide occupancy of H3K27ac as determined by ChIP-seq, compared to the vehicle-treated group. ChIP-seq was performed using cHet forebrain treatment with acetate compared to cHet forebrain treatment with PBS. The unit of the color key is normalized RPKM.

F Genome views of H3K27ac ChIP-seq in forebrain tissues of vehicle or Acetate-treated cHet mice at the *Gabra1* and *Gabrb2* loci. Promoters are highlighted with vertical shaded boxes (yellow).

G qPCR analysis of the effect of acetate on the indicated genes in (C) in cHet mice (n = 4 mice per group).

Data information: Data are represented as mean ± SEM. In (D, G), *$P < 0.05$, **$P < 0.01$, ***$P < 0.001$, unpaired two-tailed t-test.

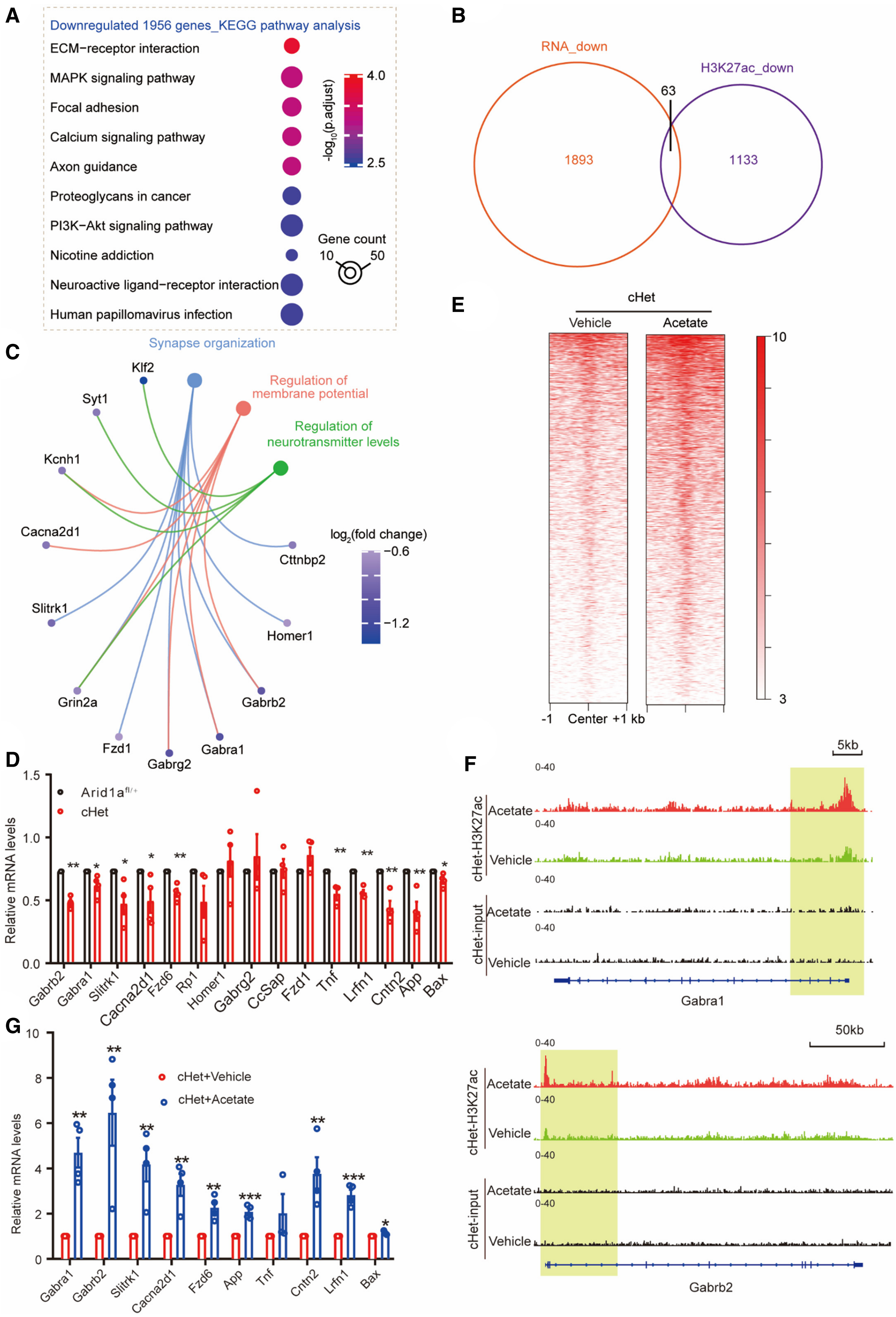

Figure 6.

©2022 The Authors

significantly slowed the reduction of H3K27ac peaks in Arid1a haploinsufficiency mice compared to *Arid1a*$^{fl/+}$ mice (Fig 6E and Fig EV3G). Moreover, the peak density of H3K27ac on the promoters of *Gabra1* and *Gabrb2* increased in *Arid1a* cHet mice treated with acetate (Fig 6F). RT-qPCR analysis demonstrated that acetate reversed the alteration of the transcription of genes involved in synaptic transmission in cHet mice upon acetate treatment (Fig 6G). This indicates that acetate could exert its effect on neuronal genes through ARID1A-dependent regulation of H3K27 acetylation.

Deletion of *ARID1A* results in disrupted neurite outgrowth and electrophysiological defects in hESC-derived neurons

Given that loss-of-murine *Arid1a* leads to severe defects in synaptic transmission, learning, and cognition in cHet mice, we then asked whether human *ARID1A* plays similar roles. To test this idea, we first generated two *ARID1A*-deletion clones (KO1, KO2) using CRISPR/Cas-9 technology, as well as an un-edited control clone from the same parental H9-ESC clone (Appendix Fig S4A). ARID1A proteins (Appendix Fig S4B and C) were almost undetectable in both KO1 and KO2 clones. To verify the pluripotency of *ARID1A* KO hESC clones, we investigated the expression of stemness and pluripotency markers (Oct4 and Nanog) and found indistinguishable expression levels of pluripotency markers in *ARID1A* WT and KO cell lines using immunofluorescence staining, qPCR, and Western blot analyses (Appendix Fig S4D–H).

Next, to examine the function of ARID1A during default neural differentiation from hESCs to hNSCs under defined conditions, we maintained the rosette-like cells at P14 as neural spheres in the neural progenitor cells (NPCs) medium. We found that *ARID1A* KO NPCs showed no significant difference in BrdU incorporation compared to control cells (Appendix Fig S4I–K), indicating that depletion of *ARID1A* does not delay the exit of pluripotency in hNPCs.

We next determined whether loss of *ARID1A* affects the morphology of hESC-derived neurons. By tracing MAP2-stained neurons (Fig EV4A), we found that there were substantial decreases in total dendritic length and the numbers of branches, ends, and nodes in *ARID1A* KO hESC-derived neurons compared to WT neurons, and that there was no difference in any morphological parameters between *ARID1A* KO1 and KO2 hESC-derived neurons on day 40 of neural differentiation (Fig EV4B–E). We then investigated whether *ARID1A* deletion affects spines in hESC-derived neurons and examined the presynaptic and postsynaptic proteins, synaptophysin (SYN) and PSD-95, respectively. In mature neurons, significantly reduced numbers of SYN and PSD-95 puncta were observed in *ARID1A* KO hESC-derived neurons on day 55 of neural differentiation using immunofluorescence staining with SYN and PSD-95 antibodies (Fig EV4F–H).

To further investigate whether *ARID1A* loss-of-function affects the maturity of hESC-derived neurons, we performed whole-cell patch recordings of hESC-derived neurons on day 55 of neural differentiation (Fig EV5A). We found that 72.7% of the cells fired repetitive spikes in the WT group. In contrast, the ratio of immature neurons and no-spike cells significantly increased in both *ARID1A* KO1 (62.5%) and KO2 (65.6%) groups, indicating that loss of *ARID1A* inhibits the maturation of hESC-derived neurons (Fig EV5B–D). Moreover, compared with the control cells, sodium and potassium currents (Fig EV5E–H) displayed downward trends in *ARID1A* KO hESC-derived neurons, suggesting that *ARID1A* KO hESC-derived neurons have impaired electrophysiological features.

Finally, to test whether *ARID1A* affects the maturity of physical synapses, we performed whole-cell patch-clamp recording to measure mEPSCs in cultures between days 55 and 70 (total $n = 48$ neurons). In contrast to WT neurons, *ARID1A* KO neurons showed a decrease in mEPSCs frequency and a significant decline in mEPSC amplitude (Fig EV5I–K), indicating a presynaptic and postsynaptic deficit. Together, these morphological and physiological data suggest that *ARID1A* ablation impairs synaptic transmission in hESC-derived excitatory neurons.

Acetate supplementation rescues neuronal dysmorphology caused by *ARID1A* deletion in hESC-derived neurons

Finally, we tested whether acetate supplementation affects the morphologic features of human hESC-derived neurons. We treated human *ARID1A* KO and WT neurons with acetate (10, 100 μM, 1 mM) and quantified their morphological features. Compared to vehicle-treated groups, we observed an enhancement in the dendritic length in acetate-treated *ARID1A* KO neurons in a dose-dependent manner (Fig 7A and B). Upon acetate treatment at a concentration of 100 μM, there was a trend toward more intersections at a distance of 40–90 μm from the soma, while there was no

Figure 7. Acetate supplementation rescues the decreased neurite complexity in hESCs-derived neurons caused by *ARID1A* deletion.

A Representative image of vehicle- and acetate-treated neurons derived from WT or *ARID1A* KO hESCs on day 40 of neural differentiation Scale Bar, 20 μm.

B Quantification of total neurite length of hESC-derived neurons after treatment with acetate or vehicle. n = at least 34 neurons for each group from 3 independent experiments.

C Sholl intersection profiles of neurons treated with vehicle or acetate on day 40 of neural differentiation (n = at least 34 neurons for each group from 3 independent experiments). n.s., nonsignificant.

D Representative images of dendrites (MAP2, green) showing localization of foci of the pre- and postsynaptic protein complexes, synaptophysin (red), and PSD-95 (white) protein in control, acetate treatment of WT and *ARID1A* KO hESC-derived neurons on day 55 of neural differentiation. Scale Bar, 5 μm.

E, F Quantification of the spine numbers from synaptophysin (E) and PSD-95 protein (F) stained secondary dendrites of neurons on day 55 of neural differentiation (Data are representative of three independent experiments).

G qRT-PCR analysis of genes related to synaptic transmission in the WT and *ARID1A* KO hESC-derived neurons on day 55 of neural differentiation (Data are representative of three independent experiments).

H qRT-PCR analysis of genes related to synaptic transmission in the WT and *ARID1A* KO hESC-derived neurons treated with acetate or vehicle on day 55 of neural differentiation. (Data are representative of three independent experiments).

Data information: Data are represented as mean ± SEM. In (B, E, F, G, H) $^*P < 0.05$, $^{**}P < 0.01$, $^{***}P < 0.001$, unpaired two-tailed t-test. In (C), $^*P < 0.05$, $^{**}P < 0.01$, $^{***}P < 0.001$; ANOVA.

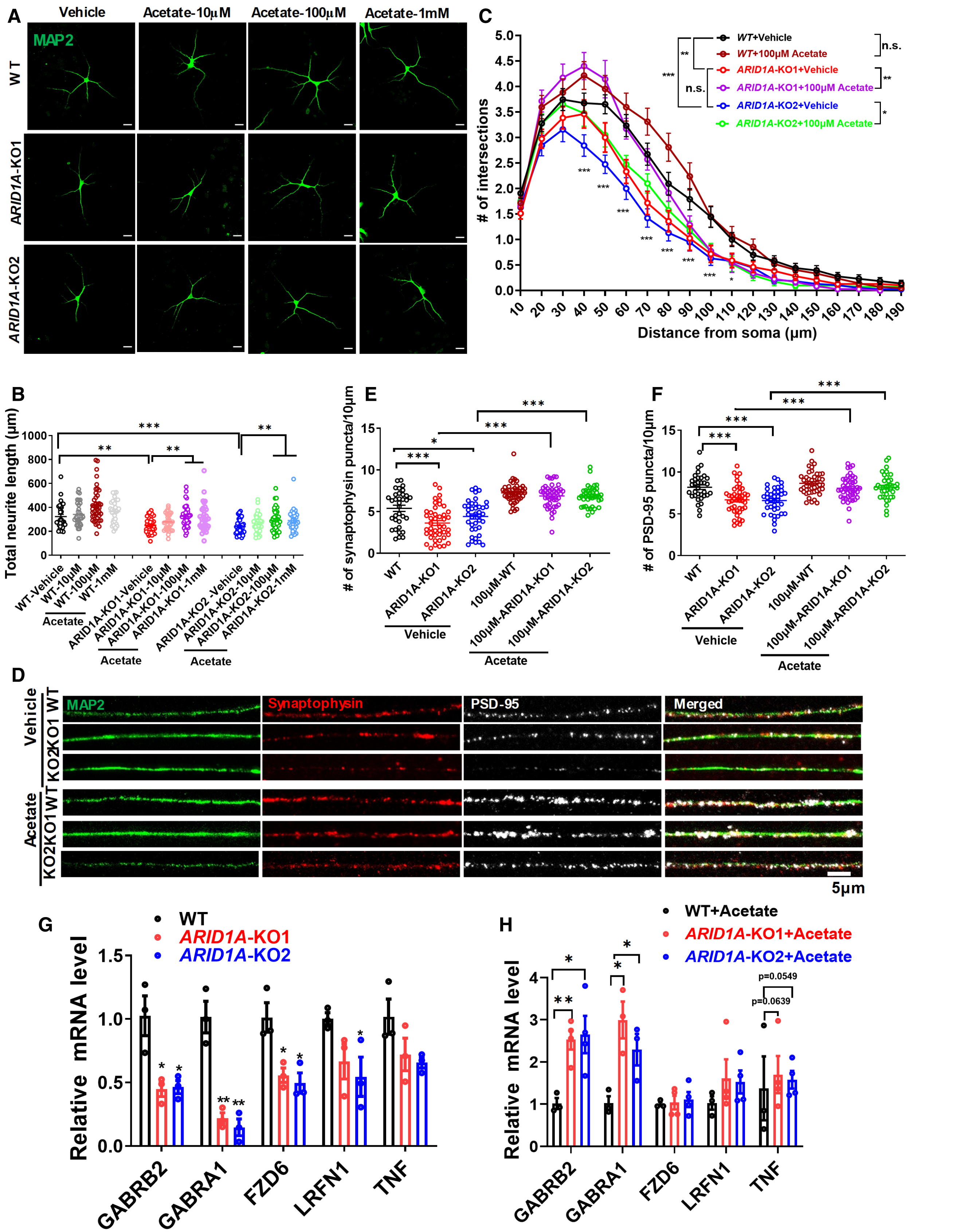

Figure 7.

©2022 The Authors

significant difference in the number of intersections between vehicle- and acetate-treated WT groups (Fig 7C). However, both *ARID1A* KO1 and KO2 hESC-derived neurons had more intersections upon acetate treatment compared with vehicle-treated neurons (Fig 7C). Importantly, treatment of *ARID1A* KO neurons with acetate (100 μM) induced a significant increase in both SYN and PSD-95 puncta numbers (Fig 7D–F). As such, these results strongly support that acetate could improve the morphological deficits in human *ARID1A* KO neurons.

Next, we examined the effects of acetate on the expression of ARID1A targets. We found that important neuronal genes related to neural morphology and cognition regulation, such as *GABRA1*, *GABRB2*, *FZD6*, *LRFN1*, and *TNF*, decreased in *ARID1A*-null differentiated neurons (Fig 7G). Moreover, the expression levels of these genes (*GABRA1*, *GABRB2*, and *TNF*) were significantly elevated in *ARID1A*-null differentiated neurons upon acetate treatment (Fig 7H). These results indicate that acetate has therapeutic potential for neuronal dysfunction in ID with *ARID1A* loss-of-function.

Discussion

ARID1A haploinsufficiency has been proposed to cause CSS and intellectual disability (Tsurusaki *et al*, 2012; Kosho *et al*, 2013; Santen *et al*, 2013), but the neurobiological basis for ARID1A-haploinsufficiency-related neurodevelopmental disorders remains unknown. In this study, we generated both mouse models and hESC-derived neurons to explore the role and mechanism of ARID1A in excitatory neurons. We demonstrated that haploinsufficiency of *Arid1a* disrupts the development and function of excitatory neurons and that behavioral phenotypes in mice result in cognitive deficits observed in individuals with intellectual disabilities. Our mechanistic studies suggest that ARID1A haploinsufficiency in excitatory neurons disrupts histone modifications at genes essential for the function of excitatory neurons and cognition (Appendix Fig S5). We also demonstrated that acetate supplementation restores cognitive deficits and abnormal neuronal morphology in both murine and human neurons.

To test whether *Arid1a* is related to intellectual disabilities, we established three mouse models to knockdown or delete *Arid1a* levels and found that excitatory neurons were the central underlying cause of CSS. Our data demonstrate that *Arid1a* haploinsufficiency in excitatory neurons results in behavioral abnormalities and impairs the transcription program related to learning and memory. These results indicate that our mouse model successfully reflects the behavioral phenotypes of intellectual disability and CSS (Bogershausen & Wollnik, 2018). Notably, *Arid1a* haploinsufficiency neurons displayed impairments in synaptic transmission, synaptic plasticity, and abnormal pattern of PSD gene expression. PSD-95 acts as a scaffolding protein with a docking site for synaptic regulatory proteins clustering around NMDA-and AMPA-type glutamate receptors, which are essential for excitatory neurotransmission and synaptic plasticity (Sheng, 2001). Synaptic dysfunction could be linked to compromised presynaptic neurotransmitter release and the progressive loss of synapses from dendrites, which could alter synaptic plasticity and functional responses (Rotterman *et al*, 2014). These data suggest that our *Arid1a* haploinsufficiency model can be used to further investigate intelligence and memory behavior, which could define how cognitive function evolves from mice to humans. Additionally, to further confirm the results obtained from the mouse model, we established a human *ARID1A* KO hESCs model. When using *ARID1A* KO hESCs upon differentiation to human excitatory neurons, we observed simple neuron morphology, decreased mEPSC frequency and amplitude, and fewer synapses, suggesting lessened neuronal differentiation/maturation. Therefore, our data in human neurons are consistent with the essential role of *Arid1a* for murine hippocampus and to determine the fate of excitatory neurons, which provides suitable models for investigating the roles of ARID1A, its underlying mechanisms, and screening potential therapeutic intervention for CSS.

Acetylation of histones can impact chromatin accessibility and alter gene expression (Zhou *et al*, 2009). Our immunoblot data demonstrated that H3K27ac levels declined in cHet mice and then combined with VGLUT1 staining, which is found on the surface membrane of almost all excitatory neurons in the cortex (Li *et al*, 2020). This indicates that the decrease in H3K27ac was primarily due to excitatory neurons in *Arid1a* haploinsufficiency cHet mice. Our results suggest a potential role of ARID1A in remodeling the chromatin complex by inducing the physical access of histone-modifying enzymes such as p300 and HDAC to histone molecules (Cramer *et al*, 2017; Wilson *et al*, 2020). Therefore, the target genes in the condensed chromatin can be altered into a more relaxed structure to generate favorable environments for greater levels of gene transcription. The BAF complex is required for histone acetylation catalyzed by acetyltransferases (Naidu *et al*, 2009; Wilson *et al*, 2020). In aging mice, brain memory disturbances are related to altered histone acetylation during learning and the failure to recruit a hippocampal gene expression program associated with memory consolidation (Peleg *et al*, 2010). Inhibitors of histone deacetylase (HDAC), which act to upregulate histone acetylation levels, can improve cognitive performance in Alzheimer's disease models (Graff *et al*, 2012). Our data demonstrate that *Arid1a* haploinsufficiency reduces the expression levels of Gamma-Aminobutyric Acid (GABA) type A receptors, such as *Gabra1* and *Gabrb2*, and adjusts epigenetic signatures in the brain. Decreases in histone acetylation at the *Gabra1* promoter could initiate signaling cascades involved in restricting *Gabra1* expression and function (Bohnsack *et al*, 2017). *Gabra1* is a major contributor to inhibitory synaptic currents, but inhibitory plasticity can also affect activity-dependent excitatory plasticity (Wang & Maffei, 2014; Lin *et al*, 2015). Subsequently, synaptic transmission is significantly disrupted, resulting in a variety of Alzheimer's disease-like cognitive deficits (Zheng *et al*, 2019). More importantly, we found that the addition of acetate, which increases H3K27 acetylation, leads to the recovery of GABA type A receptor and synaptic function and improves cognitive impairment in *Arid1a* haploinsufficiency mice and hESC *ARID1A* null neurons. This is consistent with findings that acetate enhancement is related to GABA synthesis in the cerebellum (Jin *et al*, 2021). Our results provide strong evidence that acetate supplementation underlying GABA signaling is a promising therapeutic intervention for CSS patients. While the neurotransmitter GABA prevents the activity of signal-receiving neurons by networking with the GABA type A receptor, a better understanding of excitatory neuron-specificity regulatory mechanisms is needed, such as combining with excitatory specific reporter systems to distinguish excitatory and other type neurons.

©2022 The Authors

We found that specific cognitive and neural circuitry impairment in the *Arid1a* haploinsufficiency brain can be overcome by acetate interventions, presumably by targeting histone deacetylation. Similar treatment in other models of monogenic brain disorders has provided mixed results (Tillotson *et al*, 2017; Rotaru *et al*, 2018; Mukai *et al*, 2019). Our findings indicate that the addition of acetate in *Arid1a* haploinsufficiency mice fully reverses behavioral abnormalities and synaptic plastic deficits. Moreover, acetate supplements counteract the downstream effects of *ARID1A* deficiency in hESC-derived neurons. However, our results demonstrate that acetate supplementation could rescue several targets (*GABRA1*, *GABRB2*, and *TNF*) observed in *Arid1a* haploinsufficiency mice, suggesting a big difference between human and murine systems. In this study, we generated hESC clones of *ARID1A*-deletion from the H9 line. Using more *ARID1A*-edited clones from other hESC lines (e.g., H1) would maximize statistical power and inference capability. Considering that the correlation between RNA transcript numbers and protein products is quite low in the brain (Schwanhausser *et al*, 2011), proteomic profiles and functional data from human cerebral organoids are also required before translating CSS models to human patients.

In conclusion, our study demonstrates that *Arid1a* regulates synaptic plasticity and hippocampal-dependent cognitive function. We found that H3K27 acetylation plays a role in regulating neuronal-dependent gene expression and reveals the essential role of histone modifications in maintaining basal patterns of cell-type-specific gene expression in the brain, which is required to support synaptic connectivity and behavioral plasticity. Collectively, our results support an essential role for ARID1A associated with H3K27ac as a neuromodulator in the forebrain to regulate synaptic plasticity, learning and memory, and cognitive function.

Materials and Methods

Animals

All mice were from a C57BL6 genetic background. The $Arid1a^{fl/fl}$ mice (a gift from Dr. Zhong Wang at the University of Michigan) possessed loxP sites flanking exon 8 of the *Arid1a* gene (Gao *et al*, 2008). The *Emx1-Cre* (Strain #:005628, C57BL/6 background) was bought from the Jackson lab. The *Nex-Cre* mice (C57BL/6 background) were provided by the lab of Feng-Quan Zhou, Johns Hopkins University (Goebbels *et al*, 2006). The $Arid1a^{fl/fl}$ mice were crossed with a mouse line (*Nex-Cre*) to generate $Arid1a^{fl/+}$; *Nex-Cre* (hereafter referred to as cHet) mice. $Arid1a^{fl/+}$;*Nex-Cre* mice were further crossed with $Arid1a^{fl/fl}$ mice to obtain homozygous $Arid1a^{fl/fl}$; *Nex-Cre* (hereafter referred to as cKO) mice in which *Cre*, under the control of the NEX promoter, drives the deletion of *Arid1a* specifically in early postmitotic, excitatory neurons of the developing forebrain. The *Emx1-Cre* and $Arid1a^{fl/fl}$ mice were crossed to generate $Arid1a^{fl/+}$;*Emx1-Cre* mice. $Arid1a^{fl/+}$; *Emx1-Cre* mice were further crossed with $Arid1a^{fl/fl}$ mice to obtain homozygous $Arid1a^{fl/fl}$;*Emx1-Cre* mice. Mice genotypes were performed by PCR assay using tail genomic DNA. Genotyping primers are listed in Appendix Table S1 (Appendix). Mice were housed in specific pathogen-free (SPF)-like conditions, with a maximum of five mice per cage. All mice were maintained in a room at a constant temperature (23°C) with a regular 12-h light/dark cycle with ad libitum access to food and water. All animal procedures were approved by the Animal Committee of the Institute of Zoology, Chinese Academy of Sciences, and were conducted in accordance with the guidelines of national ethical regulations for animal care and use in research (IOZ20180025).

Stereotactic injection

Adeno-associated virus (AAV) 2/9 virus was produced as previously described (Hordeaux *et al*, 2019; Bravo-Hernandez *et al*, 2020). In brief, HEK293 cells were transfected with the transfer plasmid pAAV carrying EGFP (pAAV-CMV-EGFP-pA, BrainVTA) or CRE recombinase (rAAV-CMV-GFP-P2A-CRE-WPRE-bGH-pA, BrainVTA), the helper plasmid carrying adenovirus-derived genes (AD helper, Addgene plasmid #112867), and the Rep/Cap plasmid carrying AAV2 replication and AAV9 capsid genes (pAAV2/9n, Addgene plasmid #112865), which together supply all of the trans-acting factors required for AAV replication and packaging. Recombinant AAV viral particles were harvested 72 h posttransfection and purified by cesium chloride (CsCl) density gradient ultracentrifugation. Purified AAV titers were determined by real-time quantitative PCR using primers targeted at the ITR (forward primer, 5′-GGAACCCCT AGTGATGGAGTT; reverse primer, 5′-CGGCCTCAGTGAGCGA). In the intracranial injection experiments, 1 μl of AAV virus (packaged by BrainVTA; Titer: > 6.15×10^{12} V.G./ml) was injected stereotaxically into each hippocampal region at 200 nL/min, and a total of 2 μl virus for each 6-week-old male $Arid1a^{fl/fl}$ mouse (stereotaxic coordinates from Bregma: 2.0 mm caudal, 1.2 mm lateral, 2.0 mm ventral; 2.8 mm caudal, 2.0 mm lateral, 1.7 mm ventral).

Behavioral assays

Two- to three-month-old male mice were used for behavioral assays. Mice were moved to the testing room for acclimation 24 h before behavioral testing. Animals injected with the AAV virus were subjected to immunostaining analysis to verify the viral infection, and animals without viral infections were excluded from the analysis. All tests were conducted blind.

Open field test

The open field arena was made of a piece of plywood (painted white) that was 72 cm long × 72 cm wide × 36 cm tall. A central zone (18 × 18 cm) was drawn in the middle of the open field arena. Each mouse was placed in one corner of the open field arena and allowed to freely explore the apparatus for 30 min. This test used a video camera to record their movements in the peripheral and central squares.

Morris water maze test

A circular water tank (120 cm in diameter) was filled with water, which was made opaque with nontoxic white paint. At the center of a given quadrant of the water tank, a round platform (13 cm in diameter) was hidden 1 cm under the surface of the water. Mice were trained for 4 successive days in the Morris water maze, and each session consisted of 4 trials. For each trial, the mouse was

 ©2022 The Authors

released from the tank wall and placed on the platform for 20 s during the 1960s. Probe tests were conducted 24 h after the last training. During the probe test, the platform was removed and the task performances were recorded. A video camera was used to record the swimming tracks of a mouse in the water pool and the swimming speed, and time spent in each quadrant.

Barnes maze task

The Barnes maze test for mice consists of three phases: 1 day for habituation, 3 days for training, and 1 day for the probe. On Day 1, during which white noise was played, the mice were placed in the center of the apparatus beneath a clear 3,500-ml glass beaker for 30 s. Mice were slowly introduced to the target hole for 15 s by moving the glass beaker. Then, the mice were allowed to independently enter through the target hole into the hiding box for 1 min. For the training phase, mice were put into an opaque cubic box in the center of the apparatus for 15 s. After 15 s, white noise was played, the cubic box was lifted, and the mouse was allowed to explore the maze for 1 min. 72 h after the final training, the probe trial was executed similarly to the training trials, except that the hiding box was removed. For the probe trial, time spent per quadrant and HS per quadrant was recorded. For data analysis, the maze was divided into equal quadrants containing 5 holes and in the center of the target quadrant was the target hole.

Drug treatment in the animals

Sodium acetate was purchased from Sigma-Aldrich (Cat# A13184). Mice received 200 mg/kg (dissolved in 1× PBS) through intraperitoneal injection or vehicle (1x PBS), vehicle: 1x PBS (200 μ/per mouse). Behavioral testing occurred one month after treatment.

Electrophysiology

Hippocampal slice preparation and recording Hippocampal field excitatory postsynaptic potentials (fEPSPs) were recorded using mice from 8 to 12 weeks old. Experiments were performed at room temperature of 22–25°C. The preparation of hippocampal slices was performed as previously described (Cheng *et al*, 2018). Briefly, the brain was removed into an ice-cold artificial cerebrospinal fluid (aCSF) bath including the following: 124 mM NaCl, 1.5 mM MgCl2, 3.3 mM KCl, 1.3 mM NaH2PO4, 26 mM NaHCO3, 11 mM Glucose, and 2.5 mM CaCl2, saturated with 95% O_2/5% CO_2, and prepared transverse hippocampal slices (400 μm) using vibratome slicer (Campden instruments, 7000smz). These slices were kept continuously oxygenated and recordings began at least 1 h later. fEPSPs were recorded from the stratum radiatum of CA1 using a glass pipette (2–3 MΩ) filled with 2 M NaCl. Stimulation pulses (0.033 Hz) were delivered to Schaffer collateral using a bipolar concentric stimulating electrode (FHC Inc., Bowdoin, ME). Following stable baseline recording for at least 15 min, LTP was induced by 100 Hz-tetanic stimulation (1 train of 100 Hz for 1 s) at a baseline stimulation intensity. For PPF recordings, the stimulus intensity was adjusted to evoke an fEPSP that was 2/3 of the maximum. PPF was assessed using interstimulus intervals of 20, 40, 50, 100, 200, 300, and 500 ms. Data were collected and analyzed using pClamp 10.6 and Clampfit 10.6 (Axon Instruments, CA, USA), respectively.

Whole-cell voltage-clamp or current-clamp recording was performed with borosilicate glass micropipettes (resistance 6–10 MΩ), which were filled with internal solutions containing 135 mM of potassium gluconate, 7 mM of NaCl, 2 mM of MgATP, 10 mM of HEPES, 0.3 mM of Na2GTP, and 2 mM of MgCl2, adjusted to pH 7.4 with KOH. The extracellular fluid contained: 124 mM NaCl, 3.3 mM KCl, 1.5 mM MgCl2, 1.3 mM NaH2PO4, 26 mM NaHCO3, 11 mM glucose, and 2.5 mM CaCl2 (pH 7.4). For miniature excitatory postsynaptic current (mEPSC) recordings, cells were voltage-clamped at −60 mV. Recording pipettes were filled with a solution containing (in mM): 115 cesium methane sulfonate, 15 CsCl, 2 MgCl2, 10 EGTA, 10 HEPES, 4 ATP (Mg), and 1 QX-314, osmolarity adjusted to 290 mOsm, pH adjusted to 7.2 by CsOH. Bicuculline (10 μM) and tetrodotoxin (TTX; 1 mM) were added to the bath solution to isolate AMPA receptor-mediated mEPSCs. Whole patch-pipette micromanipulation was obtained by an infrared differential interference contrast video-microscopy (Nikon, Eclipse fn1, Japan). Under current-clamp mode, intracellular membrane electrical potentials were recorded using a Multi-clamp 700B amplifier (Molecular Devices, Palo Alto, CA, USA) and an AxonTM Digidata 1440A digitizer (Molecular Devices). For voltage-clamp recordings, cells were held at −60 mV. Data were digitized at 10 kHz with a 2 kHz low-pass filter. Data were collected using Clampex 10.6 software (Axon Instruments, CA, USA) and analyzed using Clampfit 10.6 software (Axon Instruments, CA, USA).

Spontaneous miniature excitatory postsynaptic currents (mEPSCs) mEPSCs were measured under the whole-cell configuration of the voltage clamp. The membrane potential was held at −70 mV. Compensation of cell capacitance and series resistances were performed before recording. The recordings had only a high resistance seal (>1 GΩ) and a series resistance <25 MΩ. To isolate AMPA receptor-mediated mEPSCs, the aCSF was added with 10 nM of glycine, 10 μM of bicuculline (the GABAA receptor antagonist), and D-AP-5 (NMDA receptor antagonist). Meanwhile, 0.5 μM of TTX was included in the extracellular solution.

Neuronal morphology analysis

In vivo dendritic and spine density analysis In vivo dendrites and spine density were analyzed using Golgi Stain Kits. Eight-week-old mouse brains were immersed in Golgi solution A + B (FD Rapid Golgi Stain Kit, PK401, FD NeuroTechnologies), and transferred into Solution C for 3 days at room temperature. Brain tissues were cut into coronal sections (200 μm) by a Leica CM1950 cryostat. All sections were mounted on 3% gelatin-coated slides. After staining, slides were dehydrated in ethanol and mounted with Permount. The dendrites and the second segment apical dendrite spine were imaged using an LSM 710 confocal microscope. The Simple Neurite Tracer plugin of Fiji was used to trace the dendritic branches and calculate their lengths.

In vitro dendritic and spine analysis Cultured neurons at 40 days *in vitro* (DIV-40) were fixed with 4% paraformaldehyde, washed with PBS, and blocked with 2% goat serum containing 0.1% Triton X-100 for 1 h. Neurons were incubated with primary antibodies (MAP2, mab3418, Millipore, 1:1,000) overnight at 4°C, and then incubated with secondary antibodies. Dendrites were imaged using

©2022 The Authors

an LSM710 confocal microscope. Immunostaining neurons at DIV-55 were used for spine analysis. The secondary dendritic spines were imaged using an LSM710 confocal microscope with a 63 × oil lens.

Histology and immunostaining

Brains were collected and fixed overnight with 4% PFA in PBS at 4°C and dehydrated in 30% sucrose in PBS at 4°C. For sections or isolated cells, cryo-sections (40 μm thick) or coverslips were fixed with 4% PFA/PBS, and permeabilized with 0.5% Triton X-100 for 10 min and blocked in blocking buffer (0.3% Triton X-100, 2% Bovine Serum Albumin) for 1 h at room temperature. Primary antibodies were incubated at 4°C overnight. The primary antibodies used in this study are as follows: rabbit anti-Arid1a (1:1,000, HPA005456, Sigma), pig anti-Vgult1 (1:1,000, ab5905, Millipore), rabbit anti-H3K27ac (1:1,000, ab4729, Abcam), chick anti-MAP2 (1:1,000, Cat No.822501, Biolegend), rabbit anti-PSD95(1:1,000, ab16659, Abcam), mouse anti-synaptophysin (1:1,000, ab13552, Abcam), mouse anti-Oct-3/4 (1;1,000, Cat No. sc-5,279, Santa Cruz), rabbit anti-Nanog (1:1,000, Cat No. 14295–1, Proteintech), and rat anti-BrdU (1:1,000, Cat No. ab6326, Abcam). Sections or coverslips were washed in PBS and incubated with secondary Alexa Fluor-conjugated antibodies (Invitrogen) for 1.5 h at room temperature, washed three times in PBS, and mounted with an adhesion antifade medium. We used a confocal laser-scanning microscope to obtain images and ImageJ to analyze the results.

RNA isolation and quantitative RT-PCR

Total RNA from mice brain tissues or cultured cells was lysed using 1 mL of Trizol reagent according to the manufacturer's instructions (Invitrogen). RNA was then reverse transcribed into cDNA with TransScript One-Step gDNA Removal and cDNA Synthesis Kit (TRANS). The cDNA was amplified using quantitative SYBR® *Premix Ex Taq*™ (Tli RNaseH Plus; Takara). Quantitative real-time PCR (qPCR) was performed using the two-step method RT-PCR system (Roche). Each sample was performed in triplicate. mRNA levels were normalized to values for GAPDH using the $2^{-\Delta\Delta CT}$ method. qPCR primer sequences are listed in Appendix Table S2 (Appendix).

Western blot

Western blot analysis was performed as previously published (Liu *et al*, 2017). The brain tissues were lysed in cold RIPA buffer (Beyotime, P0013B) containing a protease inhibitor cocktail (Roche). After centrifugation, the supernatants of the sample's concentration were measured using a BCA protein assay kit (Biomed, PP0102). Protein samples were loaded in 8–15% SDS-PAGE gels (Bio-Rad) and transferred into PVDF membranes (Millipore). The membrane was blocked with 5% milk in TBST (TBS + 0.05% Tween-20) for 1 h at room temperature and incubated at 4°C overnight with primary antibodies. The following antibodies were used: anti-tubulin (HRP Conjugated; 1:5,000, BE3312, EASYBIO), mouse anti-β-actin (1:10,000, A5441, Sigma), rabbit anti-Arid1a (1:2,000, HPA005456, Sigma), rabbit anti-H3K27ac (1:1,000, ab4729, Abcam), rabbit anti-PSD95(1:1,000, ab16659, Abcam), mouse anti-Oct-3/4 (1;1,000, Cat No. sc-5,279, Santa Cruz), rabbit anti-Nanog (1:1,000, Cat No. 14295–1, Proteintech), rabbit recombinant anti-Glutamate Receptor 1 (AMPA subtype) antibodies (1:1,000, ab109450, Abcam), and rabbit recombinant anti-CaMKII antibodies (1:1,000, ab52476, Abcam). Secondary antibodies conjugated to HRP were incubated at room temperature for 1 h. Protein bands were visualized by enhanced chemiluminescence reagent (ECL, Pierce) and quantified using ImageJ.

Chromatin immunoprecipitation (ChIP)

ChIP was performed as described earlier. In brief, tissues isolated from Arid1a$^{fl/+}$and cHet were cross-linked with 1% formaldehyde (Sigma-Aldrich) for 10 min at room temperature and quenched with 0.125 M glycine for 5 min. Cross-linked samples were washed with cold PBS and suspended in 500 μl of nuclei lysis buffer (50 mM Tris, pH 8.1, 10 mM EDTA, 1% SDS, 1 × protease inhibitor cocktail), followed by sonication for 30 min at 4°C to achieve fragments of sizes ranging from 200 to 500 bp. 1% lysate was maintained to quantify DNA before immunoprecipitation (input). Immunoprecipitation was performed at 4°C overnight. Antibodies used include normal rabbit IgG (ChIP grade, 2729, Cell Signaling), and rabbit polyclonal to H3K27ac (ChIP grade,ab4729, Abcam). After incubation, chromatin pulled down by the Protein A–bound antibodies were washed in IP dilution buffer, TSE-500 solution (0.1% SDS, 1% Triton X-100, 2 mM EDTA, 20 mM Tris, pH 8.1, 500 mM NaCl), Li/Cl wash solution (100 mM Tris, pH 8.1, 300 mM LiCl, 1% NP-40, 1% deoxycholic acid), and 1× Tris-EDTA buffer (TE), two times each at 4°C. Protein–DNA complexes were decross-linked in IP elution buffer (50 mM NaHCO3 and 1% SDS) and incubated with proteinase K (20 mg/ml) and RNase A at 65°C overnight. DNA was extracted with phenol/chloroform/isoamyl alcohol (25:24:1) isolations, precipitated with ethanol and 10 μg linear acrylamide, and resuspended in nuclease-free water.

Human embryonic stem cell (hESC) culture and neural differentiation

The hESC line H9 was provided by Dr. Baoyang Hu at the Institute of Zoology, Chinese Academy of Sciences. We regularly used the PCR detection method to test for mycoplasma contamination. H9 cells were cultivated on Matrigel (BD Biosciences)-coated six-well plates and maintained in TesR-E8 medium (STEMCELL Technologies, Vancouver, BC, Canada). For neural differentiation, H9 cells were dissociated to single cells with accutase (BD Biosciences) and maintained in N2 medium (DMEMF/12, 1 × N2, 1 × NEAA) containing 10 uM Rock inhibitor (Y27632, Selleck, Shanghai, China) from day 0 to day 7, then maintained in N2 medium with 1ug/mL laminin for 7 days. The medium was changed every other day. After 14 days, canonical neural rosettes appeared and were picked. These neural rosettes were induced as neurospheres in an N2B27 medium (DMEM/F12, 1 × N2, 1 × B27, 0.2 mM RA, and 20 ng/mL FGF2) until day 20. The neurospheres were collected by dissociation with accutase and replated in a differentiation medium, including DMEM/F12, 1 × N2, 1 × B27, 10 ng/mL BDNF, 10 ng/mL GDNF, 1 mM cAMP, and 200 nM of ascorbic acid on poly-ornithine and laminin-coated 24-well plates and maintained for up to 40–60 d with a medium change every 2 days.

©2022 The Authors

Construction of the RNA-Guided CRISPR/Cas9 vector and gene knock-out in hESCs

We designed specific guide RNA sequences (sgRNAs) targeting exon 1 of ARID1A on the website (http://crispr.mit.edu). Then, sgRNA were cloned to the PX330-GFP-U6 plasmid (a gift from Dr. Haoyi Wang at the Institute of Zoology, Chinese Academy of Sciences) containing the Cas9 protein sequence and guide RNA.

H9 cells at 70–80% confluence were separated into single cells using accutase at 37°C for 4 min. Prior to electroporation, 10 μg gRNA expression plasmid was diluted with Primary cell solution (Lonza) and was first made a nucleofection solution. For each reaction, cells were mixed with the nucleofection solution (Lonza). Nucelofection was performed in a Nucleofector II device (Lonza) using the program CM115. After electroporation, cells were immediately resuspended in an hESC medium with 10 uM Rock inhibitor (Y-27632) and transferred into a matrigel-coated 6-well plate. To measure the targeted editing frequency, cells were screened to obtain GFP-positive with flow cytometry (BD Bioscience) after 36 h of culture and reseeded as single cells. After approximately 2 weeks, colonies were picked and expanded for genotype sequencing with the following primers to check for integration: Arid1a-372 bp-F, 5′-GGGAGAAGACGAAGACAGGG-3′, Arid1a-372 bp-R, 5′-CGTTCCCGTTCGAGTTCTTC-3′.

ChIP-seq data analysis

ChIP-seq libraries were sequenced, generating 50-bp single reads. We used Trimmomatic (v.0.36) and quality control was performed using FastQC (v. 0.11.7) to filter raw reads data (Bolger *et al*, 2014). High-quality reads were aligned using Bowtie 2 (v2.3.5.1) to the mouse reference genome using default parameters (Langmead & Salzberg, 2012). Samtools (v.1.9) was then used to transfer files to bam format and filter reads with parameters “-F 1804 -q 30” for single-end sequencing data (Li *et al*, 2009). PCR duplicates were removed using the Mark Duplicates function in Picard (v.2.21.2; http://broadinstitute.github.io/picard/) and mitochondrial reads were also removed. MACS (v.2.2.5) was used to call peaks (−q 0.01) relative to the input sample (Feng *et al*, 2012).

MAnorm (v.1.2.0) was designed for the quantitative comparison of ChIP-Seq data (Shao *et al*, 2012). Different binding of H3K27ac peaks was defined by P-values < 0.05 and absolute M-values exceeding 1.5. Peak annotation was performed using ChIPseeker (v.1.22.1) at the gene level and promoter regions were demarcated as +/− 1,000 bp of TSS(Yu *et al*, 2015). DeepTools (v. 3.3.1) “computeMatrix,” “plotHeatmap,” and “plotProfile” functions were used to generate heatmaps and profile plots (Ramírez *et al*, 2016). For genome browser representation, IGV (v. 2.4.10) was used to visualize data in bigwig files generated by deepTools (Thorvaldsdottir *et al*, 2013). The mouse reference genome sequence (vM23) and gene annotation (vM23) was achieved from GENCODE (https://www.gencodegenes.org/).

RNA-seq data analysis

We performed pseudo-replicates of FASTQ files according to the uniform processing pipeline (https://github.com/ENCODE-DCC/atac-seq-pipeline/blob/master/src/encode_task_spr.py) to reduce the effect of library construction bias and sequencing depth. In brief, the two biological replicates were merged and arbitrarily divided into two equal parts called pseudo-replicates (Fu *et al*, 2018; Yan *et al*, 2020). We used FastQC to assess sequencing quality control and used Salmon (v.1.0.0) with the parameter “--validateMappings --gcBias -g” to quantify high-quality RNA-seq reads (Patro *et al*, 2017). Differential gene expression analysis was generated using DESeq2 (v1.26.0) and differentially expressed genes were conducted by P.adjust < 0.05 and absolute fold change more than 1.5 (Love *et al*, 2014). We used clusterProfiler to perform gene enrichment analysis (Yu *et al*, 2012).

> **The paper explained**
>
> **Issue**
> Mutations in AT-rich interactive domain-containing protein 1A (ARID1A) cause Coffin-Siris syndrome (CSS), a rare genetic disorder that can result in mild-to-severe intellectual disabilities. However, the biological role of ARID1A in the brain remains unclear. Currently, little is known about the cellular and molecular mechanisms underlying Intellectual Disabilities (ID) caused by ARID1A mutations, or how to ameliorate related phenotypes.
>
> **Results**
> We established a mouse model with specific *Arid1a* deletion in excitatory neurons and found that *Arid1a* haploinsufficiency in excitatory neurons of the forebrain led to a reduction of neurite complexity, dysfunction of synaptic plasticity, and impairment of mouse spatial memory. Using CRISPR-CAS9 knockout technology, we constructed *ARID1A* KO hESC cell lines and demonstrated that *ARID1A* KO hESC-derived excitatory neurons exhibited a severe reduction in neurite complexity and abnormal synaptic plasticity. Transcriptomic and ChIP-seq analyses demonstrated that ARID1A haploinsufficiency in excitatory neurons suppressed the histone H3 lysine 27 acetylation (H3K27ac) mark at key neuronal genes associated with synapses or cognition. Acetate supplementation could reverse the defects caused by the *Arid1a* mutant in mice and *ARID1A* KO hESC-derived neurons by regulating H3K27 acetylation levels in the promoters of neuronal genes.
>
> **Impact**
> Our findings have significant implications for the role of ARID1A in excitatory neurons and spatial memory. Acetate supplementation by increasing H3K27 acetylation levels could serve as an intervention for treating ID or CSS caused by *ARID1A* mutations.

Quantification of H3K27ac immunofluorescence

To confirm all the cells analyzed were excitatory neurons, we performed co-immunostaining of H3K27ac with an excitatory neuron marker Vglut1. Only Vglut1 positive cells were selected to measure their relative fluorescent intensities. To determine the relative fluorescent intensities, fluorescence images were captured at the same exposure time using a Zeiss LSM880 confocal microscope. Area, integrated density and mean gray values of at least 120 randomly selected nuclei in the cortex (150–400 μm from the pia mater) from 3 animals per group were measured. The mean fluorescence of small areas that had no fluorescence was calculated as the background reading for every image. The corrected total cell fluorescence was determined by the following equation: the Corrected

©2022 The Authors

Total Cell Fluorescence = Integrated Density – (Area of Selected Cell x Mean Fluorescence of Background readings).

Statistical analysis

We applied Shapiro–Wilk and Kolmogorov–Smirnov tests to assess the normal distributions of datasets. All datasets passed the normality test in this study. Statistical analysis of the data was performed using GraphPad Prism 8 software with student's *t*-test (*t*-test), one-way, or two-way analysis of covariance as specified in the diagram of each figure. All data were presented as mean ± SEM, and statistical significance was defined as $*P < 0.05$; $**P < 0.01$; $***P < 0.001$.

Data availability

The RNA-seq and ChIP-seq data used in this study have been deposited in the Gene Expression Omnibus (GEO) under accession number GSE198916 (https://www.ncbi.nlm.nih.gov/geo/query/acc.cgi?acc=GSE198916).

Expanded View for this article is available online.

Acknowledgments
This work was supported by grants from the National Key Research and Development Program of China Project (2021YFA1101402/2018YFA0108001), the Strategic Priority Research Program of the Chinese Academy of Sciences (XDA16010300/XDA16021400), the National Science Foundation of China (82271428/31900690), and the Open Project Program of State Key Laboratory of Stem Cell and Reproductive Biology.

Author contributions
Chang-Mei Liu: Conceptualization; data curation; funding acquisition; writing – original draft; writing—review and editing. **Pei-Pei Liu:** Conceptualization; data curation; formal analysis; methodology; writing—original draft. **Shang-Kun Dai:** Data curation; validation; methodology. **Ting-Wei Mi:** Data curation; validation; methodology. **Gang-Bin Tang:** Conceptualization; formal analysis. **Zhuo Wang:** Formal analysis; validation. **Hui Wang:** Formal analysis; validation. **Hong-Zhen Du:** Formal analysis; validation. **Yi Tang:** Project administration. **Zhao-Qian Teng:** Conceptualization; data curation; validation; investigation; project administration; writing – review and editing.

Disclosure and competing interests statement
The authors declare that they have no conflict of interest.

References

Bartsocas CS, Tsiantos AK (1970) Mental retardation with absent fifth fingernail and terminal phalanx. *Am J Dis Child* 120: 493 – 494

Bidart M, El Atifi M, Miladi S, Rendu J, Satre V, Ray PF, Bosson C, Devillard F, Lehalle D, Malan V *et al* (2017) Microduplication of the ARID1A gene causes intellectual disability with recognizable syndromic features. *Genet Med* 19: 701 – 710

Bogershausen N, Wollnik B (2018) Mutational landscapes and phenotypic Spectrum of SWI/SNF-related intellectual disability disorders. *Front Mol Neurosci* 11: 252

Bohnsack JP, Patel VK, Morrow AL (2017) Ethanol exposure regulates Gabra1 expression via histone deacetylation at the promoter in cultured cortical neurons. *J Pharmacol Exp Ther* 363: 1 – 11

Bolger AM, Lohse M, Usadel B (2014) Trimmomatic: a flexible trimmer for Illumina sequence data. *Bioinformatics* 30: 2114 – 2120

Bravo-Hernandez M, Tadokoro T, Navarro MR, Platoshyn O, Kobayashi Y, Marsala S, Miyanohara A, Juhas S, Juhasova J, Skalnikova H *et al* (2020) Spinal subpial delivery of AAV9 enables widespread gene silencing and blocks motoneuron degeneration in ALS. *Nat Med* 26: 118 – 130

Bultman S, Gebuhr T, Yee D, La Mantia C, Nicholson J, Gilliam A, Randazzo F, Metzger D, Chambon P, Crabtree G *et al* (2000) A Brg1 null mutation in the mouse reveals functional differences among mammalian SWI/SNF complexes. *Mol Cell* 6: 1287 – 1295

Cheng Y, Wang ZM, Tan W, Wang X, Li Y, Bai B, Li Y, Zhang SF, Yan HL, Chen ZL *et al* (2018) Partial loss of psychiatric risk gene Mir137 in mice causes repetitive behavior and impairs sociability and learning via increased Pde10a. *Nat Neurosci* 21: 1689 – 1703

Clapier CR, Cairns BR (2009) The biology of chromatin remodeling complexes. *Annu Rev Biochem* 78: 273 – 304

Cramer SL, Miller AL, Pressey JG, Gamblin TL, Beierle EA, Kulbersh BD, Garcia PL, Council LN, Radhakrishnan R, Hendrix SV *et al* (2017) Pediatric anaplastic embryonal rhabdomyosarcoma: targeted therapy guided by genetic analysis and a patient-derived xenograft study. *Front Oncol* 7: 327

Diana CH, Gerald RC (2011) ATP-dependent chromatin remodeling: genetics, genomics and mechanisms. *Cell Res* 21: 396 – 420

Feng JX, Liu T, Qin B, Zhang Y, Liu XS (2012) Identifying ChIP-seq enrichment using MACS. *Nat Protoc* 7: 1728 – 1740

Fu S, Wang Q, Moore JE, Purcaro MJ, Pratt HE, Fan K, Gu C, Jiang C, Zhu R, Kundaje A *et al* (2018) Differential analysis of chromatin accessibility and histone modifications for predicting mouse developmental enhancers. *Nucleic Acids Res* 46: 11184 – 11201

Gao X, Tate P, Hu P, Tjian R, Skarnes WC, Wang Z (2008) ES cell pluripotency and germ-layer formation require the SWI/SNF chromatin remodeling component BAF250a. *Proc Natl Acad Sci U S A* 105: 6656 – 6661

Goebbels S, Bormuth I, Bode U, Hermanson O, Schwab MH, Nave KA (2006) Genetic targeting of principal neurons in neocortex and hippocampus of NEX-Cre mice. *Genesis* 44: 611 – 621

Gorski JA, Talley T, Qiu MS, Puelles L, Rubenstein JLR, Jones KR (2002) Cortical excitatory neurons and glia, but not GABAergic neurons, are produced in the Emx1-expressing lineage. *J Neurosci* 22: 6309 – 6314

Graff J, Rei D, Guan JS, Wang WY, Seo J, Hennig KM, Nieland TJ, Fass DM, Kao PF, Kahn M *et al* (2012) An epigenetic blockade of cognitive functions in the neurodegenerating brain. *Nature* 483: 222 – 226

Hordeaux J, Yuan Y, Clark PM, Wang Q, Martino RA, Sims JJ, Bell P, Raymond A, Stanford WL, Wilson JM (2019) The GPI-linked protein LY6A drives AAV-PHP.B transport across the blood-brain barrier. *Mol Ther* 27: 912 – 921

Jin SY, Cao Q, Yang FH, Zhu HY, Xu S, Chen Q, Wang ZY, Lin YH, Cinar R, Pawlosky RJ *et al* (2021) Brain ethanol metabolism by astrocytic ALDH2 drives the behavioural effects of ethanol intoxication. *Nat Metab* 3: 337 – 351

Kim TH, Yoo JY, Wang Z, Lydon JP, Khatri S, Hawkins SM, Leach RE, Fazleabas AT, Young SL, Lessey BA *et al* (2015) ARID1A is essential for endometrial function during early pregnancy. *PLoS Genet* 11: e1005537

Kosho T, Okamoto N, Ohashi H, Tsurusaki Y, Imai Y, Hibi-Ko Y, Kawame H, Homma T, Tanabe S, Kato M *et al* (2013) Clinical correlations of mutations affecting six components of the SWI/SNF complex: detailed description of 21 patients and a review of the literature. *Am J Med Genet A* 161A: 1221 – 1237

 ©2022 The Authors

Kosho T, Okamoto N, Coffin-Siris Syndrome International Collaborators (2014) Genotype-phenotype correlation of coffin-Siris syndrome caused by mutations in SMARCB1, SMARCA4, SMARCE1, and ARID1A. *Am J Med Genet C Semin Med Genet* 166C: 262 – 275

Langmead B, Salzberg SL (2012) Fast gapped-read alignment with bowtie 2. *Nat Methods* 9: 357 – 359

Li H, Handsaker B, Wysoker A, Fennell T, Ruan J, Homer N, Marth G, Abecasis G, Durbin R, 1000 Genome Project Data Processing Subgroup (2009) The sequence alignment/map format and SAMtools. *Bioinformatics* 25: 2078 – 2079

Li MY, Miao WY, Wu QZ, He SJ, Yan GQ, Yang YR, Liu JJ, Taketo MM, Yu X (2017) A critical role of presynaptic cadherin/catenin/p140Cap complexes in stabilizing spines and functional synapses in the neocortex. *Neuron* 94: 1155 – 1172.e8

Li ZH, Zhang CK, Qiao Y, Ge SN, Zhang T, Li JL (2020) Coexpression of VGLUT1 and VGLUT2 in precerebellar neurons in the lateral reticular nucleus of the rat. *Brain Res Bull* 162: 94 – 106

Lin WC, Tsai MC, Davenport CM, Smith CM, Veit J, Wilson NM, Adesnik H, Kramer RH (2015) A comprehensive optogenetic pharmacology toolkit for In vivo control of GABA(a) receptors and synaptic inhibition. *Neuron* 88: 879 – 891

Liu PP, Tang GB, Xu YJ, Zeng YQ, Zhang SF, Du HZ, Teng ZQ, Liu CM (2017) MiR-203 interplays with Polycomb repressive complexes to regulate the proliferation of neural stem/progenitor cells. *Stem Cell Reports* 9: 190 – 202

Liu X, Dai SK, Liu PP, Liu CM (2021) Arid1a regulates neural stem/progenitor cell proliferation and differentiation during cortical development. *Cell Prolif* 54: e13124

Love MI, Huber W, Anders S (2014) Moderated estimation of fold change and dispersion for RNA-seq data with DESeq2. *Genome Biol* 15: 550

Mathur R, Alver BH, San Roman AK, Wilson BG, Wang X, Agoston AT, Park PJ, Shivdasani RA, Roberts CW (2017) ARID1A loss impairs enhancer-mediated gene regulation and drives colon cancer in mice. *Nat Genet* 49: 296 – 302

Maulik PK, Mascarenhas MN, Mathers CD, Dua T, Saxena S (2013) Prevalence of intellectual disability: a meta-analysis of population-based studies (vol 32, pg 419, 2011). *Res Dev Disabil* 34: 729

Moussaieff A, Rouleau M, Kitsberg D, Cohen M, Levy G, Barasch D, Nemirovski A, Shen-Orr S, Laevsky I, Amit M *et al* (2015) Glycolysis-mediated changes in acetyl-CoA and histone acetylation control the early differentiation of embryonic stem cells. *Cell Metab* 21: 392 – 402

Mukai J, Cannavo E, Crabtree GW, Sun Z, Diamantopoulou A, Thakur P, Chang CY, Cai Y, Lomvardas S, Takata A *et al* (2019) Recapitulation and reversal of schizophrenia-related phenotypes in Setd1a-deficient mice. *Neuron* 104: 471 – 487.e12

Naidu SR, Love IM, Imbalzano AN, Grossman SR, Androphy EJ (2009) The SWI/SNF chromatin remodeling subunit BRG1 is a critical regulator of p53 necessary for proliferation of malignant cells. *Oncogene* 28: 2492 – 2501

Patro R, Duggal G, Love MI, Irizarry RA, Kingsford C (2017) Salmon provides fast and bias-aware quantification of transcript expression. *Nat Methods* 14: 417 – 419

Peleg S, Sananbenesi F, Zovoilis A, Burkhardt S, Bahari-Javan S, Agis-Balboa RC, Cota P, Wittnam JL, Gogol-Doering A, Opitz L *et al* (2010) Altered histone acetylation is associated with age-dependent memory impairment in mice. *Science* 328: 753 – 756

Ramírez F, Ryan D, Grüning B, Bhardwaj V, Kilpert F, Richter A, Heyne S, Dündar F, Manke T (2016) deepTools2: a next generation web server for deep-sequencing data analysis. *Nucleic Acids Res* 44: W160 – W165

Rotaru DC, van Woerden GM, Wallaard I, Elgersma Y (2018) Adult Ube3a gene reinstatement restores the electrophysiological deficits of prefrontal cortex layer 5 neurons in a mouse model of Angelman syndrome. *J Neurosci* 38: 8011 – 8030

Rotterman TM, Nardelli P, Cope TC, Alvarez FJ (2014) Normal distribution of VGLUT1 synapses on spinal motoneuron dendrites and their reorganization after nerve injury. *J Neurosci* 34: 3475 – 3492

Santen GW, Aten E, Vulto-van Silfhout AT, Pottinger C, van Bon BW, van Minderhout IJ, Snowdowne R, van der Lans CA, Boogaard M, Linssen MM *et al* (2013) Coffin-Siris syndrome and the BAF complex: genotype-phenotype study in 63 patients. *Hum Mutat* 34: 1519 – 1528

Schwanhausser B, Busse D, Li N, Dittmar G, Schuchhardt J, Wolf J, Chen W, Selbach M (2011) Global quantification of mammalian gene expression control. *Nature* 473: 337 – 342

Shao Z, Zhang Y, Yuan GC, Orkin SH, Waxman DJ (2012) MAnorm: a robust model for quantitative comparison of ChIP-seq data sets. *Genome Biol* 13: R16

Sheng M (2001) Molecular organization of the postsynaptic specialization. *Proc Natl Acad Sci U S A* 98: 7058 – 7061

Singh S, Choudhury A, Gusain P, Parvez S, Palit G, Shukla S, Ganguly S (2016) Oral acetate supplementation attenuates N-methyl D-aspartate receptor hypofunction-induced behavioral phenotypes accompanied by restoration of acetyl-histone homeostasis. *Psychopharmacology* 233: 1257 – 1268

Slavotinek A, Lefebvre M, Brehin AC, Thauvin C, Patrier S, Sparks TN, Norton M, Yu J, Huang E (2022) Prenatal presentation of multiple anomalies associated with haploinsufficiency for ARID1A. *Eur J Med Genet* 65: 104407

Son EY, Crabtree GR (2014) The role of BAF (mSWI/SNF) complexes in mammalian neural development. *Am J Med Genet C Semin Med Genet* 166C: 333 – 349

Thorvaldsdottir H, Robinson JT, Mesirov JP (2013) Integrative genomics viewer (IGV): high-performance genomics data visualization and exploration. *Brief Bioinform* 14: 178 – 192

Tillotson R, Selfridge J, Koerner MV, Gadalla KKE, Guy J, De Sousa D, Hector RD, Cobb SR, Bird A (2017) Radically truncated MeCP2 rescues Rett syndrome-like neurological defects. *Nature* 550: 398 – 401

Tsurusaki Y, Okamoto N, Ohashi H, Kosho T, Imai Y, Hibi-Ko Y, Kaname T, Naritomi K, Kawame H, Wakui K *et al* (2012) Mutations affecting components of the SWI/SNF complex cause coffin-Siris syndrome. *Nat Genet* 44: 376 – 378

Vasko A, Drivas TG, Schrier Vergano SA (2021) Genotype-phenotype correlations in 208 individuals with coffin-Siris syndrome. *Genes* 12: 937

Vissers LELM, Gilissen C, Veltman JA (2016) Genetic studies in intellectual disability and related disorders. *Nat Rev Genet* 17: 9 – 18

Wang L, Maffei A (2014) Inhibitory plasticity dictates the sign of plasticity at excitatory synapses. *J Neurosci* 34: 1083 – 1093

Wilson BG, Roberts CW (2011) SWI/SNF nucleosome remodellers and cancer. *Nat Rev Cancer* 11: 481 – 492

Wilson MR, Reske JJ, Holladay J, Neupane S, Ngo J, Cuthrell N, Wegener M, Rhodes M, Adams M, Sheridan R *et al* (2020) ARID1A mutations promote P300-dependent endometrial invasion through super-enhancer hyperacetylation. *Cell Rep* 33: 108366

Yan P, Liu Z, Song M, Wu Z, Xu W, Li K, Ji Q, Wang S, Liu X, Yan K *et al* (2020) Genome-wide R-loop landscapes during cell differentiation and reprogramming. *Cell Rep* 32: 107870

Yu GC, Wang LG, Han YY, He QY (2012) clusterProfiler: an R package for comparing biological themes among gene clusters. *OMICS* 16: 284 – 287

Yu G, Wang LG, He QY (2015) ChIPseeker: an R/Bioconductor package for ChIP peak annotation, comparison and visualization. *Bioinformatics* 31: 2382 – 2383

Zheng Y, Liu A, Wang ZJ, Cao Q, Wang W, Lin L, Ma K, Zhang F, Wei J, Matas E *et al* (2019) Inhibition of EHMT1/2 rescues synaptic and cognitive functions for Alzheimer's disease. *Brain* 142: 787 – 807

Zhou YG, Schmitz KM, Mayer C, Yuan XJ, Akhtar A, Grummt I (2009) Reversible acetylation of the chromatin remodelling complex NoRC is required for non-coding RNA-dependent silencing. *Nat Cell Biol* 11: 1010 – U1229

License: This is an open access article under the terms of the Creative Commons Attribution License, which permits use, distribution and reproduction in any medium, provided the original work is properly cited.

©2022 The Authors

Expanded View Figures

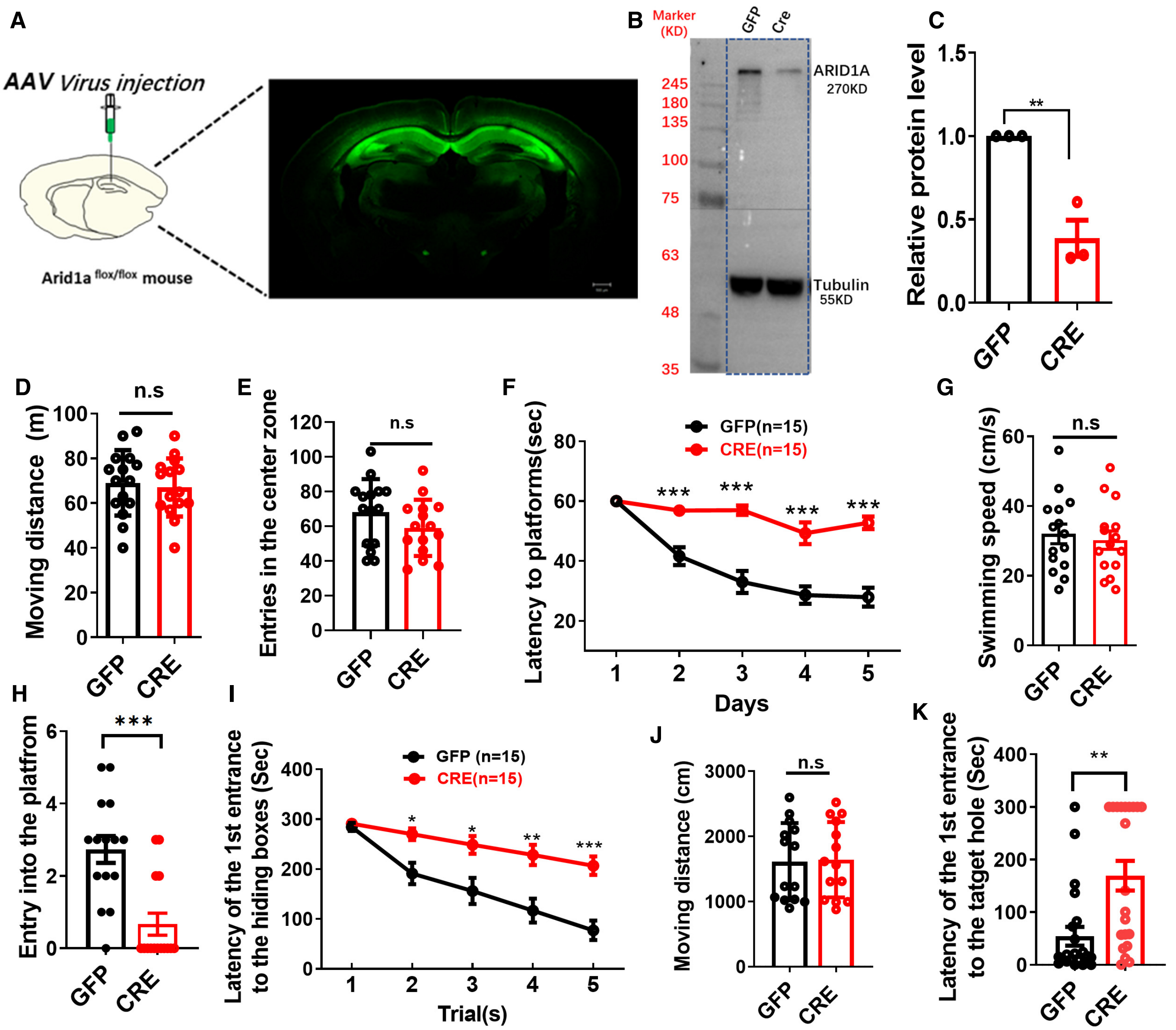

Figure EV1.

Figure EV1. Reduced expression of *Arid1a* exhibits impaired learning and memory disability.

A Schematic illustrating injection of AAV-CRE-GFP or AAV-GFP control virus in adult $Arid1a^{fl/fl}$ hippocampus (left), representative images showing GFP expression in the hippocampus after 1-month viral injection (right). Scale bar, 500 μm.
B Representative Western blot images for ARID1A expression. ARID1A expression is downregulated in the hippocampus after AAV-*Cre* injection. Tubulin was used as a loading control.
C Quantification of ARID1A protein levels in hippocampal tissues from GFP and AAV-*Cre* mice (*n* = 3 mice).
D AAV-*Cre* mice had comparable locomotion to GFP littermate mice in open field test over a 30-min period (AAV-GFP, *n* = 15 mice; AAV-*Cre*, *n* = 15 mice).
E AAV-*Cre* mice had comparable entries into the center zone during a 30-min open field test compared to GFP littermate mice (AAV-GFP, *n* = 15 mice; AAV-*Cre*, *n* = 15 mice; n.s., nonsignificant).
F AAV-*Cre* mice spent more time reaching the platform during the 5-day training in the Morris water maze test (AAV-GFP, *n* = 15 mice; AAV-*Cre*, *n* = 15 mice).
G AAV-*Cre* mice showed similar swimming speed in the Morris water maze test compared to GFP mice (AAV-GFP, *n* = 15 mice; AAV-*Cre*, *n* = 15 mice).
H AAV-*Cre* mice crossed the platform less frequently in the Morris water maze test (AAV-GFP, *n* = 15 mice; AAV-*Cre*, *n* = 15 mice).
I AAV-*Cre* mice spent more time finding the escape box during 5-day training in the Barnes maze test (AAV-GFP, *n* = 15 mice; AAV-*Cre*, *n* = 15 mice).
J AAV-*Cre* mice showed similar moving distances in the Morris water maze test compared to GFP mice (AAV-GFP, *n* = 15 mice; AAV-*Cre*, *n* = 15 mice).
K AAV-*Cre* mice showed longer latency when finding the escape box in the Barnes maze test (AAV-GFP, *n* = 15 mice; AAV-*Cre*, *n* = 15 mice).

Data information: Data represent means ± SEM. In (C–E), $**P < 0.01$, n.s. = nonsignificant (unpaired two-tailed *t*-test). In (F), $P > 0.9999$ (day 1), $***P < 0.001$ (day 2), $***P < 0.001$ (day 3), $***P < 0.001$ (day 4), $***P < 0.001$ (day 5; two-way ANOVA with Bonferroni *post hoc* test). In (G, H), n.s. = nonsignificant, $***P < 0.001$(unpaired two-tailed *t*-test). In (I), $P > 0.9999$ (Trial1), $*P < 0.05$(Trial 2), $*P < 0.05$ (Trial 3), $**P < 0.01$ (Trial 4), $***P < 0.001$ (Trial 5; two-way ANOVA with Bonferroni *post hoc* test). In (J, K), n.s. = nonsignificant, $**P < 0.01$(unpaired two-tailed *t*-test).
Source data are available online for this figure.

Figure EV2. Supplementation of Acetate rescues neuronal morphology deficits caused by *Arid1a* haploinsufficiency in excitatory neurons.

A Change in the body weight of mice after chronic treatment with ethyl acetate (*n* = 5 mice per group).
B Separate channels of the triple immunostainings staining of DAPI (blue), H3K27ac (green), and Vgult1 (red) in forebrain tissues of $Arid1a^{fl/+}$ and cHet mice, respectively. IF staining was performed on 40-μm thick floating sections. Relative fluorescence intensities of H3K27ac decreased upon the loss of Arid1a in the cortex. Scale Bar, 20 μm.
C Open field test in $Arid1a^{fl/+}$ and *cHet* mice treated with vehicle or Acetate. $Arid1a^{fl/+}$ and cHet mice treated with vehicle or Acetate had similar locomotion in open field tests over a 30-min period (*n* = 15 mice per group).
D $Arid1a^{fl/+}$ and cHet mice treated with vehicle or Acetate displayed similar entry tendencies into the center zone during a 30-min open field test, (*n* = 15 mice per group).
E The digitized trace of Golgi-stained coronal sections in the hippocampal CA1 region of adult (2-months-old) Vehicle or Acetate-treated cHet mice. Scale Bar, 50 μm.
F Quantification of total dendritic length from dendritic tree reconstructions shown in (E)., at least 15 neurons from *n* = 3 mice per group.
G Sholl analysis of dendritic branching complexity in the total dendrites of vehicle or Acetate-treated cHet mice, at least 15 neurons from *n* = 3 mice per group.
H Sholl analysis of dendritic branching complexity in the basal and apical dendrites of vehicle or Acetate-treated cHet mice, at least 15 neurons from *n* = 3 mice per group.

Data information: Data represent means ± SEM. In (C, D), n.s. = nonsignificant, unpaired two-tailed *t*-test. In (F), $**P < 0.01$(apical), $**P < 0.01$(total; unpaired two-tailed *t*-test). In G, $*P < 0.05$ (100 μm), $*P < 0.05$ (110 μm; two-way ANOVA with Bonferroni *post hoc* test). In (H), $*P < 0.05$ (80 μm), $*P < 0.05$ (90 μm), $*P < 0.05$ (100 μm), $**P < 0.01$ (110 μm), $*P < 0.05$ (120 μm), $*P < 0.05$ (130 μm; two-way ANOVA with Bonferroni *post hoc* test).

 ©2022 The Authors

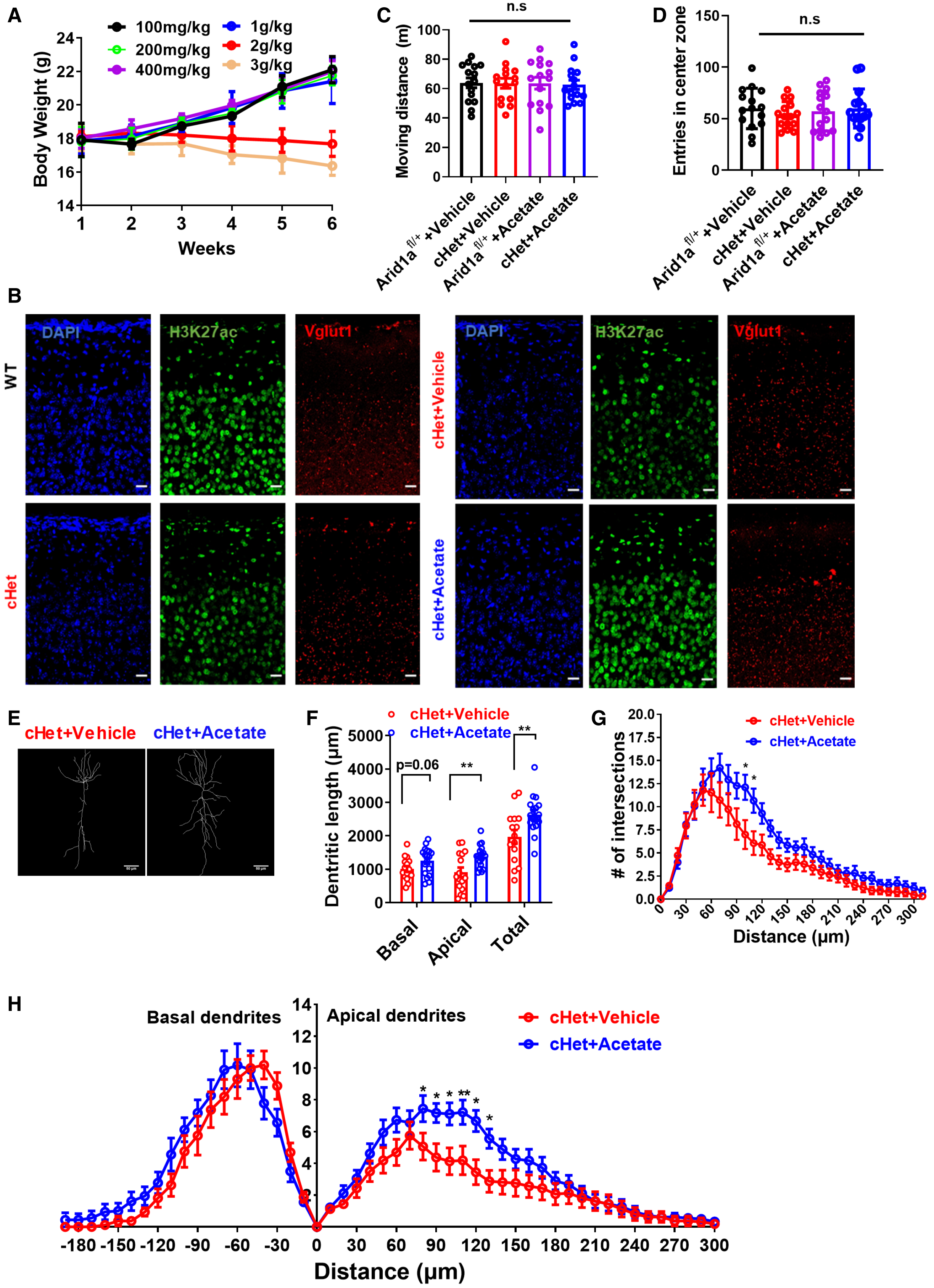
A
Body Weight (g)
Weeks
100mg/kg
200mg/kg
400mg/kg
1g/kg
2g/kg
3g/kg
C
Moving distance (m)
n.s
Arid1a fl/+ +Vehicle
cHet+Vehicle
Arid1a fl/+ +Acetate
cHet+Acetate
D
Entries in center zone
n.s
B
WT
cHet
cHet+Vehicle
cHet+Acetate
DAPI
H3K27ac
Vglut1
E
cHet+Vehicle
cHet+Acetate
F
Dentritic length (μm)
p=0.06
**
Basal
Apical
Total
G
# of intersections
Distance (μm)
H
Basal dendrites
Apical dendrites
Distance (μm)

Figure EV2.

Figure EV3. Altered gene expression profile caused by the haploinsufficiency of *Arid1a*.

A Schematic of RNA-seq experiment. Left, coronal section showing the distribution of excitatory neurons with red fluorescence of tdTomato.
B The principal component analysis (PCA) of RNA-seq.
C GO functional clustering of downregulated targets in Arid1a-haploinsufficiency transcriptome.
D KEGG pathway analysis of upregulated targets in Arid1a-haploinsufficiency transcriptome.
E Principal component analysis (PCA) results of H3K27ac ChIP-seq data based on enrichment signals at peak regions.
F Left, heatmaps displaying enhanced (5061) and reduced (1899) H3K27ac peaks from ChIP-seq analysis in the *Arid1a*$^{fl/+}$ vs cHet mice. Right, Gene ontology (GO) functional clustering of genes that were upregulated and downregulated to identify biological processes regulated by H3K27ac ChIP-seq. The unit of the color key is normalized RPKM.
G Effect of acetate on genome-wide occupancy of H3K27ac as determined by ChIP-seq. The unit of the color key is normalized RPKM.

 ©2022 The Authors

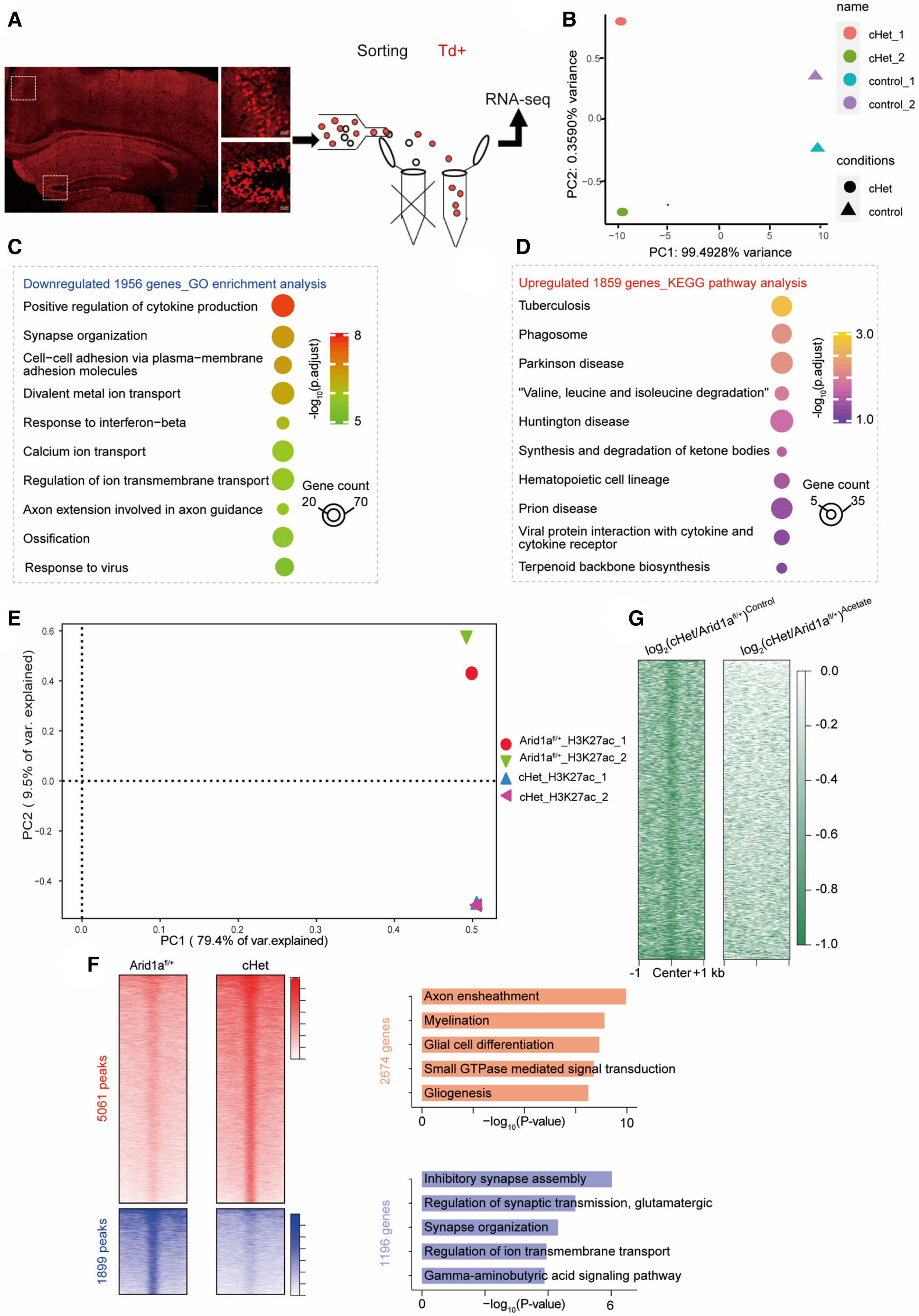
A
Sorting
Td+
RNA-seq
B
name
cHet_1
cHet_2
control_1
control_2
conditions
cHet
control
PC2: 0.3590% variance
PC1: 99.4928% variance
C
Downregulated 1956 genes_GO enrichment analysis
Positive regulation of cytokine production
Synapse organization
Cell−cell adhesion via plasma−membrane adhesion molecules
Divalent metal ion transport
Response to interferon−beta
Calcium ion transport
Regulation of ion transmembrane transport
Axon extension involved in axon guidance
Ossification
Response to virus
-log10(p.adjust)
Gene count
D
Upregulated 1859 genes_KEGG pathway analysis
Tuberculosis
Phagosome
Parkinson disease
"Valine, leucine and isoleucine degradation"
Huntington disease
Synthesis and degradation of ketone bodies
Hematopoietic cell lineage
Prion disease
Viral protein interaction with cytokine and cytokine receptor
Terpenoid backbone biosynthesis
-log10(p.adjust)
Gene count
E
PC2 (9.5% of var. explained)
PC1 (79.4% of var.explained)
Arid1afl/+_H3K27ac_1
Arid1afl/+_H3K27ac_2
cHet_H3K27ac_1
cHet_H3K27ac_2
G
log2(cHet/Arid1afl/+)Control
log2(cHet/Arid1afl/+)Acetate
-1 Center +1 kb
F
Arid1afl/+
cHet
5061 peaks
1899 peaks
2674 genes
Axon ensheathment
Myelination
Glial cell differentiation
Small GTPase mediated signal transduction
Gliogenesis
−log10(P-value)
1196 genes
Inhibitory synapse assembly
Regulation of synaptic transmission, glutamatergic
Synapse organization
Regulation of ion transmembrane transport
Gamma-aminobutyric acid signaling pathway
−log10(P-value)

Figure EV3.

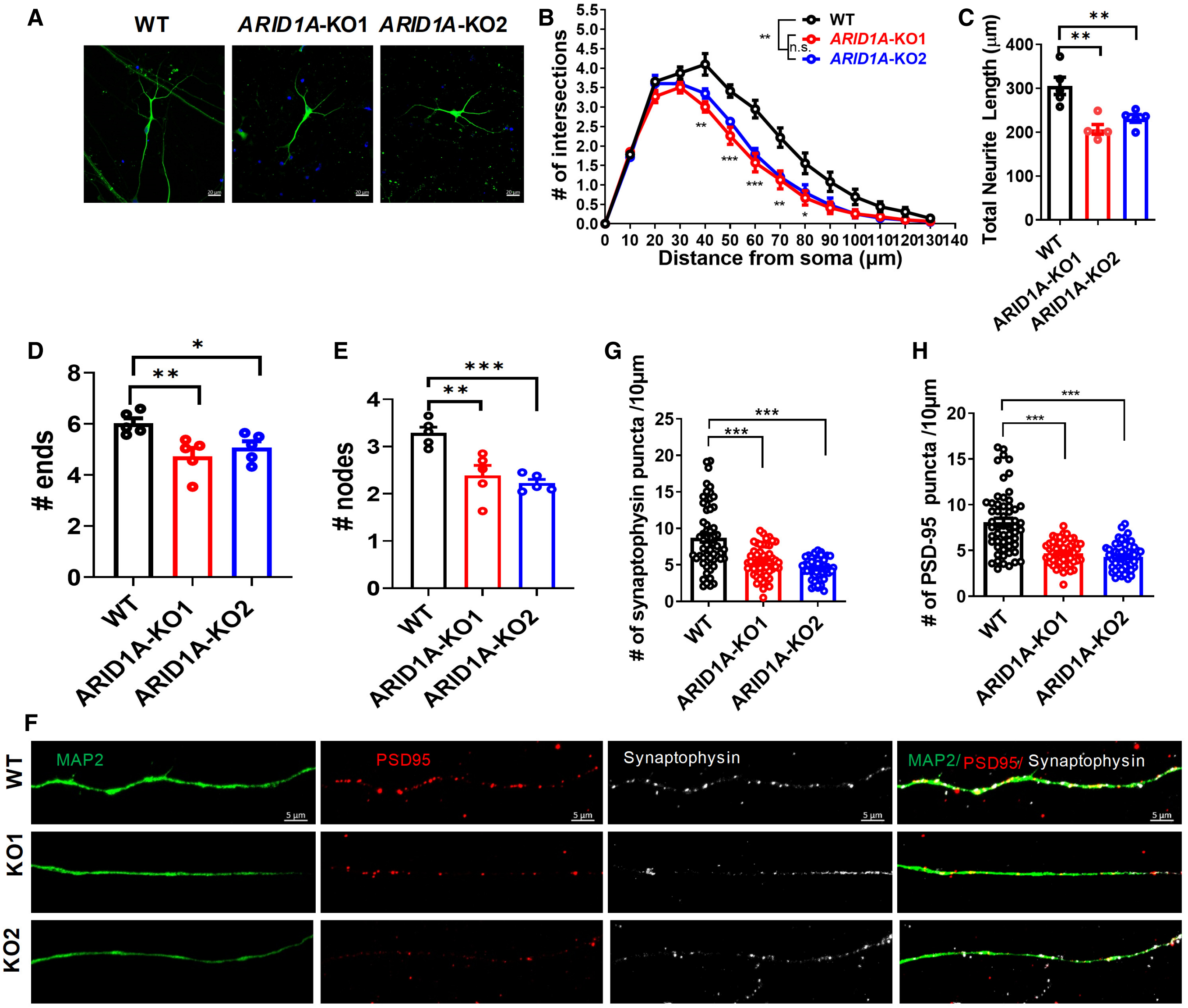

Figure EV4. Loss of ARID1A decreased neurite complexity in hESCs-derived neurons.

A Representative image of neurons derived from WT and *ARID1A* KO hESCs on day 40 of neural differentiation Scale Bar, 20 µm.

B Sholl analysis shows that total neurite length decreased in ARID1A KO hESC-derived neurons on day 40 (*n* = at least 3 independent replicates).

C–E Compared with the WT group, ARID1A KO hESC-derived neurons exhibited decreased dendritic complexity, as shown by reduced length (C), ends (D), and notes (E) (*n* = at least 3 independent replicates).

F Representative images of dendrites (MAP2, green) showing localization of foci of the pre- and postsynaptic protein complexes, synaptophysin, and PSD-95 proteins in WT and ARID1A KO hESC-derived neurons on day 55 Scale Bar, 5 µm.

G, H Quantification of the spine density from synaptophysin (G) and PSD-95 protein (H) stained secondary dendrites of neurons on day 55 were significantly reduced in ARID1A KO hESC on day 55 of neural differentiation (*n* = 3 independent replicates).

Data information: Data represent means ± SEM. In (B), *$P < 0.05$ (40 µm), ***$P < 0.01$(50 µm), ***$P < 0.01$(60 µm), **$P < 0.01$(70 µm), *$P < 0.05$ (80 µm); ANOVA. In (C), **$P < 0.01$ (KO1), **$P < 0.01$ (KO2); unpaired two-tailed *t*-test. In (D), **$P < 0.01$(KO1), *$P < 0.05$ (KO2); unpaired two-tailed *t*-test. In (E), **$P < 0.01$ (KO1), ***$P < 0.001$ (KO2); unpaired two-tailed *t*-test. In (G), ****$P < 0.0001$(KO1), ****$P < 0.0001$(KO2); unpaired two-tailed *t*-test. In (H), ****$P < 0.0001$(KO1), ****$P < 0.0001$(KO2), unpaired two-tailed *t*-test.

 ©2022 The Authors

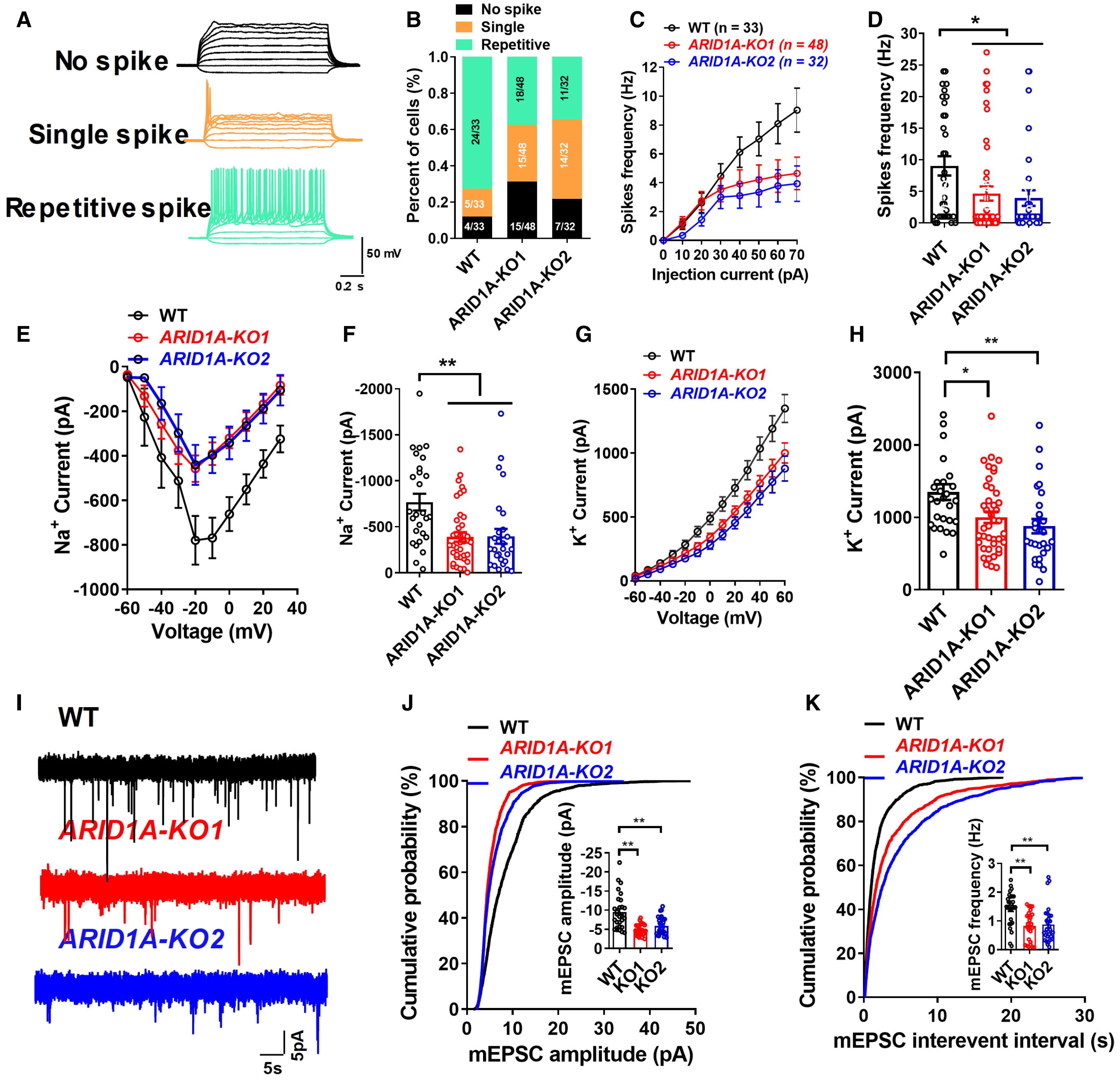

Figure EV5. Loss of ARID1A results in electrophysiological defects and neuronal functions in hESCs-derived neurons.

- A Representative traces of membrane potential responding to step depolarization by current injection steps from −10 pA to +60 pA in 10 pA increments. Membrane potential was current-clamped at around −65 mV. Representative traces were displayed by WT neurons.
- B Quantification of the neuron maturity by recorded AP firing patterns on day 55 after differentiation ($n \geq 30$ neurons in every group).
- C, D Mean input/output relationship during WT and ARID1A hESC-derived neurons on day 55. ($n \geq 30$ neurons in every group).
- E, F Averaged current–voltage relationship (I-V curves) for Na+ currents, recorded from hESC-derived neurons ($n \geq 27$ neurons in every group).
- G, H Averaged current–voltage relationship (I-V curves) for K+ currents, recorded from hESC-derived neurons ($n \geq 27$ neurons in every group).
- I Detection of mEPSCs in whole-cell recordings of WT and ARID1A KO hESC-derived neurons on day 55.
- J Amplitude of SC decreased significantly in ARID1A KO hESC-derived neurons ($n \geq 25$ neurons in every group).
- K Frequency of mEPSC decreased significantly in ARID1A KO hESC-derived neurons ($n \geq 25$ neurons in every group).

Data information: Data represent means ± SEM. In (D, F, H, J, K), *$P < 0.05$, **$P < 0.01$, unpaired two-tailed t-test.
