# Supplementary material for: Acetate supplementation restores cognitive deficits caused by ARID1A haploinsufficiency in excitatory neurons
Source: EMBO Mol Med. 2022 Nov 17;14(12):e15795. doi: 10.15252/emmm.202215795 (PMC9728054; doi:10.15252/emmm.202215795)
Supplement: Supplementary file 8 [file EMMM-14-e15795-s008.pdf]

EMBO Molecular Medicine

Acetate Supplementation Restores Cognitive Deficits Caused by ARID1A Haploinsufficiency in Excitatory Neurons

Chang-Mei Liu, Pei-Pei Liu, Shang-Kun Dai, Ting-Wei Mi, Gang-Bin Tang, Zhuo Wang, Hui Wang, Hong-Zhen Du, Yi Tang, and Zhao-Qian Teng
DOI: 10.15252/emmm.202215795

Corresponding authors: Chang-Mei Liu (liuchm@ioz.ac.cn) , Chang-Mei Liu (liuchm@ioz.ac.cn), Zhao-Qian Teng (tengzq@ioz.ac.cn), Yi Tang (tangyi@xwhosp.org)

Review Timeline:

Submission Date:	28th Jan 22
Editorial Decision:	15th Mar 22
Revision Received:	2nd May 22
Editorial Decision:	17th May 22
Revision Received:	19th Jun 22
Editorial Decision:	1st Aug 22
Revision Received:	30th Aug 22
Editorial Decision:	5th Oct 22
Revision Received:	13th Oct 22
Accepted:	14th Oct 22

Editor: Jingyi Hou

Transaction Report:

(Note: With the exception of the correction of typographical or spelling errors that could be a source of ambiguity, letters and reports are not edited. Depending on transfer agreements, referee reports obtained elsewhere may or may not be included in this compilation. Referee reports are anonymous unless the Referee chooses to sign their reports.)

15th Mar 2022

Dear Prof. Liu,

Thank you for submitting your work to EMBO Molecular Medicine. First of all, I would like to apologize for the slow process. We have now heard back from two of the three referees who agreed to evaluate your manuscript. Unfortunately, after a series of reminders, we did not manage to obtain a report from Referee #2. In the interest of time, I prefer to make a decision now rather than further delaying the process. As you will see below, substantial concerns have been raised by the referees, which unfortunately preclude the publication of the study in EMBO Molecular Medicine.

While Referee #3 did not raise major issues in their rather brief report, Referee #1 raised substantial technical concerns about the study and thought the quality of the presented data was not sufficient to support the main conclusions. In particular, Referee #1 rated the technical quality as "low" and indicated that they do not support the publication in EMBO Molecular Medicine. As clear and conclusive insights into a novel clinically relevant observation is key for publication in EMBO Molecular Medicine, and together with the fact that we need strong support from the referees to move on, I see no other choice than to return the manuscript to you at this point with the decision that we cannot offer to publish it.

I am very sorry that the review of your work did not result in a more favorable outcome on this occasion. Still, I hope you will not be discouraged from sending your work to EMBO Molecular Medicine in the future. In any case, thank you for the opportunity to examine this work.

Sincerely,
Jingyi

Jingyi Hou
Editor
EMBO Molecular Medicine

***** Reviewer's comments *****

Referee #1 (Comments on Novelty/Model System for Author):

The quality of the presented data and data analyses are low. Moreover, there is a lack of information about biological replicates.

Referee #1 (Remarks for Author):

Liu et al. investigated effects of reduced neural expression of Arid1a, a gene that is mutated in Coffin-Siris syndrome. How mutations in different BAF chromatin remodeling complex components, including ARID1A, lead to cognitive deficits found in Coffin-Siris syndrome remains largely unclear. To address this, the authors used four different models:

1) An acute deletion of Arid1a exon 8 in the hippocampus by injection of a Cre-expressing AAV into the hippocampus of adult Arid1afl/fl mice.
2) Arid1afl/+; Emx1-Cre+/- mice: exon 8 deletion of one Arid1a allele in Emx1-expressing cells.
3) Arid1afl/+; Nex-Cre+/- mice: exon 8 deletion of one Arid1a allele in Nex-expressing cells.
4) Neurons derived from two human ES cell lines (KO1, KO2) with homozygous ARID1A deletions.

The authors claim that reduced Arid1a expression levels in excitatory neurons lead to impaired learning and memory in mouse models, and electrophysiological defects as well as altered neurite morphologies in mice and in human ES cell-derived neurons. Moreover, they propose that reduced Arid1a levels are accompanied by lowered H3K27 acetylation and that acetate treatments can reverse the described behavioral, electrophysiological, morphological and gene expression alterations. However, the quality of the presented data is not sufficient to support several of their conclusions.

Mouse models 1), 2) and 3) were applied to investigate effects of reduced Arid1a gene expression on spatial learning and memory. For this purpose, the Morris water maze and Barnes maze tests were performed.
Models 3) and 4) were additionally used for electrophysiological, morphological, and gene expression experiments. Moreover, ChIP studies were performed with model 3).

Comments regarding animal experiments
a) In the materials and methods section, information about the AAV9 virus expressing Cre and the control virus, their production, and the intracranial injection experiments is not provided. The exact age of injected animals is missing ("young adults").
b) It is misleading to designate the animals that were injected with AAV "Arid1a deletion mice" in contrast to Arid1a cHet mice. It would be more accurate to speak about an acute deletion model.
c) Information regarding the source, background and genotyping of all mice used in this study (Arid1afl/fl, Emx-Cre, and Nex-Cre) should be provided in the materials and methods section. It would also be helpful to mention that the loxP sites flank Arid1a exon 8 as well as explain the consequences of deleting exon 8 with respect to the Arid1a protein product. It remains unclear how Arid1afl/fl control animals were generated - were these Cre-negative offspring of breedings with Cre lines or derived from pure Arid1afl/fl breedings?
d) Both, Arid1afl/+; Emx1-Cre and Arid1afl/+; Nex-Cre are referred to as cHet mice in different sections of the manuscript text and figure legends. This is misleading.
e) The breeding scheme shown in Fig. 1A cannot result in the indicated Arid1afl/fl genotype, but can only give rise to heterozygous Arid1afl/+ animals as Nex-Cre animals do not carry a floxed Arid1a allele.
f) The genotype of control mice used remains unclear. "compared to littermate controls (WT)" is written on page 6, however breeding of Arid1afl/fl with Nex-Cre+/- animals results in Arid1afl/+;Nex-Cre+/- and Arid1afl/+;Nex-Cre-/- animals. Wildtype animals would be Arid1a+/+;Nex-Cre-/-.
g) The exact age of mice used in behavioral tests is missing, nor is their sex given.
h) Motor functions should have been investigated before performing the two tests to assess if potential abnormalities are in fact memory and learning impairments and not due to decreased locomotor activity.

Comments regarding presented results, figure contents and legends
i) Fig. S1b: Uncropped immunoblot images should be shown in addition. Why are there two Arid1a protein bands?
j) Fig. S1d and f: single p-values are given, however results of multiple test days are shown.
k) Fig. 1D: Scale bars of different size are shown (CA1 panel) although they should be all 10 µm according to the figure legend. Scale bars of three of the lower panels are missing. The labeling of panels is inconsistent: cHet and WT versus and Arid1afl/+ Nex-Cre and Arid1afl/+.
l) Fig. 3: In case "n=6" / "n=4" should refer to the number of neurons analyzed, how many independent biological replicates (animals) were included?
m) Fig. 4a and c: Separate channels of the triple immunostainings should be shown. It remains unclear how the relative fluorescent intensities were analyzed. How many cells/nuclei were included in these analyses and intensities of which areas were considered?
n) Fig. 4o: How many individual neurons and how many biological replicates (animals) were analyzed?
o) Fig. S5: Nex-Cre expressing cells and their derivates were isolated by flow cytometry from Arid1afl/+;Nex-Cre; stop-tdtomato-stop mice based on Td expression in Cre-expressing cells. The authors performed RNA-seq experiments using these sorted cells, however the reference cell population remains unclear. The term "control samples" is mentioned in the manuscript text on page 9 without any further explanation.
p) Fig. 5: How many biological replicates (independent samples) were investigated?
q) Fig. 6 and results text page 13: The authors claim "compared to control vehicle-treated groups, we observed a significant increase in the number of branches (Fig. 6A-D)" ... "in acetate-treated KO neurons". However, no statistical test results are given for the intersection analyses (Fig. 6B,C,D).
r) Fig. 6: In the intersection analyses, no vehicle-treated controls (WT) are included (Fig. 6B) and control (WT) acetate-treated samples are missing as well. The ARID-KO1 and -KO2 reference samples (Fig. 6C,D) were not vehicle-treated according to the graph labels.
s) Fig. 6 E,F,G,I,J: Based on the distribution of the data points, several indicated statistical significance levels seem doubtful. Moreover, information on the number of independent biological experiments is missing.
t) Fig. 6 K,L: Wildtype (WT) qPCR measurements were intended to be used as a reference. Instead of setting their mean values to one, all individual wildtype data points are shown with relative expression levels of exactly one in these figures. This leads to false t-test results.
u) General remark on statistics: how were normal distributions of datasets assessed as a prerequisite for applying t-tests?

Referee #3 (Comments on Novelty/Model System for Author):

authors examined the role of a critical gene Arid1a in a conditional deletion mouse model. Arid1a is one of candidate genes leading to neural developmental orders. previously, there is few studies focusing on the mechanistic and behavioral defects of Arid1a gene deletion mouse. thus this work is quite unique in this aspect. authors used a conditional heterozygous deletion mouse model, which carrying deletion of Arid1a specifically in post-mitotic neurons. this models narrows down the role of Arid1a into CNS and provides critical insights for the role of Arid1a in the brain.

Referee #3 (Remarks for Author):

Authors examined the role of Arid1a in the conditional deletion mouse model. Arid1a is one of candidate genes leading to neural developmental orders. previously, there is few studies focusing on the mechanistic and behavioral defects of Arid1a gene deletion mouse. thus this work is quite unique in this aspect. authors found that deletion of Arid1a in the CNS neurons lead to various defects including synaptic transmission and neural development. interestingly, authors used a previously identified approach, acetate treatment, to rescue the behavioral defects of Arid1a cko animal. this is a thorough study providing novel and unique insights about the mechanism of Arid1a-related disorders as well as epigenetic approaches to intervene neural developmental disorders. i have some minor comments prior to recommend publication on EMM.

1. mutations of Arid1a are only part of causes for CSS. thus i suggest that authors revised the statement in introduction and briefly discuss other gene mutations of CSS.
2. since acetate is given through IP injection, more thorough analysis should be done beside body weight in order to rule out potential side effects of acetate to mouse. i suggest authors performed some further analysis to blood of mouse with acetate treatment.
3. the gene names of mouse should be italic with capital initials. authors may check the manuscript throughout.

As a service to authors, EMBO provides authors with the possibility to transfer a manuscript that one journal cannot offer to publish to another EMBO publication. The full manuscript and if applicable, reviewers reports are automatically sent to the receiving journal to allow for fast handling and a prompt decision on your manuscript. For more details of this service, and to transfer your manuscript to another EMBO title please click on Link Not Available

Please do not share this URL as it will give anyone who clicks it access to your account.

Responses to the comments

We appreciate the editors and anonymous reviewers for your in-depth comments, suggestions and corrections, which have greatly improved our manuscript. We have addressed all the comments and revised the manuscript. The point-by-point responses (in **blue**) to each comment (in ***italic***) are shown below, and all of the major revisions are highlighted in **red** in the revised manuscript.

Detailed Responses to Reviewers Comments

The comments from the reviewers are in ***italic*** and our responses are as **plain** text.

Reviewer's comments

Referee #1 (Comments on Novelty/Model System for Author):

The quality of the presented data and data analyses are low. Moreover, there is a lack of information about biological replicates.

Response: Many thanks for Referee #1's critical comments. We have now completed a substantial number of new experiments and analyses to address all the concerns raised by the reviewers. We are glad that new data are consistent and extended our original conclusion. We believe our manuscript is significantly improved.

Referee #1 (Remarks for Author):

Liu et al. investigated effects of reduced neural expression of Arid1a, a gene that is mutated in Coffin-Siris syndrome. How mutations in different BAF chromatin remodeling complex components, including ARID1A, lead to cognitive deficits found in Coffin-Siris syndrome remains largely unclear. To address this, the authors used four different models:

1) An acute deletion of Arid1a exon 8 in the hippocampus by injection of a Cre-expressing AAV into the hippocampus of adult Arid1afl/fl mice.

2) Arid1afl/+; Emx1-Cre+/- mice: exon 8 deletion of one Arid1a allele in Emx1-expressing cells.

3) Arid1afl/+; Nex-Cre+/- mice: exon 8 deletion of one Arid1a allele in Nex-expressing cells.

4) Neurons derived from two human ES cell lines (KO1, KO2) with homozygous ARID1A deletions.

The authors claim that reduced Arid1a expression levels in excitatory neurons lead to impaired learning and memory in mouse models, and electrophysiological defects as well as altered neurite morphologies in mice and in human ES cell-derived neurons. Moreover, they propose that reduced Arid1a levels are accompanied by lowered H3K27 acetylation and that acetate treatments can reverse the described behavioral, electrophysiological, morphological and gene expression alterations. However, the quality of the presented data is not sufficient to support several of their conclusions.

Mouse models 1), 2) and 3) were applied to investigate effects of reduced Arid1a gene expression on spatial learning and memory. For this purpose, the Morris water maze and

Barnes maze tests were performed.

Models 3) and 4) were additionally used for electrophysiological, morphological, and gene expression experiments. Moreover, ChIP studies were performed with model 3).

Response: We appreciate Referee #1's careful review and critical comments. We added the advantages and disadvantages discuss of mouse models as well as limitations of the present study (*Page* 14, *line* 339-344; *Page* 15, *line* 358-361, *Page* 17, *line* 404-405).

Comments regarding animal experiments

a) In the materials and methods section, information about the AAV9 virus expressing Cre and the control virus, their production, and the intracranial injection experiments is not provided. The exact age of injected animals is missing ("young adults").

Response: We deeply apologize for not providing the detailed information about the production and injection of AAV9 virus in our manuscript.

We bought the AAV9 virus from company. The AAV was prepared using triple-plasmid transfection of HEK293 cells. The main pAAV plasmid contained AAV2 ITRs, Separate Rep/Cap plasmid and the helper Plasmid provided components of the viral replication machinery and the capsid proteins of selected AAV serotypes. After HEK293 cell lysis, viral particles were purified by CsCl gradient ultracentrifugation. And rAAVs were titered by quantitative polymerase chain reaction (qPCR). The viral tools were all packaged by Brain VTA (BrainVTA Co., Ltd., Wuhan, China).

In the intracranial injection experiments, we injected stereotaxically1 μL of AAV2/9 virus (BrainVTA Co., Ltd. Company, China; Titer: > 6.15×10^{12} V.G./mL) into the hippocampus (stereotaxic coordinates from Bregma: 2.0 mm caudal, 1.2 mm lateral, 2.0 mm ventral; 2.8mm caudal, 2.0mm lateral, 1.7mm ventral) of 6-week-old ARID1A$^{fl/fl}$ mice. We have added this detailed information in our revised manuscript (*Page* 18, *line* 433-437).

b) It is misleading to designate the animals that were injected with AAV "Arid1a deletion mice" in contrast to Arid1a cHet mice. It would be more accurate to speak about an acute deletion model.

Response: We appreciate this correction. We have designated the animals that were injected with AAV "Arid1a deletion mice" as an acute deletion model in the revised manuscript (*Page 5, Line 102-116*).

c) Information regarding the source, background and genotyping of all mice used in this study (Arid1afl/fl, Emx-Cre, and Nex-Cre) should be provided in the materials and methods section. It would also be helpful to mention that the loxP sites flank Arid1a exon 8 as well as explain the consequences of deleting exon 8 with respect to the Arid1a protein product. It remains unclear how Arid1afl/fl control animals were generated - were these Cre-negative offspring of breedings with Cre lines or derived from pure Arid1afl/fl breedings?

Response: We deeply apologized for not providing the informative Materials and Methods.

The Arid1a$^{fl/fl}$ mice on a congenic C57BL/6J background (a gift from Dr. Zhong Wang at University of Michigan and Dr. Chun-sheng Han at the Institute of Zoology, Chinese Academy of Sciences) possess loxP sites flanking exon 8 of the *Arid1a* gene. Emx1-Cre (Strain #:005628, C57BL/6 background) was bought on a congenic C57BL/6J background from the Jackson Laboratory. The Nex-Cre mice (C57BL/6 background) was kindly provided from the lab of Feng-Quan Zhou, Johns Hopkins University. Mice were genotyped by PCR analysis using the genomic DNA isolated from tails. The following primers were used: flox/flox, forward (5'- TGG GCA GGA AAG AGT AAT GG -3'), reverse (5'- AAC ACC ACT TTC CCA TAG GC -3'). Emx1-Cre, transgene forward (5'- GCGGTCTGGCAGTAAAAACTATC -3'), transgene reverse (5'-GTGAAACAGCATTGCTGTCACTT -3'), internal positive control forward (5'-AAGGTGTGGTTCCAGAATCG -3'), internal positive control reverse (5'-CTCTCCACCAGA AGGCTGAG -3'). Nex-cre, Primer1 (5'-GAGTCCTGGAATCAGTCTTTTTC-3'), Primer2, (5'-AGAATGTGGAGTAGGGTGAC -3'), Primer3, (5'-CCGCATAACCAGTGAAACAG -3'). We have added the detailed information in our revised manuscript (*Page17- 18, Lines 417-427*) and in the revised supplemental data.).

We generated mice lacking Arid1a activity in excitatory neurons by crossing Arid1a$^{fl/fl}$ mice, in which exon 8 of ARID1A gene is flanked by loxP sites, with Nex-Cre mice. The Arid1a$^{fl/fl}$ mice were crossed with the excitatory neuron specific promoter driven transgenic mice Nex-Cre, then generating *Arid1a$^{fl/+}$;Nex-Cre+* (hereafter referred as cHet) and *Arid1a$^{fl/+}$;Nex-Cre-/-* (WT) mice. *Arid1a$^{fl/+}$;Nex-Cre+* mice (F1 generation) were further crossed with Arid1a$^{fl/fl}$ mice to obtain homozygous *Arid1a$^{fl/fl}$;Nex-Cre+* (hereafter referred as cKO) mice, *Arid1a$^{fl/fl}$;Nex-Cre-* mice, *Arid1a$^{fl/+}$;Nex-Cre-* (WT, as control animals in our study) mice and *Arid1a$^{fl/+}$;Nex-Cre+* (cHet) animals. In our study, we used *Arid1a$^{fl/+}$;Nex-Cre-/-* as our WT control mice. The detailed mouse breeding schematic is as follows (Figure R1), we also changed Fig.1A with new breeding schematic (see in our new Figure.1A). We have added this information in the revised manuscript (*Page 18, Lines 423-427*).

d) Both, Arid1afl/+; Emx1-Cre and Arid1afl/+; Nex-Cre are referred to as cHet mice in different sections of the manuscript text and figure legends. This is misleading.

Response: Many thanks for the correction. In our revised manuscript, we have only designated *Arid1a$^{fl/+}$;Nex-Cre* mice as cHet mice (*Page 5-6, Lines 127-128*).

e) The breeding scheme shown in Fig. 1A cannot result in the indicated Arid1afl/fl genotype, but can only give rise to heterozygous Arid1afl/+ animals as Nex-Cre animals do not carry a floxed Arid1a allele.

Response: Thanks a lot for this correction. We have modified Fig.1A with the accurate breeding scheme in our revised manuscript (*Page 18, Lines 423-427*).

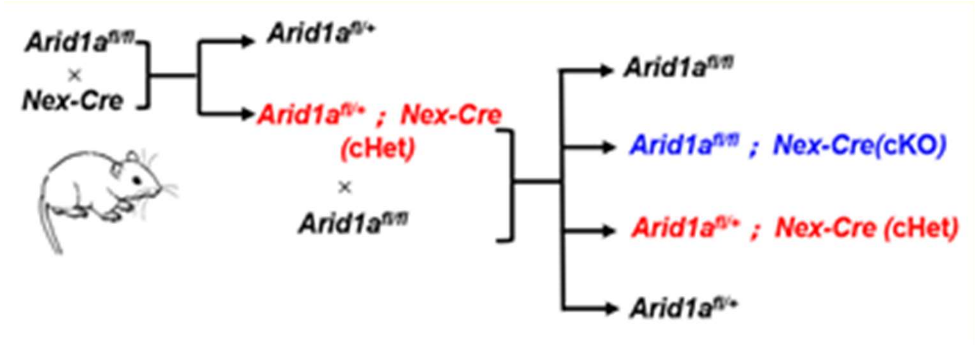

Figure R1, Revised New Figure 1A

f) The genotype of control mice used remains unclear. "compared to littermate controls (WT)" is written on page 6, however breeding of Arid1afl/fl with Nex-Cre+/- animals results in Arid1afl/+;Nex-Cre+/- and Arid1afl/+;Nex-Cre-/- animals. Wildtype animals would be Arid1a+/+;Nex-Cre-/-.

Response: Yes. Wildtype animals were *Arid1a*$^{fl/+}$*;Nex-Cre*$^{-/-}$ (*Page 5-6, Lines 127-128*) in our study.

g) The exact age of mice used in behavioral tests is missing, nor is their sex given.

Response: We deeply apologize for not providing this information in the original manuscript. Two- to three-month-old male mice were used for behaviour assays. We have added this information in the Materials and Methods section in our revised manuscript (*Page 19, line 445-449*).

h) Motor functions should have been investigated before performing the two tests to assess if potential abnormalities are in fact memory and learning impairments and not due to decreased locomotor activity.

Response: Actually, we did investigate locomotor activity using the open field test before performing the learning and memory assays. No significant difference in locomotor activity between the two genotypes, indicating that learning and memory impairments in Arid1a acute deletion (AAV-cre virus injection), *Arid1a*$^{fl/+}$*;Emx-Cre+ and Arid1a*$^{fl/+}$*;Nex-Cre+* (cHet) mice were not due to movement dysfunction. We have provided the locomotor activity data in our revised manuscript *(Page 5, line 102-106; Page 5, line 116-120; Page 6, line 135-138) and* Supplemental Fig.1DE 2AB, 3DE.

Comments regarding presented results, figure contents and legends

i) Fig. S1b: Uncropped immunoblot images should be shown in addition. Why are there two Arid1a protein bands?

Response: We have provided all the full images of immunoblot.

Figure for reviewers removed

j) Fig. S1d and f: single p-values are given, however results of multiple test days are shown.

Response: Thanks for pointing this out, we have modified in our new figures (Fig.S 1F and I).

k) Fig. 1D: Scale bars of different size are shown (CA1 panel) although they should be all 10 µm according to the figure legend. Scale bars of three of the lower panels are missing. The labeling of panels is inconsistent: cHet and WT versus and Arid1afl/+ Nex-Cre and Arid1afl/+.

Response: We have provided clear scar bars in Figure 1D.

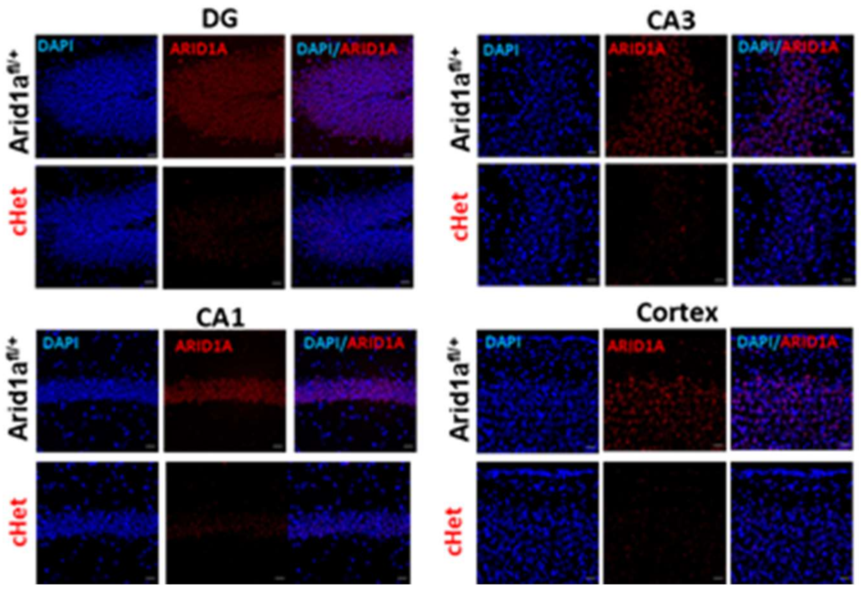

Figure R3: New Figure 1D. Immunostaining against ARID1A (red) in the dentate gyrus (DG), cortex, CA1, CA3 region of WT and cHet mice. Scale bars, 50 µm.

l) Fig. 3: In case "n=6" / "n=4" should refer to the number of neurons analyzed; how many independent biological replicates (animals) were included?

Response: We have provided both the number of neurons analyzed and the number of independent biological replicates included in our revised manuscript. We analyzed n=15 neurons from 6 WT control and n=14 neurons for 6 cHet mice. We reorganized our figures labeled with neuron numbers in the revised manuscript (Figure.3).

m) Fig. 4a and c: Separate channels of the triple immunostainings should be shown. It remains unclear how the relative fluorescent intensities were analyzed. How many cells/nuclei were included in these analyses and intensities of which areas were considered?

Response: We have provided separate channels of the triple immunostainings in Figure 4A and C. To analyse the relative fluorescent intensities, all images were taken at the same exposure time. A small area in the section that has no fluorescence was selected to measure background signal. At least 120 cells/nuclei from cortex and hippocampus area of one animal and totally from 3 animals per group were included in fluorescent intensity analyses. We have added this information in the revised manuscript *(Page 29, line 673-675)*.

A

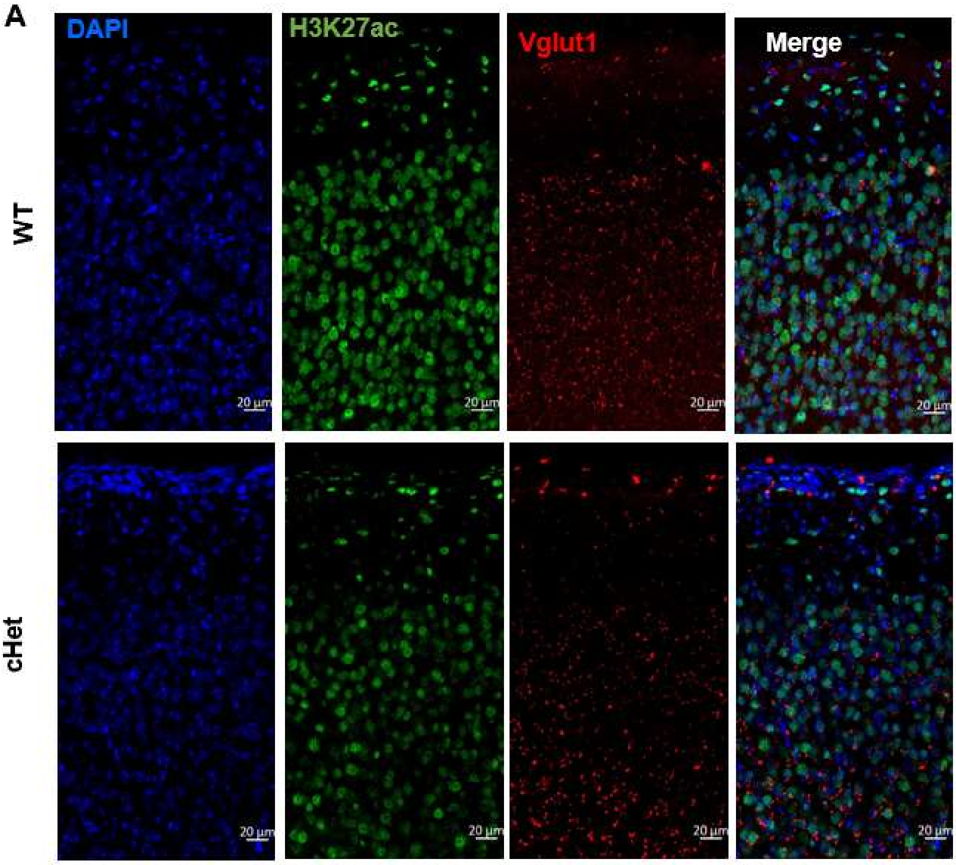
DAPI
H3K27ac
Vglut1
Merge
WT
cHet
20 µm

C

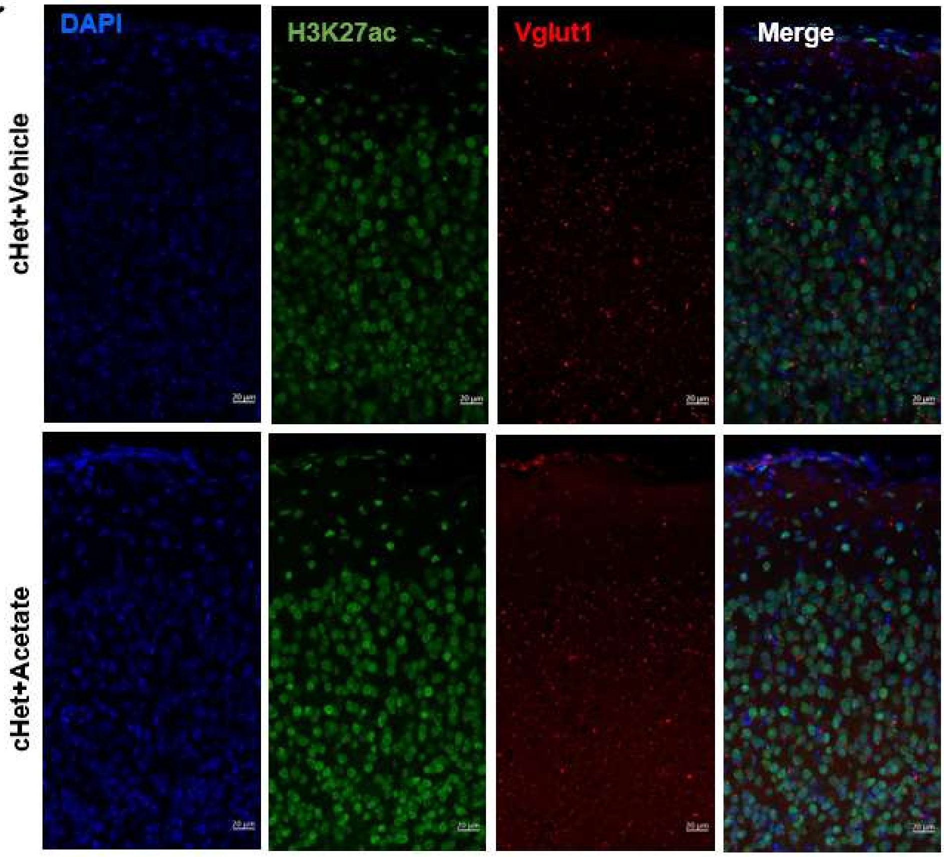
DAPI
H3K27ac
Vglut1
Merge
cHet+Vehicle
cHet+Acetate

Figure R4: New Figure 4A, Representative images of immunohistochemical staining of H3K27ac (red) and Vgult1 (green) in forebrain tissues of WT and Arid1a cHet mice. Figure 4C, Representative images of immunohistochemical staining of H3K27ac in forebrain tissues of vehicle or acetate-treated Arid1a cHet mice. Scale bars, 50 µm.

n) Fig. 4o: How many individual neurons and how many biological replicates (animals) were analyzed?

Response: Brains from 6 pairs of littermates were harvested for Golgi staining. Spines of 20 neurons were traced for each group from 4 mice. We have provided this information in the revised manuscript (P40, Line 1019).

o) Fig. S5: Nex-Cre expressing cells and their derivates were isolated by flow cytometry from Arid1afl/+;Nex-Cre; stop-tdtomato-stop mice based on Td expression in Cre-expressing cells. The authors performed RNA-seq experiments using these sorted cells, however the reference cell population remains unclear. The term "control samples" is mentioned in the manuscript text on page 9 without any further explanation.

Response: We are sorry for this missing information. We sorted Td positive cells from *Nex-cre;Td* mice as control samples. The Td+ cell population from forebrain of P28 was isolated through fluorescence-activated cell sorting (FACS). Approximately 100,000 cells were obtained in each sample. We had added this information in our revised manuscript *(Page9, Lines 227-228)*.

p) Fig. 5: How many biological replicates (independent samples) were investigated?

Response: Two biological replicates per group were investigated. We had added this information in our revised manuscript *(Page9, Lines 227-228)*.

q) Fig. 6 and results text page 13: The authors claim "compared to control vehicle-treated groups, we observed a significant increase in the number of branches (Fig. 6A-D)" ... "in acetate-treated KO neurons". However, no statistical test results are given for the intersection analyses (Fig. 6B,C,D).

Response: We have provided statistical test results in our revised manuscript (Page 13, Line314-315, New Figure 6B, C, D).

r) Fig. 6: In the intersection analyses, no vehicle-treated controls (WT) are included (Fig. 6B) and control (WT) acetate-treated samples are missing as well. The ARID-KO1 and -KO2 reference samples (Fig. 6C,D) were not vehicle-treated according to the graph labels.

Response: We have repeated this experiment and updated Figure 6 in the revised manuscript (Figure 6 B, C, D, Page 13, Line314-315).

s) Fig. 6 E,F,G,I,J: Based on the distribution of the data points, several indicated statistical significance levels seem doubtful. Moreover, information on the number of independent biological experiments is missing.

Response: We have repeated the statistical analysis and confirmed that all statistical significance levels are accurate in Figure 6.

t) Fig. 6 K,L: Wildtype (WT) qPCR measurements were intended to be used as a reference. Instead of setting their mean values to one, all individual wildtype data points are shown with relative expression levels of exactly one in these figures. This leads to false t-test results.

Response: Thanks for pointing this out, we have modified in our new Figure.6 K and L.

u) General remark on statistics: how were normal distributions of datasets assessed as a prerequisite for applying t-tests?

Response: We applied Shapiro-Wilk and Kolmogorov-Smirnov tests to assess normal distributions of datasets. All datasets passed the normality test in the present study. We have added this information in the Materials and Methods section (Page 29, Lines 675-677).

Referee #3 (Comments on Novelty/Model System for Author):
authors examined the role of a critical gene Arid1a in a conditional deletion mouse model. Arid1a is one of candidate genes leading to neural developmental orders. previously, there is few studies focusing on the mechanistic and behavioral defects of Arid1a gene deletion mouse. thus this work is quite unique in this aspect. authors used a conditional heterozygous deletion mouse model, which carrying deletion of Arid1a specifically in post-mitotic neurons. this models narrows down the role of Arid1a into CNS and provides critical insights for the role of Arid1a in the brain.
Referee #3 (Remarks for Author):
Authors examined the role of Arid1a in the conditional deletion mouse model. Arid1a is one of candidate genes leading to neural developmental orders. previously, there is few studies focusing on the mechanistic and behavioral defects of Arid1a gene deletion mouse. thus this work is quite unique in this aspect. authors found that deletion of Arid1a in the CNS neurons lead to various defects including synaptic transmission and neural development. interestingly, authors used a previously identified approach, acetate treatment, to rescue the behavioral defects of Arid1a cko animal. this is a thorough study providing novel and unique insights about the mechanism of Arid1a-related disorders as well as epigenetic approaches to intervene neural developmental disorders. i have some minor comments prior to recommend publication on EMM.

Response: Many thanks for Referee #3's positive evaluation and constructive comments.

1. mutations of Arid1a are only part of causes for CSS. thus i suggest that authors revised the statement in introduction and briefly discuss other gene mutations of CSS.

Response: We have introduced all gene mutations of CSS in our revised manuscript *(Page 3, line 54-60)*.

2. since acetate is given through IP injection, more thorough analysis should be done

beside body weight in order to rule out potential side effects of acetate to mouse. i suggest authors performed some further analysis to blood of mouse with acetate treatment.

Response: We thank the reviewer for pointing out this issue. We performed some further analysis of the mouse blood with acetate treatment after 4 weeks. We designed the two doses used in our study. Here the results showed that no significant side effects of the two doses (200mg/kg and 400mg/kg) of acetate treatment for continuous 4 weeks.

Figure for reviewers removed

3. the gene names of mouse should be italic with capital initials. authors may check the manuscript throughout.

Response: Thanks a lot for this correction. We have fixed this issue in our revised manuscript.

1st Revision - Editorial Decision **17th May 2022**

17th May 2022

Dear Chang-Mei,

Thank you for your message regarding our recent decision on your manuscript entitled "Acetate Supplementation Can Restore the Cognition Deficit Caused by ARID1A Haploinsufficiency," and thank you for sending us your revised manuscript and point-by-point responses.

We have now heard back from the two referees who agreed to evaluate your study. As you will see below, while Referee #3, who was positive in the previous round of review, remains positive, Referee #1 is still not convinced that the revised manuscript has satisfactorily addressed all the issues raised in the last round of review. Furthermore, this referee rated the technical quality as "low" and explicitly stated that they do not support the publication of the revised manuscript in EMBO Molecular Medicine.

As you may already know, our editorial policy is in principle to allow only a single round of major revision. Under these circumstances and considering the overall low level of support provided by the referees, I see no other choice than to return the manuscript with the message that we cannot offer to publish it.

I am very sorry to disappoint you again, but I hope you will soon find a suitable venue to publish your work.

Sincerely,
Jingyi

Jingyi Hou
Editor
EMBO Molecular Medicine

***** Reviewer's comments *****

Referee #1 (Comments on Novelty/Model System for Author):

The quality of the presented data and data analyses are low. Moreover, there is a lack of information about biological replicates.

Referee #1 (Remarks for Author):

Liu et al. submitted a revised version of their manuscript and provided a point-by-point response.
The lack of scientific accuracy and quality of the first and revised version raises substantial doubts about the work of these authors.

a) In the materials and methods section, information about the AAV9 virus expressing Cre and the control virus, their production, and the intracranial injection experiments is not provided. The exact age of injected animals is missing ("young adults").

Response: We deeply apologize for not providing the detailed information about the production and injection of AAV9 virus in our manuscript.
We bought the AAV9 virus from company. The AAV was prepared using tripleplasmid transfection of HEK293 cells. The main pAAV plasmid contained AAV2 ITRs, Separate Rep/Cap plasmid and the helper Plasmid provided components of the viral replication machinery and the capsid proteins of selected AAV serotypes. After HEK293 cell lysis, viral particles were purified by CsCl gradient ultracentrifugation. And rAAVs were titered by quantitative polymerase chain reaction (qPCR). The viral tools were all packaged by Brain VTA (BrainVTA Co., Ltd., Wuhan, China).
In the intracranial injection experiments, we injected stereotaxically1μL of AAV2/9 virus (BrainVTA Co., Ltd. Company, China; Titer: > 6.15 × 1012 V.G./mL) into the hippocampus (stereotaxic coordinates from Bregma: 2.0 mm caudal, 1.2 mm lateral, 2.0 mm ventral; 2.8mm caudal, 2.0mm lateral, 1.7mm ventral) of 6-week-old ARID1Afl/fl mice. We have added this detailed information in our revised manuscript (Page 18, line 433-437).

Detailed information about the three plasmids, in particular the DNA elements of the transfer plasmid, are still not given in the

materials and methods section. A bilateral injection is mentioned on page 18 - did the authors inject 0,5 ul or 1ul into each hippocampus of one animal?

c) Information regarding the source, background and genotyping of all mice used in this study (Arid1afl/fl, Emx-Cre, and Nex-Cre) should be provided in the materials and methods section. It would also be helpful to mention that the loxP sites flank Arid1a exon 8 as well as explain the consequences of deleting exon 8 with respect to the Arid1a protein product. It remains unclear how Arid1afl/fl control animals were generated - were these Crenegative offspring of breedings with Cre lines or derived from pure Arid1afl/fl breedings?
Response: We deeply apologized for not providing the informative Materials and Methods.
The Arid1afl/fl mice on a congenic C57BL/6J background (a gift from Dr. Zhong Wang at University of Michigan and Dr. Chun-sheng Han at the Institute of Zoology, Chinese Academy of Sciences) possess loxP sites flanking exon 8 of the Arid1a gene. Emx1-Cre (Strain #:005628, C57BL/6 background) was bought on a congenic C57BL/6J background from the Jackson Laboratory. The Nex-Cre mice (C57BL/6 background) was kindly provided from the lab of Feng-Quan Zhou, Johns Hopkins University.

Original publications of the Arid1afl/fl and Nex-Cre line should be cited in addition.

i) Fig. S1b: Uncropped immunoblot images should be shown in addition. Why are there two Arid1a protein bands?

Response: We have provided all the full images of immunoblot.
Figure R2 western blot confirmed AAV-mediated knockdown of Arid1a in the mouse hippocampus.

The authors still do not provide full immunoblots and I doubt the specificity of the antibody that was used for all Arid1a immunoblots and immunostainings in this manuscript. There is an unexplained double band and marker sizes are lacking. Moreover, it appears as if there are additional bands below this double band and cutting of the membranes was performed exactly through this additional lower band in A and B. This lower band is not visible in C as the gel has been run for a longer period of time so that cutting would have led to the removal of this band.
In Fig. S1B they label Arid1a bands with 270kDa and tubulin (misspelled in this figure) with 55kDa. Based on this large difference there would be no need to cut the membrane just below this double band.
In addition, it remains unclear which reference protein was in fact detected as a loading control. The immunoblots are labeled with "tublin", but no anti-tubulin antibody is given in the materials and methods section. A mouse anti-β-actin antibody is mentioned in this section (page 25).

m) Fig. 4a and c: Separate channels of the triple immunostainings should be shown. It remains unclear how the relative fluorescent intensities were analyzed. How many cells/nuclei were included in these analyses and intensities of which areas were considered?

Response: We have provided separate channels of the triple immunostainings in Figure 4A and C. To analyse the relative fluorescent intensities, all images were taken at the same exposure time. A small area in the section that has no fluorescence was selected to measure background signal. At least 120 cells/nuclei from cortex and hippocampus area of one animal and totally from 3 animals per group were included in fluorescent intensity analyses. We have added this information in the revised manuscript (Page 29, line 673-675).

According to this response, cells/nuclei from cortex and hippocampus area from one animal - three animals per genotype - were analyzed. On page 29 they write "For fluorescence intensities, whole field intensity was measured in randomly selected cortical regions (n=5 regions) with ImageJ. This is misleading and I still do not understand how many regions per mouse were in fact analyzed. Moreover, the dimensions and position of these regions are lacking. Furthermore, if one measures "whole field intensities", and not single cell intensities as done by the authors, one also includes cells that do not express Nex. The immunostainings shown in the response letter for example encompass meningeal cells.

p) Fig. 5: How many biological replicates (independent samples) were investigated?

Response: Two biological replicates per group were investigated. We had added this information in our revised manuscript (Page9, Lines 227-228).

In their response they mention "two biological replicates per group" On page 9, lines 227-228 they write: "Approximately 100,000 "228 excitatory neurons were obtained per animal (two biological replicates from two littermates)."

I still do not understand how many animals were used. Two biological replicates per group = two biological replicates from two littermates? Did they use two animals (each of them from another litter) per group, in total four animals?

Fig. 6

Only one WT hES line, but two ARID1A KO hES lines were analyzed.
Upon acetate treatment, a statistically significant difference in the # of intersections is indicated in case of the two ARID1A KO hES lines: at 60 um and 70 um (KO1, Fig. 6E) and at 40 m and 60 um (KO2, Fig. 6F). However, a trend towards an increased # of intersections is also apparent in case of the single WT hES line (Fig. 6D).
Moreover, already differences between lines of the same genotype (between the two vehicle-treated ARID1A KO hES lines and between the two acetate-treated ARID1A KO hES lines) are so large that it is doubtful that any conclusions can be from data obtained with a single WT and two KO lines.

Referee #3 (Comments on Novelty/Model System for Author):

this work used neuronal cultures and mouse models, as well as various technology including genetics and pharmacology.

Referee #3 (Remarks for Author):

authors have thoroughly revised the manuscript according to reviewer comments. I have no more comments.

Responses to the comments

We appreciate the editors and anonymous reviewers for your in-depth comments, suggestions and corrections, which have greatly improved our manuscript. We have addressed all the comments and revised the manuscript. The point-by-point responses (in **blue**) to each comment (in ***italic***) are shown below, and all of the major revisions are highlighted in **red** in the revised manuscript.

Referee #1 (Comments on Novelty/Model System for Author):
The quality of the presented data and data analyses are low. Moreover, there is a lack of information about biological replicates.
Referee #1 (Remarks for Author):
Liu et al. submitted a revised version of their manuscript and provided a point-by-point response.
The lack of scientific accuracy and quality of the first and revised version raises substantial doubts about the work of these authors.
Response: We deeply appreciate Referee #1's careful review and critical comments. Please see our detailed responses below to the concerns raised by Referee #1.

a) In the materials and methods section, information about the AAV9 virus expressing Cre and the control virus, their production, and the intracranial injection experiments is not provided. The exact age of injected animals is missing ("young adults").
Detailed information about the three plasmids, in particular the DNA elements of the transfer plasmid, are still not given in the materials and methods section. A bilateral injection is mentioned on page 18 - did the authors inject 0,5 ul or 1ul into each hippocampus of one animal?
Response: We apologize for not providing the detailed information about the production and intracranial injection of AAV2/9 virus as well as the exact age of animals in previous version of the manuscript.

AAV was produced as previously described (Bravo-Hernandez et al., 2020; Hordeaux et al., 2019). In brief, HEK293 cells were transfected with the transfer plasmid pAAV carrying EGFP (pAAV-CMV-EGFP-pA, BrainVTA) or CRE recombinase (rAAV-CMV-GFP-P2A-CRE-WPRE-bGH-pA, BrainVTA), the helper plasmid carrying adenovirus-derived genes (AD helper, Addgene plasmid #112867), and the Rep/Cap plasmid carrying AAV2 replication and AAV9 capsid genes (pAAV2/9n, Addgene plasmid #112865), which together supply all of the trans-acting factors required for AAV replication and packaging. Recombinant AAV viral particles were harvested 72 h post-transfection and purified by cesium chloride (CsCl) density gradient ultracentrifugation. Purified AAV titers were determined by real-time quantitative PCR using primers targeted the ITR (forward primer, 5'-GGAACCCCTAGTGATGGAGTT; reverse primer, 5'-CGGCCTCAGTGAGCGA).

In the intracranial injection experiments, 1μL of AAV virus (packaged by BrainVTA; Titer: > 6.15×10^{12} V.G./mL) was injected stereotaxically into each hippocampal region at 200 nL/min and a total of 2 μL virus for each 6-week-old male *Arid1a*$^{fl/fl}$ mouse (stereotaxic

coordinates from Bregma: 2.0 mm caudal, 1.2 mm lateral, 2.0 mm ventral; 2.8 mm caudal, 2.0 mm lateral, 1.7 mm ventral). We have provided the above information in the revised manuscript (*Lines445-461*).

c) Information regarding the source, background and genotyping of all mice used in this study (Arid1afl/fl, Emx-Cre, and Nex-Cre) should be provided in the materials and methods section. It would also be helpful to mention that the loxP sites flank Arid1a exon 8 as well as explain the consequences of deleting exon 8 with respect to the Arid1a protein product.
It remains unclear how Arid1afl/fl control animals were generated - were these Crenegative offspring of breedings with Cre lines or derived from pure Arid1afl/fl breedings?
Response: Many thanks for these instructive comments. We have provided the information regarding the source, background and genotyping of all mice used in the present study in Materials and Methods section of our revised manuscript *(Lines 433-440, and Table S1 in Supplementary Information).* The breeding strategy to generate experimental animals was demonstrated in Fig.1A *(Lines 1092-1093).*

Original publications of the Arid1afl/fl and Nex-Cre line should be cited in addition.
Response: We have cited original publications in our revised manuscript *(Lines 431, 433, and lines 766-768,770-771).*

i) Fig. S1b: Uncropped immunoblot images should be shown in addition. Why are there two Arid1a protein bands?
The authors still do not provide full immunoblots and I doubt the specificity of the antibody that was used for all Arid1a immunoblots and immunostainings in this manuscript. There is an unexplained double band and marker sizes are lacking. Moreover, it appears as if there are additional bands below this double band and cutting of the membranes was performed exactly through this additional lower band in A and B. This lower band is not visible in C as the gel has been run for a longer period of time so that cutting would have led to the removal of this band.
Response: We have provided uncropped immunoblot images as well as marker sizes in Supplemental Fig. S1B. To validate the ARID1A protein bands, we bought another ARID1A antibody from Sigma-Aldrich (HPA005456) and repeated the immunoblot experiment in Fig. S1B. Again, we observed multiple bands on the Western blot, indicating that both antibodies were not specific for detecting ARID1A of tissue samples. We speculate that multiple bands may result from the detection of the protein during turnover or of non-specific background immunostaining. Based on the apparent molecular mass (270 kDa) of ARID1A protein, we firmly believe that the bands we selected for analysis are correct.

In Fig. S1B they label Arid1a bands with 270kDa and tubulin (misspelled in this figure) with 55kDa. Based on this large difference there would be no need to cut the membrane just below this double band.
Response: Thanks a lot for the correction. We have fixed this issue in the revised manuscript

(Supplemental Fig. S1B).

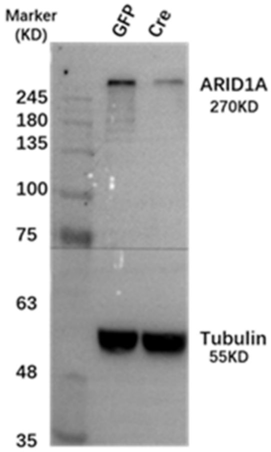

Supplemental Fig. 1B. Representative images of Western blot for ARID1A. ARID1A expression is downregulated in hippocampus after AAV-Cre viral injection. Tubulin is used as a loading control.

In addition, it remains unclear which reference protein was in fact detected as a loading control. The immunoblots are labeled with "tublin", but no anti-tubulin antibody is given in the materials and methods section. A mouse anti-β-actin antibody is mentioned in this section (page 25).

Response: Many thanks for pointing out this typo. We used Tubulin as a loading control. We have corrected the error and added the information of anti-tubulin antibody (HRP Conjugated,1:5000, BE3312, EASYBIO) in the revised manuscript (*Lines 592-593*).

m) Fig. 4a and c: Separate channels of the triple immunostainings should be shown. It remains unclear how the relative fluorescent intensities were analyzed. How many cells/nuclei were included in these analyses and intensities of which areas were considered?

According to this response, cells/nuclei from cortex and hippocampus area from one animal - three animals per genotype - were analyzed. On page 29 they write "For fluorescence intensities, whole field intensity was measured in randomly selected cortical regions (n=5 regions) with ImageJ. This is misleading and I still do not understand how many regions per mouse were in fact analyzed. Moreover, the dimensions and position of these regions are lacking. Furthermore, if one measures "whole field intensities", and not single cell intensities as done by the authors, one also includes cells that do not express Nex. The immunostainings shown in the response letter for example encompass meningeal cells.

Response: We have provided separate channels of the triple immunostainings in Supplemental Figure 5B as well as the method for quantifying the relative fluorescent intensities in the revised manuscript (*Lines 683-693*). To confirm all the cells analysed were excitatory neurons, we performed co-immunostaining of H3K27ac with an excitatory neuron

marker Vglut1. Only Vglut1 positive cells were selected to measure their relative fluorescent intensities. To determine the relative fluorescent intensities, Fluorescence images were captured at the same exposure time using the Zeiss LSM880 confocal microscope. Area, integrated density and mean grey values of at least 120 randomly selected nuclei in the cortex (150-400 μm from the pia mater) from 3 animals per group were measured. The mean fluorescence of small areas that had no fluorescence was calculated as the background reading for every image. The corrected total cell fluorescence was determined as the following equation: the Corrected Total Cell Fluorescence = Integrated Density – (Area of Selected Cell x Mean Fluorescence of Background readings). We have added the above information in our revised manuscript (*Lines 683-693*).

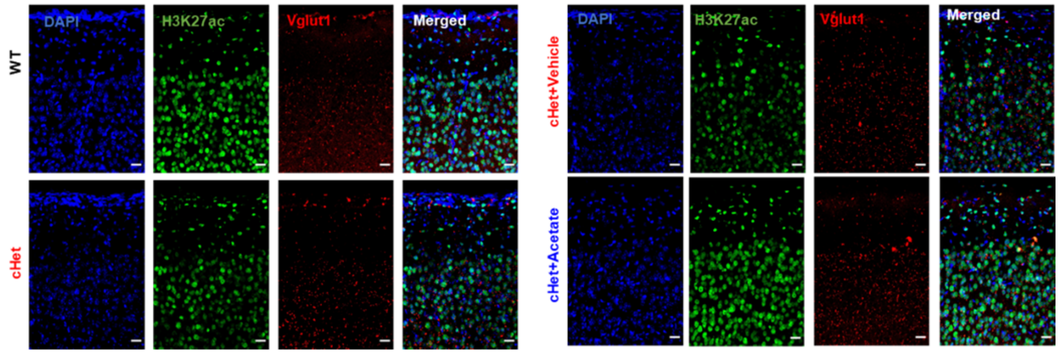

Supplemental Fig. 5B. Separate channels of the triple immunostainings staining of DAPI (blue), H3K27ac(green) and Vglut1(red) in forebrain tissues of Arid1a$^{fl/+}$ and cHet mice, respectively. IF staining were carried out on 40-μm thick floating sections. Relative fluorescence intensities of H3K27ac were reduced upon the loss of Arid1a in the cortex. Scale Bar, 20μm.

p) Fig. 5: How many biological replicates (independent samples) were investigated? In their response they mention "two biological replicates per group" On page 9, lines 227-228 they write: "Approximately 100,000 "228 excitatory neurons were obtained per animal (two biological replicates from two littermates)."
I still do not understand how many animals were used. Two biological replicates per group = two biological replicates from two littermates? Did they use two animals (each of them from another litter) per group, in total four animals?

Response: We deeply apologized for not clearly indicating the size of independent samples. Yes, we used two animals (each of them from another litter) per group, in total four animals for RNA-seq. We have revised "Two biological replicates per group" to "2 mice from 2 littermates for each group" in the revised manuscript *(line231 and New Figure 6).*

Fig. 6
Only one WT hES line, but two ARID1A KO hES lines were analyzed.
Upon acetate treatment, a statistically significant difference in the # of intersections is indicated in case of the two ARID1A KO hES lines: at 60 um and 70 um (KO1, Fig. 6E) and at 40 m and 60 um (KO2, Fig. 6F). However, a trend towards an increased # of intersections is also apparent in case of the single WT hES line (Fig. 6D).

Moreover, already differences between lines of the same genotype (between the two vehicle-treated ARID1A KO hES lines and between the two acetate-treated ARID1A KO hES lines) are so large that it is doubtful that any conclusions can be from data obtained with a single WT and two KO lines.

Response: To our knowledge, H9 is one of the most widely distributed and researched human ES cell lines on the NIH Human Embryonic Stem Cell Registry. For generating CRISPR mutation of specific gene in H9-ESCs, gene-edited H9-ESC clones and a control un-edited H9-clone are usually derived from the same parental H9-ESC clone (e.g., O'Loghlen et al., 2012; Chung et al., 2018; Yang et al., 2019; Rong et al., 2014). In our cell culture experiments, each cell line was seeded in 3 independent wells per group, and all experiments were repeated three times independently for data analysis. We totally agree with the review that more biological replicates gene-edited from different hESC lines would be better to maximize statistical power and inference capability. We have provided the information of replicates *(Lines1074-1079)*.and discussed the limitation of our present study in the revised manuscript (*Lines411-417*).

After generating ARID1A-KO1 and KO2 hESC clones by CRISPR/Cas-9 technology, we examined whether loss of ARID1A affects the morphology of hESC-derived neurons at day 40 of neural differentiation. We found that there were substantial decreases in total dendritic length ($P < 0.001$) and numbers of branches ($P < 0.001$), ends ($P < 0.05$) and nodes ($P < 0.001$) in ARID1A KO hESC-derived neurons compared to WT neurons (ANOVA), and that there was no difference in total dendritic length ($P = 0.118$) and numbers of branches ($P = 0.118$), ends ($P = 0.435$) or nodes ($P = 0.504$) between ARID1A KO1 and KO2 hESC-derived neurons (unpaired two-tailed *t*-test; Supplemental Fig.8A).

In the acetate treatment experiment, we also did not observe any obvious difference in the average number of intersections between *ARID1A* KO1 and KO2 neurons treated with vehicle ($P = 0.097$; unpaired two-tailed *t*-test). Upon acetate treatment, although a trend towards an increased number of intersections at the distance of 40-90 µm from soma was apparent, there was no significant difference in the number of intersections between vehicle-treated and acetate-treated WT groups ($P = 0.203$; unpaired two-tailed *t*-test). However, both ARID1A KO1 and KO2 hESC-derived neurons did have greater numbers of intersections upon acetate treatment, compared with that of vehicle-treated neurons (KO1: acetate vs vehicle, $P < 0.01$; KO2: acetate vs vehicle, $P < 0.05$; unpaired two-tailed *t*-test). These results strongly support that acetate could improve the morphological deficits in human ARID1A KO neurons.

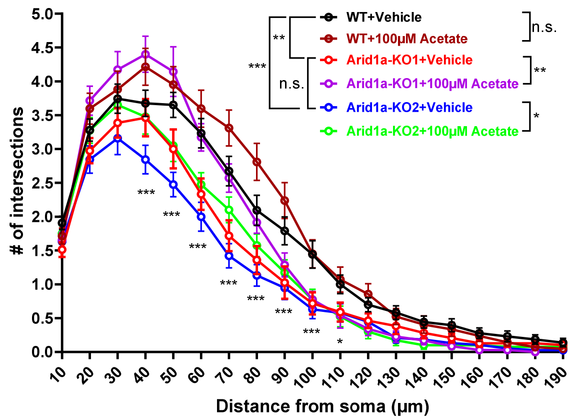

Figure 7C (new). Sholl intersection profiles of neurons treated with vehicle or acetate at day 40 of neural differentiation (n = at least 34 neurons for each group from 3 independent experiments). n.s., non-significant; *P < 0.05, ***P < 0.001; ANOVA.

Referee #3 (Comments on Novelty/Model System for Author):
this work used neuronal cultures and mouse models, as well as various technology including genetics and pharmacology.
Referee #3 (Remarks for Author):
authors have thoroughly revised the manuscript according to reviewer comments. I have no more comments.

Response: We appreciate Referee #3's tremendous efforts to support and improve our work.

References

Ana O'Loghlen, Ana M Muñoz-Cabello, Alexandre Gaspar-Maia, Hsan-Au Wu, Ana Banito, Natalia Kunowska, Tomas Racek, Helen N Pemberton, Patrizia Beolchi, Fabrice Lavial, Osamu Masui, Michiel Vermeulen, Thomas Carroll, Johannes Graumann, Edith Heard, Niall Dillon, Veronique Azuara, Ambrosius P Snijders, Gordon Peters, Emily Bernstein, Jesus Gil. MicroRNA regulation of Cbx7 mediates a switch of Polycomb orthologs during ESC differentiation. Cell Stem Cell. 2012 Jan 6;10(1):33-46. doi: 10.1016/j.stem.2011.12.004.

Hachung Chung 1, Jorg J A Calis 2, Xianfang Wu 1, Tony Sun 1, Yingpu Yu 1, Stephanie L Sarbanes 1, Viet Loan Dao Thi 1, Abigail R Shilvock 1, H-Heinrich Hoffmann 1, Brad R Rosenberg 3, Charles M Rice. Human ADAR1 Prevents Endogenous RNA from Triggering Translational Shutdown. *Cell*. 2018 Feb 8;172(4):811-824.e14. doi: 10.1016/j.cell.2017.12.038.

Xiaoyang Yang, Beisi Xu, Brett Mulvey, Myron Evans, Samuel Jordan, Yong-Dong Wang, Vishwajeeth Pagala, Junmin Peng, Yiping Fan, Arishna Patel, Jamy C Peng. Differentiation of human pluripotent stem cells into neurons or cortical organoids requires transcriptional co-regulation by UTX and 53BP1. *Nature Neuroscience*. 2019 Mar;22(3):362-373. doi:

10.1038/s41593-018-0328-5.

Zhili Rong, Meiyan Wang, Zheng Hu, Martin Stradner, Shengyun Zhu, Huijuan Kong, Huanfa Yi, Ananda Goldrath, Yong-Guang Yang, Yang Xu, Xuemei Fu. An effective approach to prevent immune rejection of human ESC-derived allografts. Cell Stem Cell. 2014 Jan 2;14(1):121-30. doi: 10.1016/j.stem.2013.11.014.

1st Aug 2022

Dear Chang-Mei,

Thank you for your message regarding our recent decision on your manuscript entitled "Acetate Supplementation Can Restore the Cognition Deficit Caused by ARID1A Haploinsufficiency," and thank you for sending us your revised manuscript and point-by-point responses. I have now discussed your revisions with the other team members, and I have also sought advice from a new expert (Referee #4) for a second opinion. As you will see from the comments below, Referee # 4 pointed out several remaining issues that need to be addressed. Given these comments, we feel that we can consider a revision of your manuscript only if you can address the following issues:

- Referee #4's concerns regarding the Points i and m raised by Referee# 1.

- Importantly, point p (regarding n=2 in the omics studies) must be carefully and satisfactorily addressed, and additional analysis needs to be provided to support that n=2 is sufficient in this case (Referee #4 made specific suggestions in this regard).

Please note that our editorial policy allows in principle a single round of major revision. In this exceptional case, we have already allowed you to submit the revised version after the manuscript has been rejected; therefore, please be aware that it is essential to provide responses to the referee's comments that are as complete as possible, as the acceptance or rejection of the manuscript will depend on this final round of review.

If you feel you can satisfactorily deal with these points, you may wish to submit a revised version of your manuscript. Please attach a covering letter giving details of how you have handled each of the points raised by the referees. A revised manuscript will be again subject to review, and you probably understand that we can give you no guarantee at this stage that the eventual outcome will be favorable.

Please read below for crucial editorial formatting and consult our author's guidelines for proper formatting of your revised article for EMBO Molecular Medicine.

Use this link to login to the manuscript system and submit your revision: https://embomolmed.msubmit.net/cgi-bin/main.plex

Yours sincerely,
Jingyi

Jingyi Hou
Editor
EMBO Molecular Medicine

***** Reviewer's comments *****

Referee #4 (Comments on Novelty/Model System for Author):

N =2 is would not be acceptable for such an RNA-seq-experiment. Thus, I checked the GEO accession number the authors had provided. You may want to have this doublechecked but I understand that for all of the experiments they have only n=2/group. Such type of data is not usable for the community and indeed raised questions about the quality of the interpretation. See general remark.

Referee #4 (Remarks for Author):

THis is a revised version of a manuscript. One reviewer is not satisfied with the revision.

Here are my thoughts on his/her comments in light of the study and specifically the issues related to "low quality":

General:
I think all the points reviewer 1 has raised are all valid. Most points of critique are in my view issues that are not too serious in a way that would question the overall quality of the data.

In essences, the current comments are about missing information about the age of animals, details on experimental procedures and raw data. Nevertheless reviewer 1 has "doubts about the work of these authors".

I would not agree with all of this issues as you will see below but reviewer 1 has relevant arguments for points i and m. However, I would not see them as a reason for rejection. What I find an important issue is point p raised by reviewer 1. The fact that the authors even in the revision do not come up with a clear statement about the n used for sequencing is not good. I also did not get it from the revised manuscript. Only when going the GEO database you see that all of their experiments are based on n=2. Such data is not usable by the community and interpretations are at least questionable. In my view any study that investigates gene-expression at the genome-wide level with n=2 is meaningless and should not be published. There are different guidelines our there and several appears have addresses this issue. None of them come to the conclusion that n=2 is a sound experimental design. To be fair, you may give the authors a chance to convince us otherwise and show a power-calculation and for example a PCA analysis to demonstrate that the 2 samples per group exhibit almost no variability amongst them and are yet substantially different when comparing experimental conditions.

Below I go through the comments one by one:

Reviewer 1, point a.

To provide information about the virus production is important and has now been provided in sufficient detail.

Reviewer 1, point c.
In my view the authors have sufficiently addressed this issue. Anyways, all mouse lines used for breeding have been previously

published.

Reviewer 1, point i.
The authors provide now the uncropped immunblot image as requested. However, it is true that this is not fully addressing the point the reviewer made, since the membrane was cut to perform the immublot with ARID1A and tubulin. This is common practice if gthe antibodies were raised in the same species, so its better to use for example mouse and rabbit antibodies on the same membrane. I guess the authors could have easily used a different loading control. The ARID1A antibody is rabbit. The authors do not provide the information about the anti-tubulin antibody and when searching for it in the internet I could not find the antibody (BE3312, EASYBIO). This antibody has been used in studies until 2019, but now I did not even find the company anymore. To be fair the authors should clarify this issue.

Reviewer 1, point m.
Reviewer 1 questions the quantification of the immuonstaining. Such data is anyways semi-quantitative at best. However, to use a synaptic protein (VGlut1) to identify a neuron in which in the nucleus H3K27ac is changing is simply not possible in my view. However, the authors back up their data by immunblot findings. I think, this point could easily be clarified by the authors.

Reviewer 1, point p.
Reviewer 2 is right. I also do not understand from the text how many mice were used.
N =2 is would not be acceptable for such an experiment. Thus, I checked the GEO accession number the authors had provided. You may want to have this doublechecked but I understand that for all of the experiments they have only n=2/group. Such type of data is not usable for the community and indeed raised questions about the quality of the interpretation. See general remark.

Reviewer 1, point "Fig. 6".
I understand that the hES cell line was used to generate two lines with ARID1 knock down. In my view this experimental design is OK.
About the statistics and the question about differences amongst the baseline and acetate treated WT and knock down groups, I think the info is OK. The authors first run an ANOVA and then t-Test which seems sound to me.

Own remark.
The references to the line numbers in the rebuttal letter do not match the lines in the text. I guess this could be an editing issue when the EMBO server generates the PDF file.

Responses to the comments

We appreciate the editors and anonymous reviewers for your in-depth comments, suggestions and corrections, which have greatly improved our manuscript. We have addressed all the comments and revised the manuscript. The point-by-point responses (in **blue**) to each comment (in ***italic***) are shown below, and all of the major revisions are highlighted in **red** in the revised manuscript.

Referee #4 (Comments on Novelty/Model System for Author):

N =2 is would not be acceptable for such an RNA-seq-experiment. Thus, I checked the GEO accession number the authors had provided. You may want to have this doublechecked but I understand that for all of the experiments they have only n=2/group. Such type of data is not usable for the community and indeed raised questions about the quality of the interpretation. See general remark.

Referee #4 (Remarks for Author):

THis is a revised version of a manuscript. One reviewer is not satisfied with the revision.

Here are my thoughts on his/her comments in light of the study and specifically the issues related to "low quality":

General:
I think all the points reviewer 1 has raised are all valid. Most points of critique are in my view issues that are not too serious in a way that would question the overall quality of the data.

In essences, the current comments are about missing information about the age of animals, details on experimental procedures and raw data. Nevertheless reviewer 1 has "doubts about the work of these authors".

I would not agree with all of this issues as you will see below but reviewer 1 has relevant arguments for points i and m. However, I would not see them as a reason for rejection. What I find an important issue is point p raised by reviewer 1. The fact that the authors even in the revision do not come up with a clear statement about the n used for sequencing is not good. I also did not get it from the revised manuscript. Only when going the GEO database you see that all of their experiments are based on n=2. Such data is not usable by the community and interpretations are at least questionable. In my view any study that investigates gene-expression at the genome-wide level with n=2 is meaningless and should not be published. There are different guidelines our there and several appears have addresses this issue. None of them come to the conclusion that n=2 is a sound experimental design. To be fair, you

may give the authors a chance to convince us otherwise and show a power-calculation and for example a PCA analysis to demonstrate that the 2 samples per group exhibit almost no variability amongst them and are yet substantially different when comparing experimental conditions.

Response: We greatly appreciate the reviewer's support and the detailed and constructive comments. Our study sampled two animals (each mouse was from a different litter) for each group in RNA-sequencing. According to the reviewer's instructive advices, we performed a principal components analysis (PCA) of the transcriptomes to examine whether the 2 samples per group exhibit almost no variability amongst them and are yet substantially different from another group. The PCA result demonstrated that the *Arid1a* cHet group and the control group were substantially different (PC1,99.4928% variance), while there was almost no variability across the replicates in each group (PC2, 0.3590% variance) (Figure EV3B), indicating that the variation in gene expression comes from "between" groups. Since the expression levels of selected dysregulated genes in cHet group were then validated through RT-PCR (Fig.6D, n=4). Therefore, we believe that our RNA-seq data should be useful in searching for candidate genes for further exploration. We have provided the PCA result (Fig EV3B) as well as the limitation of the present study in our revised manuscript (Fig EV3B, Lines 233-238).

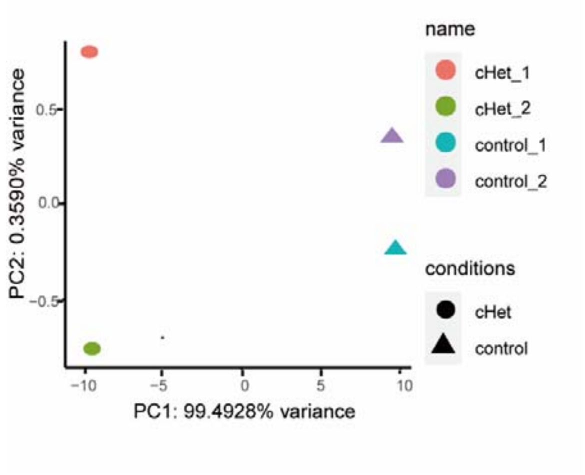

Figure EV3B. The principal component analysis (PCA) of RNA-seq data.

Below I go through the comments one by one:

Reviewer 1, point a.

To provide information about the virus production is important and has now been provided in sufficient detail.

Reviewer 1, point c.

In my view the authors have sufficiently addressed this issue. Anyways, all mouse lines used for breeding have been previously published.

Response: We thank the reviewer for the positive evaluation of our previous response to these issues.

Reviewer 1, point i.

The authors provide now the uncropped immunblot image as requested. However, it is true that this is not fully addressing the point the reviewer made, since the membrane was cut to perform the immublot with ARID1A and tubulin. This is common practice if gthe antibodies were raised in the same species, so its better to use for example mouse and rabbit antibodies on the same membrane. I guess the authors could have easily used a different loading control. The ARID1A antibody is rabbit. The authors do not provide the information about the anti-tubulin antibody and when searching for it in the internet I could not find the antibody (BE3312, EASYBIO). This antibody has been used in studies until 2019, but now I did not even find the company anymore. To be fair the authors should clarify this issue.

Response: We are sorry for not fully addressing the issue of immunblot image. The antibody against beta-Tubulin is conjugated with HRP (Catlog NO. BE3312, EASYBIO). It is raised in mouse and commercially available at the EASYBIO website (http://www.bioeasytech.com/product/2381.html?goods_id=4269). We bought ARID1A antibody from Sigma-Aldrich (HPA005456). The host species for anti-ARID1A is rabbit.

In the last round of revision, we provided uncropped immunoblot images as well as marker sizes in Fig EV1B (Response Figure 1). To validate the ARID1A protein bands, we repeated the immunoblot experiment in Fig EV1B. Again, we observed multiple bands on the Western blot, indicating that both antibodies were not specific for detecting ARID1A of tissue samples. We speculate that multiple bands may result from the detection of the protein during turnover or of non-specific background immunostaining. Based on the apparent molecular mass (270 kDa) of ARID1A protein, we firmly believe that the bands we selected for analysis are correct.

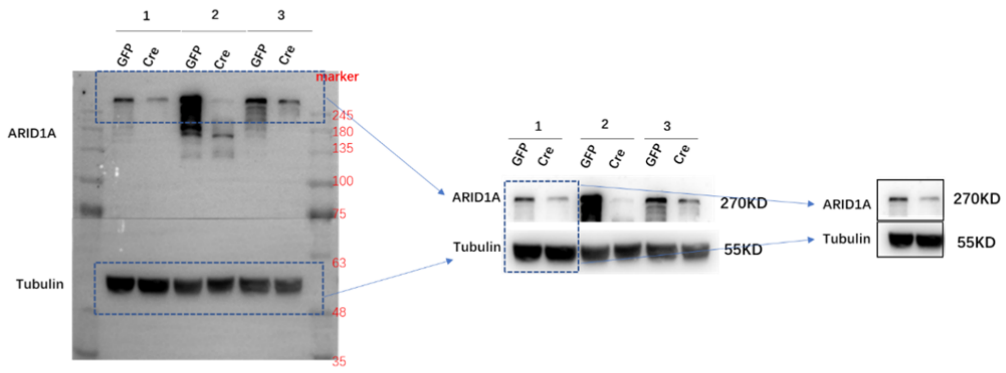

Response Figure 1. Representative western blotting images for ARID1A expression from Hippocampus, the results showed that ARID1A expression was downregulated in hippocampus after AAV-Cre injection. Tubulin is a loading control.

To confirm better the above observations, we applied both anti-beta-Tubulin (1:5000, BE3312, EASYBIO) and anti-ARID1A (1:1000, HPA005456, Sigma) on the same membrane, but had to take shorter (1s) and longer exposure time (>1min) for beta-Tubulin and ARID1A, respectively. Possible reasons for differences in exposure time include different antibody qualities and protein concentrations. As shown in the Response Figure 2, both ARID1A and

beta-Tubulin bands are visible at the exposure time of 1 min, but the signals of beta-Tubulin are too strong to discriminate different samples (left panel). At the exposure time of 1 sec, bands of Tubulin are well displayed and easy to quantify on the same membrane (right panel).

Figure for reviewers removed

Reviewer 1, point m.

Reviewer 1 questions the quantification of the immuonstaining. Such data is anyways semi-quantitative at best. However, to use a synaptic protein (VGlut1) to identify a neuron in which in the nucleus H3K27ac is changing is simply not possible in my view. However, the authors back up their data by immunblot findings. I think, this point could easily be clarified by the authors.

Response: Thank you very much for this correction. Cerebral cortex is composed of two populations of neurons: excitatory, comprising 85% of the total neurons, and inhibitory, comprising the remaining 15% (PMID: 28581480). To our knowledge, glutamatergic neurons produce glutamate, which is one of the most common excitatory neurotransmitters in the central nervous system, about 81.89 % of glutamatergic neurons co-express VGLUT1 and VGLUT2, and the others express either VGLUT1 or VGLUT2 (PMID:32562720). In Response Figure 3, VGLUT1 is found on the surface membrane of almost all the neurons in the cortex, indicating that the decreased expression of H3K27ac is mainly from excitatory neurons in Arid1a haploinsufficiency (cHet) mice. We have discussed this issue as well as the limitation of the present study in our revised manuscript (Lines381-385, 411-413).

Figure for reviewers removed

Reviewer 1, point p.

Reviewer 2 is right. I also do not understand from the text how many mice were used. N =2 is would not be acceptable for such an experiment. Thus, I checked the GEO accession number the authors had provided. You may want to have this doublechecked but I understand that for all of the experiments they have only n=2/group. Such type of data is not usable for the community and indeed raised questions about the quality of the interpretation. See general remark.

Response: Please see our above Response to this issue of sample size.

Reviewer 1, point "Fig. 6".

I understand that the hES cell line was used to generate two lines with ARID1 knock down. In my view this experimental design is OK.

About the statistics and the question about differences amongst the baseline and acetate

treated WT and knock down groups, I think the info is OK. The authors first run an ANOVA and then t-Test which seems sound to me.
Response: We thank the reviewer for the positive evaluation of our manuscript.

Own remark.
The references to the line numbers in the rebuttal letter do not match the lines in the text. I guess this could be an editing issue when the EMBO server generates the PDF file.
Response: Yes, this is an editing issue when the EMBO server generates the PDF file.

5th Oct 2022

Dear Prof. Liu,

Thank you for the submission of your revised manuscript to EMBO Molecular Medicine. We have now received the enclosed report from the referee, who was asked to re-assess it. As you will see, the referee is now supportive, and I am pleased to inform you that we will be able to accept your manuscript pending the following amendments:

1. Address the remaining concerns of the referee.

2. We updated our journal's competing interests policy in January 2022 and request authors to consider both actual and perceived competing interests. Please review the policy https://www.embopress.org/competing-interests and update your competing interests if necessary.

Please use the heading "Disclosure statement and competing interests".

3. Remove the red color font.

4. Please move 'The Paper Explained' to the main manuscript file.

5. Please remove the Author Contribution from the manuscript text, and we encourage you to use the free text boxes in the online submission system instead.

6. The funding information seems to be incomplete in the online submission system. Please ensure the entered information is consistent with that in the manuscript file.

7. In the Author Checklist, please fill out the "Ethics- Studies involving experimental animals..." box.

8. Source data:

- The "Representative images" do not seem to be source data; please remove them.
- For the Western Blot source data, please upload them in the following way:

For the main figure: it should be uploaded as one (zipped) file /figure and named as "manuscriptID_SourceDataForFigure x."

For Expanded View and Appendix figures: uploaded as a single ZIP file containing all the Source Data for Expanded View and Appendix content. Within the ZIP file, the Source Data should be included in individual folders pertaining to the figure/table that the Source Data is for.

9. I have slightly modified the synopsis text (see attached). Please let me know if it is OK like this or if you would like to introduce further modifications.

10. Our data editors have seen the manuscript and made some comments and suggestions that need answering (in track change mode, see attached). Please send back a track changes file as we will need to go through the changes.

11. As part of the EMBO Publications transparent editorial process initiative (see our Editorial at http://embomolmed.embopress.org/content/2/9/329), EMBO Molecular Medicine will publish online a Review Process File (RPF) to accompany accepted manuscripts.

In the event of acceptance, this file will be published in conjunction with your paper and will include the anonymous referee reports, your point-by-point response, and all pertinent correspondence relating to the manuscript. Let us know whether you DISAGREE with this and if you want to remove or keep any figures from it prior to publication.

Please note that the Authors checklist will be published at the end of the RPF.

Please submit your revised manuscript within two weeks. I look forward to seeing a revised form of your manuscript as soon as possible.

Sincerely,

Jingyi

Jingyi Hou
Editor
EMBO Molecular Medicine

*

***** Reviewer's comments *****

Referee #4 (Comments on Novelty/Model System for Author):

Very interesting study on the function of a gene linked to a rare congential diseases affected brain development and cognition. The same mechanisms that are likely to play a role also in sporadic brain diseases.

Referee #4 (Remarks for Author):

I like to thank the authors for addressing the remaining questions. A few final comments:

- Although n=2 is suboptimal for any OMICS experiment, the PCA analysis suggest that the differences observed between WT and ADRID1 mutant mice are rather huge and reproducible. I agree that the data is convincing.

- Similar to the RNAseq data, the authors were asked to shown PCA for the ChIPseq data that is also based on n=2, as far as I could see. Please add these data to one of the EV or main figures.

- Mention the "n" in the corresponding figure legends

- I suggest to have the final manuscript proof read by a native speaker before publication, since there still are numerous grammatical mistakes. Please also check the figures and especially the labelling of the y axes. Also check the guidelines of how to write out gene and protein names in mice and humans. This is not consistent at present.

Responses to the comments

We appreciate the editors and anonymous reviewers for your in-depth comments, suggestions and corrections, which have greatly improved our manuscript. We have addressed all the comments and revised the manuscript. The point-by-point responses (in **blue**) to each comment (in ***italic***) are shown below, and all of the major revisions are highlighted in **red** in the revised manuscript.

Referee #4 (Comments on Novelty/Model System for Author):

Very interesting study on the function of a gene linked to a rare congential diseases affected brain development and cognition. The same mechanisms that are likely to play a role also in sporadic brain diseases.

Referee #4 (Remarks for Author):

I like to thank the authors for addressing the remaining questions. A few final comments:

- Although n=2 is suboptimal for any OMICS experiment, the PCA analysis suggest that the differences observed between WT and ADRID1 mutant mice are rather huge and reproducible. I agree that the data is convincing.

Response: We thank the reviewer for the positive evaluation of our manuscript.

- Similar to the RNAseq data, the authors were asked to shown PCA for the ChIPseq data that is also based on n=2, as far as I could see. Please add these data to one of the EV or main figures.

Response: Many thanks for the constructive comment. PCA for the ChIPseq data has been shown in Figure EV3E (Lines 274-276 and 1252-1253).

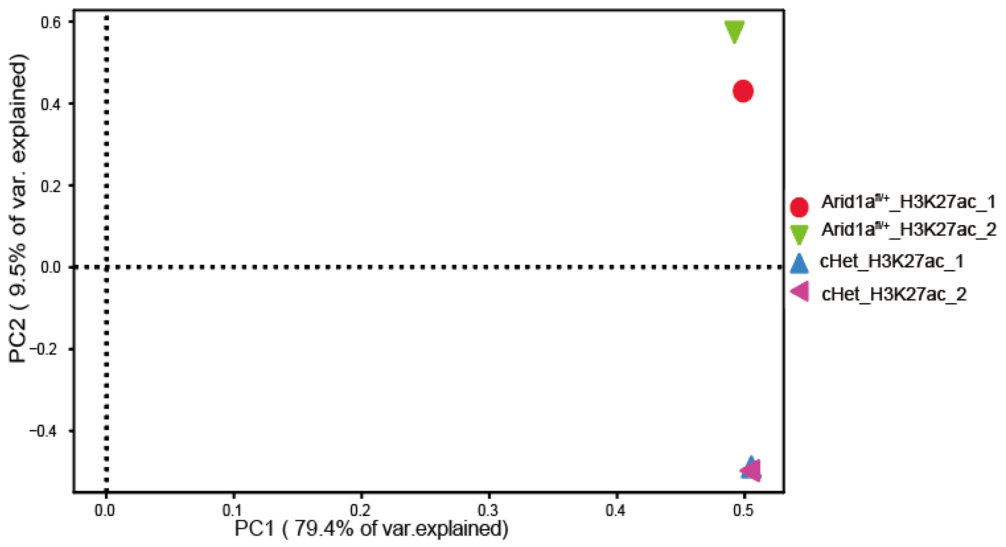

Figure EV3E. The principal component analysis (PCA) results of H3K27ac ChIP-seq data based on enrichment signals at peak regions.

- Mention the "n" in the corresponding figure legends

Response: We have provided the "n" values in the corresponding figure legends (Lines 1000, 1018, 1020, 1042,1046,1054,1058, 1068, 1073, 1108, 1116, 1227, 1229,1267, 1274,1297, and 1299 in clean manuscript).

- I suggest to have the final manuscript proof read by a native speaker before publication, since there still are numerous grammatical mistakes. Please also check the figures and especially the labelling of the y axes. Also check the guidelines of how to write out gene and protein names in mice and humans. This is not consistent at present.

Response: The final manuscript has been carefully proofread by a native English speaker. We have also checked all the names of genes and proteins in the final manuscript.

14th Oct 2022

Dear Prof. Liu,

We are pleased to inform you that your manuscript is accepted for publication and is now being sent to our publisher to be included in the next available issue of EMBO Molecular Medicine.

We would like to remind you that as part of the EMBO Publications transparent editorial process initiative, EMBO Molecular Medicine will publish a Review Process File online to accompany accepted manuscripts. If you do NOT want the file to be published or would like to exclude figures, please immediately inform the editorial office via e-mail.

Please read below for additional IMPORTANT information regarding your article, its publication and the production process.

Congratulations on your interesting work,

Jingyi

Jingyi Hou
Editor
EMBO Molecular Medicine

Follow us on Twitter @EmboMolMed
Sign up for eTOCs at embopress.org/alertsfeeds

EMBO Press Author Checklist

Corresponding Author Name: Chang-Mei Liu	
Journal Submitted to: EMBO Molecular Medicine	
Manuscript Number: EMM-2022-15795R3	

USEFUL LINKS FOR COMPLETING THIS FORM
The EMBO Journal - Author Guidelines
EMBO Reports - Author Guidelines
Molecular Systems Biology - Author Guidelines
EMBO Molecular Medicine - Author Guidelines

Reporting Checklist for Life Science Articles (updated January 2022)
This checklist is adapted from Materials Design Analysis Reporting (MDAR) Checklist for Authors. MDAR establishes a minimum set of requirements in transparent reporting in the life sciences (see Statement of Task: 10.31222/osf.io/9sm4x). Please follow the journal's guidelines in preparing your manuscript.
Please note that a copy of this checklist will be published alongside your article.

Abridged guidelines for figures
1. Data
The data shown in figures should satisfy the following conditions:
- the data were obtained and processed according to the field's best practice and are presented to reflect the results of the experiments in an accurate and unbiased manner.
- ideally, figure panels should include only measurements that are directly comparable to each other and obtained with the same assay.
- plots include clearly labeled error bars for independent experiments and sample sizes. Unless justified, error bars should not be shown for technical replicates.
- if n<5, the individual data points from each experiment should be plotted. Any statistical test employed should be justified.
- Source Data should be included to report the data underlying figures according to the guidelines set out in the authorship guidelines on Data Presentation.

2. Captions
Each figure caption should contain the following information, for each panel where they are relevant:
- a specification of the experimental system investigated (eg cell line, species name).
- the assay(s) and method(s) used to carry out the reported observations and measurements.
- an explicit mention of the biological and chemical entity(ies) that are being measured.
- an explicit mention of the biological and chemical entity(ies) that are altered/varied/perturbed in a controlled manner.
- the exact sample size (n) for each experimental group/condition, given as a number, not a range;
- a description of the sample collection allowing the reader to understand whether the samples represent technical or biological replicates (including how many animals, litters, cultures, etc.).
- a statement of how many times the experiment shown was independently replicated in the laboratory.
- definitions of statistical methods and measures:
  - common tests, such as t-test (please specify whether paired vs. unpaired), simple χ2 tests, Wilcoxon and Mann-Whitney tests, can be unambiguously identified by name only, but more complex techniques should be described in the methods section;
  - are tests one-sided or two-sided?
  - are there adjustments for multiple comparisons?
  - exact statistical test results, e.g., P values = x but not P values < x;
  - definition of 'center values' as median or average;
  - definition of error bars as s.d. or s.e.m.

Please complete ALL of the questions below.
Select "Not Applicable" only when the requested information is not relevant for your study.

Materials

Newly Created Materials	Information included in the manuscript?	In which section is the information available? (Reagents and Tools Table, Materials and Methods, Figures, Data Availability Section)
New materials and reagents need to be available; do any restrictions apply?	Yes	Materials and Methods

Antibodies	Information included in the manuscript?	In which section is the information available? (Reagents and Tools Table, Materials and Methods, Figures, Data Availability Section)
For **antibodies** provide the following information:<br>- Commercial antibodies: RRID (if possible) or supplier name, catalogue number and or/clone number<br>- Non-commercial: RRID or citation	Yes	Materials and Methods

DNA and RNA sequences	Information included in the manuscript?	In which section is the information available? (Reagents and Tools Table, Materials and Methods, Figures, Data Availability Section)
**Short novel DNA or RNA including primers, probes**: provide the sequences.	Yes	Appendix Table,Materials and Methods

Cell materials	Information included in the manuscript?	In which section is the information available? (Reagents and Tools Table, Materials and Methods, Figures, Data Availability Section)
**Cell lines:** Provide species information, strain. Provide accession number in repository **OR** supplier name, catalog number, clone number, and/**OR** RRID.	Yes	Materials and Methods
**Primary cultures:** Provide species, strain, sex of origin, genetic modification status.	Yes	Materials and Methods
Report if the cell lines were recently **authenticated** (e.g., by STR profiling) and tested for mycoplasma contamination.	Not Applicable	

Experimental animals	Information included in the manuscript?	In which section is the information available? (Reagents and Tools Table, Materials and Methods, Figures, Data Availability Section)
**Laboratory animals or Model organisms:** Provide species, strain, sex, age, genetic modification status. Provide accession number in repository **OR** supplier name, catalog number, clone number, **OR** RRID.	Yes	Materials and Methods
**Animal observed in or captured from the field:** Provide species, sex, and age where possible.	Yes	Materials and Methods
Please detail **housing and husbandry conditions**.	Yes	Materials and Methods

Plants and microbes	Information included in the manuscript?	In which section is the information available? (Reagents and Tools Table, Materials and Methods, Figures, Data Availability Section)
**Plants:** provide species and strain, ecotype and cultivar where relevant, unique accession number if available, and source (including location for collected wild specimens).	Not Applicable	
**Microbes:** provide species and strain, unique accession number if available, and source.	Not Applicable	

Human research participants	Information included in the manuscript?	In which section is the information available? (Reagents and Tools Table, Materials and Methods, Figures, Data Availability Section)
If collected and within the bounds of privacy constraints report on age, sex and gender or ethnicity for all study participants.	Not Applicable	

Core facilities	Information included in the manuscript?	In which section is the information available? (Reagents and Tools Table, Materials and Methods, Figures, Data Availability Section)
If your work benefited from core facilities, was their service mentioned in the acknowledgments section?	Not Applicable	

Design

Study protocol	Information included in the manuscript?	In which section is the information available? (Reagents and Tools Table, Materials and Methods, Figures, Data Availability Section)
If study protocol has been **pre-registered, provide DOI in the manuscript**. For clinical trials, provide the trial registration number **OR** cite DOI.	Not Applicable	
Report the **clinical trial registration number** (at ClinicalTrials.gov or equivalent), where applicable.	Not Applicable	

Laboratory protocol	Information included in the manuscript?	In which section is the information available? (Reagents and Tools Table, Materials and Methods, Figures, Data Availability Section)
Provide DOI OR other citation details if **external detailed step-by-step protocols** are available.	Not Applicable	

Experimental study design and statistics	Information included in the manuscript?	In which section is the information available? (Reagents and Tools Table, Materials and Methods, Figures, Data Availability Section)
Include a statement about **sample size** estimate even if no statistical methods were used.	Not Applicable	
Were any steps taken to minimize the effects of subjective bias when allocating animals/samples to treatment (e.g. **randomization procedure**)? If yes, have they been described?	Not Applicable	
Include a statement about **blinding** even if no blinding was done.	Yes	Behavioral assays
Describe **inclusion/exclusion criteria** if samples or animals were excluded from the analysis. Were the criteria pre-established?<br><br>If sample or data points were omitted from analysis, report if this was due to attrition or intentional exclusion and provide justification.	Not Applicable	
For every figure, are **statistical tests** justified as appropriate? Do the data meet the assumptions of the tests (e.g., normal distribution)? Describe any methods used to assess it. Is there an estimate of variation within each group of data? Is the variance similar between the groups that are being statistically compared?	Yes	Statistical Analysis

Sample definition and in-laboratory replication	Information included in the manuscript?	In which section is the information available? (Reagents and Tools Table, Materials and Methods, Figures, Data Availability Section)
In the figure legends: state number of times the experiment was **replicated** in laboratory.	Yes	Figures
In the figure legends: define whether data describe **technical or biological replicates**.	Yes	Figures

Ethics

Ethics	Information included in the manuscript?	In which section is the information available? (Reagents and Tools Table, Materials and Methods, Figures, Data Availability Section)
Studies involving **human participants**: State details of **authority granting ethics approval** (IRB or equivalent committee(s), provide reference number for approval.	Not Applicable	
Studies involving **human participants**: Include a statement confirming that **informed consent** was obtained from all subjects and that the experiments conformed to the principles set out in the WMA Declaration of Helsinki and the Department of Health and Human Services Belmont Report.	Not Applicable	
Studies involving **human participants:** For publication of **patient photos**, include a statement confirming that consent to publish was obtained.	Not Applicable	
Studies involving experimental **animals**: State details of **authority granting ethics approval** (IRB or equivalent committee(s), provide reference number for approval. Include a statement of compliance with ethical regulations.	Yes	Materials and Methods
Studies involving **specimen and field samples:** State if relevant **permits** obtained, provide details of authority approving study; if none were required, explain why.	Not Applicable	

Dual Use Research of Concern (DURC)	Information included in the manuscript?	In which section is the information available? (Reagents and Tools Table, Materials and Methods, Figures, Data Availability Section)
Could your study fall under dual use research restrictions? Please check biosecurity documents and list of **select agents and toxins** (CDC): https://www.selectagents.gov/sat/list.htm	Not Applicable	
If you used a select agent, is the security level of the lab appropriate and reported in the manuscript?	Not Applicable	
If a study is subject to dual use research of concern regulations, is the name of the **authority granting approval and reference number** for the regulatory approval provided in the manuscript?	Yes	Acknowledgment

Reporting

The MDAR framework recommends adoption of discipline-specific guidelines, established and endorsed through community initiatives. Journals have their own policy about requiring specific guidelines and recommendations to complement MDAR.

Adherence to community standards	Information included in the manuscript?	In which section is the information available? (Reagents and Tools Table, Materials and Methods, Figures, Data Availability Section)
State if relevant guidelines or checklists (e.g., **ICMJE, MIBBI, ARRIVE, PRISMA**) have been followed or provided.	Not Applicable	
For **tumor marker prognostic studies,** we recommend that you follow the **REMARK** reporting guidelines (see link list at top right). See author guidelines, under 'Reporting Guidelines'. Please confirm you have followed these guidelines.	Not Applicable	
For **phase II and III randomized controlled trials**, please refer to the **CONSORT** flow diagram (see link list at top right) and submit the CONSORT checklist (see link list at top right) with your submission. See author guidelines, under 'Reporting Guidelines'. Please confirm you have submitted this list.	Not Applicable	

Data Availability

Data availability	Information included in the manuscript?	In which section is the information available? (Reagents and Tools Table, Materials and Methods, Figures, Data Availability Section)
Have **primary datasets** been deposited according to the journal's guidelines (see 'Data Deposition' section) and the respective accession numbers provided in the Data Availability Section?	Yes	Data Availability
Were **human clinical and genomic datasets** deposited in a public access-controlled repository in accordance to ethical obligations to the patients and to the applicable consent agreement?	Not Applicable	
Are **computational models** that are central and integral to a study available without restrictions in a machine-readable form? Were the relevant accession numbers or links provided?	Not Applicable	
If publicly available data were reused, provide the respective **data citations in the reference list**.	Not Applicable	
